# Controlled human malaria infection with NF54 and 7G8 strains elicit differential antibody responses to Plasmodium falciparum peptides

**DOI:** 10.3389/fimmu.2025.1641280

**Published:** 2025-09-15

**Authors:** DeAnna J. Friedman-Klabanoff, Travis L. Jensen, Kirsten E. Lyke, Matthew B. Laurens, Joana C. Silva, Emily M. Stucke, Amed Ouattara, Olukemi O. Ifeonu, Theresa Hodges, Kara A. Moser, Casey E. Gelber, Johannes B. Goll, Stephen L. Hoffman, Jigar J. Patel, Richard S. Pinapati, John C. Tan, Gregory A. Deye, Shannon Takala-Harrison, Mark A. Travassos, Andrea A. Berry

**Affiliations:** ^1^ Center for Vaccine Development and Global Health, University of Maryland School of Medicine, Baltimore, MD, United States; ^2^ Bioinformatics and Data Science, The Emmes Company, Rockville, MD, United States; ^3^ Institute for Genome Sciences, University of Maryland School of Medicine, Baltimore, MD, United States; ^4^ Sanaria, Inc., Rockville, MD, United States; ^5^ Nimble Therapeutics, Inc., Madison, WI, United States; ^6^ Parasitology and International Programs Branch, Division of Microbiology and Infectious Diseases, National Institute of Allergy and Infectious Diseases, National Institutes of Health, Bethesda, MD, United States

**Keywords:** malaria, *Plasmodium falciparum*, controlled human malaria infection, peptide microarrays, humoral immunity to malaria, epitope

## Abstract

**Introduction:**

Extensive *Plasmodium falciparum* genetic diversity plays a role in immune evasion, and antibody responses can be strain-specific or broadly reactive depending on the epitope. Controlled human malaria infection (CHMI) allows investigation of immune responses to variant parasite proteins after a single infection with a known strain.

**Methods:**

We designed a novel diversity-reflecting peptide microarray containing 638,817 unique peptides representing 22,655 variants of 227 proteins from 23 P*. falciparum* genome assemblies and 379 field isolates. Using this array, we probed sera from 38 malaria naïve adults before and 28 days after CHMI with one of two genetically distinct *P. falciparum* strains, NF54 (n = 21) or 7G8 (n = 17). We examined fold-increase in antibody response (intensity) and cross-reactivity to protein variants (breadth). ABCPred was used to predict linear epitopes for all 227 proteins. We used MEME to identify enriched motifs in regions of high intensity or breadth, which were presumed to be potential epitopes.

**Results:**

While the two CHMI groups had similar intensity of responses to all proteins on the array, 20 proteins on the array had differential breadth of responses and participants infected with 7G8 strain had a higher breadth of responses to 17 of them. Of 543 ABCPred-predicted epitopes, 66 overlapped with MEME-identified epitopes, six of which were highly cross-reactive with >95% of peptide variants serorecognized by at least one CHMI group.

**Discussion:**

Overall, we found most antibody responses to be comparable after infection with the NF54 strain or 7G8 strain, but we saw notable differences for ~10% of proteins on the array. While many MEME-identified epitopes from highly cross-reactive proteins were asparagine rich, an epitope from PF3D7_1033200 (ETRAMP10.2) was not. Highly cross-reactive responses to ETRAMP10.2 could be further characterized and ETRAMP10.2 could be considered for inclusion in a next generation vaccine.

## 1 Introduction

Malaria continues to cause substantial morbidity and mortality worldwide, with over 263 million cases and 597,000 deaths in 2023 attributable to malaria, despite decades of research and prevention efforts (1). RTS,S/AS01 is now recommended by the World Health Organization (WHO) for widespread use in countries with moderate to high transmission of *Plasmodium falciparum* (2). But the four-dose regimen poses a logistical challenge, and supply is limited (3, 4). Also, RTS,S-associated efficacy is modest and wanes over time (5, 6). Another vaccine, R21/Matrix M, is also recommended by the WHO, which may alleviate supply chain issues (7). However, the R21/Matrix M is also given as a four-dose regimen, and the long-term efficacy and required booster frequency remain unclear (8, 9). Additional and longer lasting malaria vaccines are needed to achieve WHO goals for malaria vaccines, including >90% efficacy lasting >12 months (10). Two major impediments to a highly efficacious malaria vaccine are the complex *P. falciparum* life cycle and its extensive genetic diversity.

Vaccine candidates that target antigens from each phase of the *P. falciparum* life cycle are currently under development and may be candidates for incorporation into multi-stage vaccines (11). But thousands of additional uncharacterized proteins exist with uncertain timing of expression and propensity to elicit protective immunity. Several challenges exist to identify novel candidates for *P. falciparum* vaccines. Commonly used assays, including ELISA, generally assess only a few antigens at a time, but the *P. falciparum* genome encodes over 5,000 proteins (12). Additionally, methods such as ELISA and protein microarrays usually examine responses to whole proteins or large segments of proteins. Some malaria proteins have repetitive regions that elicit high antibody levels that correlate more with exposure to malaria than protection from infection or disease, and these immunodominant repetitive regions will drive the measured signal in an ELISA (13). Focusing on the most immunodominant region, which is effectively represented when evaluating one signal per antigen, can hinder detection of signals corresponding to informative antibody binding to less immunogenic epitopes. One example is the circumsporozoite protein (CSP), which has both an immunodominant central repeat region that induces high seroreactivity, and additional epitopes, including the binding sites of the CIS43 and L9 monoclonal antibodies (mAbs), with potent neutralizing activity (14, 15). Studying humoral responses to overlapping peptides that represent the full length of a protein permits differentiation of responses to multiple epitopes, including subdominant epitopes.

The extensive genetic diversity that is a hallmark of *P. falciparum* surface proteins facilitates host immune evasion (16–18). Determining the immune response to single antigenic variants, as occurs with most antibody-based assays, represents only partial information that does not consider locally circulating parasite strains or potential cross-reactivity between strains. Previous studies have used protein microarrays to study natural and vaccine-induced antibody responses to conveniently available laboratory strains but have not systematically assessed antigenic diversity in field isolates (19–22). Allele-specific vaccine efficacy demonstrated in other studies underscores the need to rationally design vaccines effective against antigenically diverse malaria parasites in endemic areas (23–33). Depending on the antigen and epitopes studied, antibody responses to *P. falciparum* can be broadly reactive, allele-specific, or a combination of both (i.e., broad reactivity and allele-specific functional activity). Ideally, a malaria vaccine would be broadly reactive to variants of essential parasite epitopes or would target conserved regions. Better knowledge of which proteins elicit broadly reactive versus allele-specific antibody responses could, therefore, inform development of a broadly protective malaria vaccine.

To address the challenges of finding subdominant epitopes and measuring cross-reactivity, we use high-density peptide microarrays to query diverse sequences from different strains/isolates and identify putative epitopes with amino acid-level precision. With our method, individual proteins from reference strains and field isolates are represented by 16-amino acid-long peptides that span the protein with a high degree of overlap (e.g., 15 amino acid overlap). In the current study, we measured the antibody responses elicited by single malaria infections in participants who were experimentally infected with malaria, i.e., received a controlled human malaria infection (CHMI). To disentangle strain-specific vs. cross-reactive antibody responses at the epitope level, we used high-density peptide microarrays to measure antibody responses to 227 P*. falciparum* proteins inclusive of antigenic variants in two groups of participants who received CHMI with different *P. falciparum* strains: NF54, thought to be of West African origin, and 7G8, a Brazilian clone (34). To our knowledge, these results represent the most comprehensive study to date of strain-specific and cross-reactive responses after a single episode of malaria with a known *P. falciparum* strain.

## 2 Materials and methods

### 2.1 Study participants

Serum samples originated from two CHMI dose optimization studies, NCT01546389 and NCT02780154 (35, 36). Included participants received either NF54 (n=21) or 7G8 (n=17) parasites, developed blood-stage malaria infection, received standard of care malaria treatment, and consented to future use. We probed sera from baseline (Day 1) and post-CHMI (Day 29) from these 38 participants on our diversity-reflecting peptide microarray (**Table 1**, **Supplementary Table S1**). The Institutional Review Board at the University of Maryland, Baltimore, approved the research protocols for both clinical trials, and appropriate informed consent for future use of specimens was obtained from study participants. The studies from which the samples were collected were conducted in accordance with the Declaration of Helsinki principles.

**Table 1:** Details of controlled human malaria infection procedures for participants included in this study.

Number of participants	Route^1^	Dose (sporozoites)	Strain	Method of Diagnosis^2^	Treatment^3^
3	ID	10,000	NF54	TBS	CQ
3	ID	10,000	NF54	TBS	CQ
4	ID	10,000	NF54	TBS	CQ
2	ID	10,000	NF54	TBS	CQ
2	ID	50,000	NF54	TBS	CQ
2	ID	50,000	NF54	TBS	CQ
3	DVI	800	7G8	uPCR	A/P
4	DVI	1,600	7G8	uPCR	A/P
8	DVI	3,200	7G8	uPCR	A/P
2	DVI	4,800	7G8	uPCR	A/P
5	DVI	3,200	NF54	uPCR	A/P

^1^ID, intradermal injection, DVI, direct venous injection.

^2^TBS, thick blood smear; uPCR, ultrasensitive polymerase chain reaction.

^3^CQ = 600 mg chloroquine base at time 0, 300 mg chloroquine base at hours 6, 24, and 48, A/P = atovaquone/proguanil 1000/400 mg once daily for 3 days.

ID group = Lyke KE et al. Optimizing intradermal administration of cryopreserved *Plasmodium falciparum* sporozoites in controlled human malaria infection. *Am J Trop Med Hyg.* 2015;93(6):1274-1284.

DVI group = Laurens et al. Dose-dependent infectivity of aseptic, purified, cryopreserved *Plasmodium falciparum* 7G8 sporozoites in malaria-naïve adults. *J Infect Dis*. 2019;220:1962-6.

### 2.2 Diversity-reflecting peptide microarray design

We designed a diversity-reflecting peptide array that included peptides mapping to a total of 22,655 protein variants encoded by 227 genes. The encoded proteins included known surface-exposed proteins, proteins previously observed to bind antibodies following recent malaria exposure, gametocyte and sexual stage proteins, proteins involved in metabolic pathways, and proteins included on previous peptide microarrays, with considerable overlap between these groupings. The variants originated from 23 *de novo* genome assemblies from East and West Africa and Southeast Asia (Ifeonu, Moser et al., in preparation), and alleles reconstructed from whole genome sequencing data generated in-house from 379 samples from Mali, Malawi, Brazil, Cambodia, Myanmar, Thailand, Laos, and Guinea, as well as reconstructed alleles from publicly available sequences from similar locations (**Supplementary Table S2**) (34, 37). Each protein was represented by 16-amino acid-long peptides that span the length of the protein, with a 15 amino acid overlap between consecutive peptides. A total of 638,817 unique peptides were included. We also included an additional 10,175 peptides comprised of random amino acids adapted from a collection previously used to distinguish antibody responses to influenza vaccines in triplicate (38). Details of *in situ* peptide synthesis, quality control, serum probing, and slide imaging can be found in the **Supplementary Materials**.

### 2.3 Quality control and identification of outliers

We visualized distributions of raw peptide log_2_-transformed fluorescence intensities across all samples using boxplots and cumulative distribution functions. We inspected the data for globally outlying samples using hierarchical clustering, multidimensional scaling, and principal component analysis.

### 2.4 Reference protein sequence alignments and peptide filtering

Proteins from the *P. falciparum* NF54 strain were used as reference for peptide mapping. For each protein variant, we mapped peptides against the reference protein sequence using ssearch36 (Version 36.3.8g). We excluded any peptides for which the Smith-Waterman alignment score was less than three standard deviations below the mean, the alignment length was two or more amino acids shorter than the full-length peptide, or the alignment had more than one gap in the reference or the query. Peptides and protein variants that did not have a protein mapping were excluded from the final analysis. After filtering, 520,015 (81.4%) of 638,817 peptides corresponding to 227 proteins had one or more primary reference protein mapping, including >99% of peptides that originated from NF54 or 7G8.

### 2.5 Data normalization

We executed median normalization of study data using intensities collected for 10,175 randomer peptide sequences included on the arrays. For each sample, the sample-specific median for the 10,175 randomer log_2_ fluorescence intensities was subtracted from the global median, which was the median of all sample-specific medians, to create a scaling factor for each sample. Thus, each sample specific randomer median was centered to the global randomer median FI. The full data set was then normalized by subtracting the sample-specific scaling factor from each non-randomer peptide log_2_ intensity. We tested other methods of data normalization to choose the least biased method - additional details of the other methods of normalization and scaling procedures are in the **Supplementary Materials**.

### 2.6 Sliding window smoothing procedure

Raw peptide array fluorescence intensities were log_2_ transformed. As consecutive peptides overlapped by about 94%, we expected intensities to be correlated as they may contain the same epitope. To increase the data signal-to-noise ratio, we applied a sliding window-based average smoothing procedure (39). Each peptide was represented by its midpoint (end position minus beginning position divided by two). Each amino acid position along the sequence was represented by a smoothed log_2_ fluorescence intensity equal to the average log_2_ fluorescence intensity for all peptides with a midpoint four amino acids before or after the given position. For each participant-sample and protein variant, we applied the sliding window smoothing procedure to calculate the average fluorescence intensity corresponding to each amino acid position on the reference protein sequence.

### 2.7 Serorecognition

We defined serorecognition thresholds for each peptide as the mean plus 2.5 times the standard deviation of the log_2_ fluorescence intensity at Day 1 from all participants (regardless of CHMI group). We classified a peptide as serorecognized by an individual participant if its log_2_ fluorescence intensity was greater than the serorecognition threshold for that peptide. We then applied a sliding window smoothing procedure so that at a given amino acid position along each protein, the number of serorecognized peptides with mid-points within four amino acids before or after that position were summed. We then calculated the sum of the smoothed log_2_ fluorescence intensities (SSI) across each protein variant for each sample and used it to define the serorecognition threshold for a protein variant (mean plus 2.5 times the SSI standard deviation for all baseline samples). For each post-CHMI sample, we defined a protein variant as serorecognized if the Day 29 SSI exceeded the serorecognition threshold for that protein variant. On the cohort level (CHMI group), we defined each feature (peptide and protein variant) as serorecognized by a CHMI group if the log_2_ fluorescence intensity exceeded the serorecognition threshold for >15% participants in that CHMI group.

To describe cross-reactivity (breadth of antibody response), for each protein, we captured the mean number of serorecognized peptides for each CHMI group at each amino acid position, and the maximum mean number of serorecognized peptide variants (MMNS) and its corresponding location. The MMNS and its location represents the epitope with the highest cross-reactivity (breadth of antibody response) for each protein and CHMI group.

### 2.8 Seroreactivity

Using the sliding window procedure, for each CHMI group we calculated the mean log_2_ fold change between D1 and D29 for each amino acid along the reference protein sequence for each protein variant. For each protein at each position, we averaged the log_2_ fold changes for all included protein variants, and calculated each CHMI group mean (mean of the mean of log_2_ fold changes, referring to the mean of the protein variants and the mean of the individuals in each cohort). We then summed all the position-specific mean of the mean log_2_ fold changes for each protein to represent mean (amongst protein variants and for each cohort) area under the curve for each protein (MF-AUC). We ascertained the amino acid positions and values for maximum mean of the mean fold changes (MMMF) for each protein by CHMI group. The MMMF and its location represents the epitope with the highest antibody binding (antibody intensity) for each protein and CHMI group.

### 2.9 Identification and comparison of serorecognized and seroreactive protein variants between the two CHMI groups

We assessed differences in the numbers of peptides and protein variants serorecognized after exposure to NF54 vs. 7G8 strain CHMI on the peptide and protein variant level using a Fisher’s exact test. To evaluate seroreactivity differences between the two CHMI groups, we implemented an ANOVA model in the limma R package fitted to MF-AUC including a fixed effect for CHMI group (NF54 and 7G8 strains) (40). We used a contrast to assess statistical significance of the mean MF-AUC difference between NF54 and 7G8 (H_0_: mean NF54 – mean 7G8 = 0, H_1_: mean NF54 - mean 7G8 ≠ 0, on the log_2_ scale).

We adjusted for multiple comparisons using the Benjamini-Hochberg procedure (as implemented in the R *p.adjust* function). We considered any responses with a false discovery rate (FDR) adjusted p-value < 0.05 to be proteins with differential serorecognition or seroreactivity.

### 2.10 Identification and comparison of epitopes

We used MEME (Version 5.0.5) to find dominant motifs that could represent epitopes in regions that overlapped with areas of high serorecognition or seroreactivity (MMNS or MMMF), which we then termed “serologically identified epitopes” (41). For each protein, at positions of interest (MMNS or MMMF), we used the peptide sequence per protein variant with the highest mean fluorescence intensity value at Day 29 across participants separately for each CHMI cohort as input for identifying motifs. We set the number of motifs detected per sequence to 0 or 1 occurrence (-mod zoops option) and the minimum and maximum motif width to 8 and 16, respectively (-minw and -maxw and options, respectively). The background sequence collection used for the null model included the unique set of all peptide sequences that did not overlap with either MMNS or MMMF regions. Motif discovery was analyzed separately for each CHMI group (NF54 and 7G8 strains). We identified shared motifs/epitopes between CHMI groups using the TOMTOM (Version 5.0.5) motif comparison software (42).

### 2.11 Assessment of serorecognition and seroreactivity for a set of known epitopes

We assessed serorecognition and seroreactivity for a set of literature-curated epitopes (**Supplementary Table S3**) based on a literature search and computationally predicted B-cell epitopes produced by the ABCPred prediction algorithm (**Supplementary Table S4**) (43). For ABCPred, we used a score cut off of >0.9 to define likely B-cell epitopes. We assessed the overlap between literature-curated or ABCPred-predicted and serologically identified epitopes using the TOMTOM motif comparison tool (42).

### 2.12 Summarization of epitope results across individual participants

Average epitope MMNS and MMMF for individual participants were visualized for literature-curated epitopes and serologically identified epitopes using heatmaps utilizing (1) a data driven approach (hierarchical clustering of epitopes and participants) and a (2) pre-ordered approach (participants sorted by CHMI group) to identify similar or divergent serorecognition/seroreactivity clustering profiles between CHMI groups.

### 2.13 Software

Data was analyzed using R (Version 3.6.0, 26APR2019) and R Bioconductor packages on the Ubuntu (Version 13.04) operating system.

## 3 Results

### 3.1 Quality control

**Figure 1:**
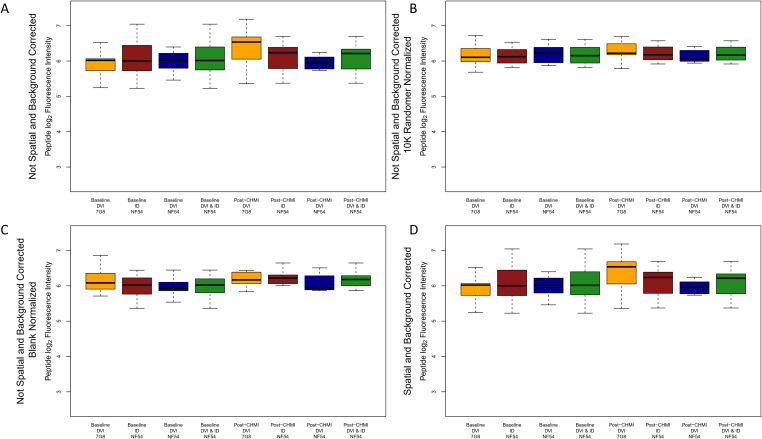
Boxplots of peptide log_2_ fluorescence intensities aggregated by timepoint, method of sporozoite delivery, and CHMI group for raw, corrected, and normalized data. Boxplots depict the median and IQR peptide log_2_ fluorescence intensity for all peptides on the array for each CHMI group at each timepoint for **(A)** raw data, **(B)** 10,175 randomer normalized data, **(C)** blank normalized data, and **(D)** spatial and background corrected data. The dark green boxes depict the aggregated median and IQR for all participants receiving NF54 strain CHMI (brown and blue boxes combined). DVI, direct venous injection; ID, intradermal injection.

The two CHMI groups (NF54 and 7G8) did not cluster into mutually exclusive groups by Euclidean distances, principal component analysis (PCA) or 1-Spearman correlation distance, and there were no strong outliers (**Supplementary Figures S1**, **S2**). Samples were run in four batches, and CHMI groups and time points were well balanced between batches. Samples did not cluster by batch in Euclidean distances, PCA or 1-Spearman correlation distance plots (**Supplementary Figure S3**). Overall, inspection of sample-specific intensities across the 638,817 peptides showed that NF54 and 7G8 CHMI groups had similar pre-CHMI median intensities, but the 7G8 CHMI group tended to have higher median intensities post-CHMI and higher fold changes relative to pre-CHMI compared to the NF54 CHMI group (aggregated by protocol and group in **Figure 1A**). Therefore, we explored different methods of normalization to mitigate bias, including spatial and background correction, blank spot normalization, and randomer normalization (**Figures 1B-D**). Randomer normalization reduced systematic differences between the groups optimally, so we used randomer normalized data for our data analysis. Boxplots of individual samples and timepoints after randomer normalization can be visualized in **Supplementary Figure S4**.

### 3.2 Serorecognition and seroreactivity

First, we assessed the breadth of responses based on serorecognition to all peptides on the array (across all proteins and variants) for the two CHMI groups. Each peptide was classified according to the strains and geographic locations in which they were originally identified, and peptides could belong in multiple groups (**Table 2**, first column). For every comparison that we evaluated, we observed higher serorecognition (breadth of responses) in the NF54 CHMI group compared to the 7G8 CHMI group (odds ratios [OR] 1.68-2.84, p <0.0001 for all, **Table 2**).

**Table 2:** Serorecognition (breadth of responses) of peptides from reference strains and field isolates.

Strain/Geographical location	Total number of peptides	Number of serorecognized peptides (7G8 CHMI group)	Number of serorecognized peptides (NF54 CHMI group)	Odds ratio (95% CI) [NF54 vs. 7G8]	P-value
NF54 (W. Africa)	278,643	5,979	10,719	1.82 (1.77,1.88)	<0.0001*
7G8 (Brazil)	258,175	5,658	10,205	1.84 (1.78,1.9)	<0.0001*
NF54-specific	20,279	344	751	2.24 (1.96,2.55)	<0.0001*
7G8-specific	7,832	267	712	2.84 (2.45,3.29)	<0.0001*
Mali	421,891	10,840	24,359	2.32 (2.27,2.38)	<0.0001*
Malawi	458,701	12,271	27,473	2.32 (2.27,2.37)	<0.0001*
Brazil	286,233	6,428	12,131	1.93 (1.87,1.99)	<0.0001*
Cambodia	333,259	8,190	16,074	2.01 (1.96,2.07)	<0.0001*
Myanmar	297,953	6,958	13,514	1.99 (1.93,2.05)	<0.0001*
Thailand	275,717	6,317	12,054	1.95 (1.89,2.01)	<0.0001*
Laos	259,996	5,778	10,383	1.83 (1.77,1.89)	<0.0001*
Guinea	252,627	5,340	9,448	1.8 (1.74,1.86)	<0.0001*
Conserved across >1 locations	348,633	8,678	18,473	2.19 (2.14,2.25)	<0.0001*
Conserved across all locations	225,394	4,722	8,028	1.73 (1.66,1.79)	<0.0001*
Conserved across all strains and locations	208,126	4,229	6,991	1.68 (1.61,1.74)	<0.0001*
Any location	629,618	17,483	39,871	2.37 (2.32,2.41)	<0.0001*
Any strain or location	638,817	17,574	40,001	2.36 (2.32,2.41)	<0.0001*

For every comparison that we evaluated, serorecognition of peptides was higher in the NF54 CHMI group compared to the 7G8 CHMI group. * indicates P-value < 0.05.

Next, we investigated serorecognition on a protein variant level, i.e., the numbers of protein variants per protein that were serorecognized by each CHMI group. Overall, we observed higher protein variant serorecognition (breadth of responses) by the 7G8 CHMI group than the NF54 CHMI group (OR 0.42, p<0.001 for protein variants from any strain or location, **Table 3**). This finding was statistically significant for the comparisons with more protein variants, i.e., most of the country specific comparisons (ORs 0.39-0.47). Of the 22,656 protein variants from 227 proteins on the array, only 364 and 347 protein variants were unique to the NF54 strain and the 7G8 strain, respectively. So, the majority of serorecognition of protein variants seen was related to cross-reactivity. No variant of the 227 proteins probed on the array was conserved across all strains and locations.

**Table 3:** Serorecognition (breadth of responses) of protein variants from reference strains and field isolates.

Strain/Geographical location	Total number of protein variants	Number of serorecognized protein variants (7G8 CHMI group)	Number of serorecognized protein variants (NF54 CHMI group)	Odds ratio (95% CI) [NF54 vs. 7G8]	P-value
NF54-specific (W. Africa)	364	22	12	0.53 (0.24,1.14)	0.1127
7G8-specific (Brazil)	347	22	11	0.48 (0.21,1.06)	0.073
Mali	7,373	572	275	0.46 (0.4,0.53)	<0.0001*
Malawi	9,813	805	357	0.42 (0.37,0.48)	<0.0001*
Brazil	1,067	73	35	0.46 (0.3,0.71)	0.0002*
Cambodia	3,620	321	157	0.47 (0.38,0.57)	<0.0001*
Myanmar	1,447	124	51	0.39 (0.27,0.55)	<0.0001*
Thailand	1,073	100	46	0.44 (0.3,0.63)	<0.0001*
Laos	400	29	12	0.4 (0.18,0.81)	0.0095
Guinea	252	17	10	0.57 (0.23,1.35)	0.2348
Conserved across >1 locations	1,735	131	86	0.64 (0.48,0.85)	0.002*
Conserved across all locations	78	3	1	0.33 (0.01,4.17)	0.6201
Conserved across all strains and locations	0	0	0	0 (0,0)	>0.9999
Any location	21,872	1,822	799	0.42 (0.38,0.45)	<0.0001*
Any strain or location	22,656	1,871	827	0.42 (0.39,0.46)	<0.0001*

Serorecognition of protein variants was higher in the 7G8 CHMI group, except for protein variants specific to NF54, specific to 7G8, specific to Guinea, or conserved across all locations, which were similar in the two CHMI groups. * indicates P-value < 0.05.

**Figure 2:**
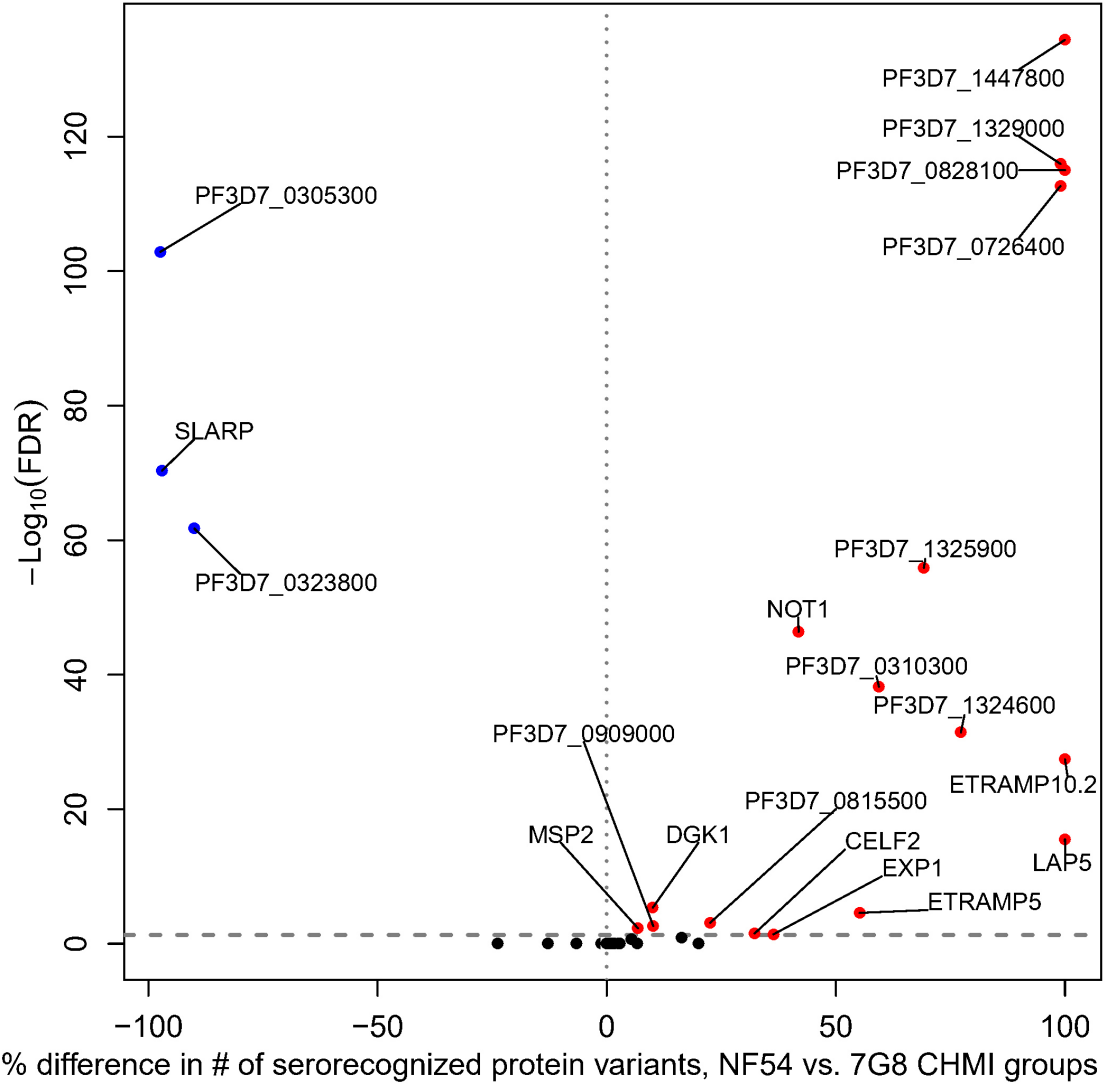
Differential breadth of responses to protein variants between NF54 CHMI group and 7G8 CHMI group. All proteins included on the array are depicted. The x-axis represents the percentage of protein variants differentially serorecognized (difference in number of serorecognized protein variants for NF54 vs. 7G8 divided by the total number of protein variants). The y-axis depicts the Fisher’s exact -log_10_ FDR-adjusted p-value. For each of the 20 proteins with a significant difference, blue dots represent those for which the NF54 CHMI group had a significantly higher percentage of serorecognized proteins and red dots represent those for which the 7G8 CHMI group had a significantly higher percentage of serorecognized proteins. Black indicates no significant difference in percentage of serorecognized protein variants between NF54 and 7G8 with many proteins overlapping. Each protein with a significant FDR-adjusted p-value is labeled with their protein ID or gene identifier.

The 7G8 CHMI group serorecognized more protein variants than the NF54 CHMI group for 17 proteins and the NF54 CHMI group serorecognized more protein variants for three proteins based on a Fisher’s Exact Test (**Figure 2**). Of the 17 proteins with more serorecognized variants by the 7G8 CHMI group, the 7G8 CHMI group serorecognized 8–230 more protein variants than the NF54 CHMI group (7-100% of total variants). For the three proteins with more serorecognized variants by the NF54 CHMI group, the NF54 CHMI group serorecognized 126–187 more protein variants than the 7G8 CHMI group (90 – 97% of total variants).

When comparing antibody levels elicited by CHMI (MF-AUC, a measure of aggregate signal across each protein), both CHMI groups showed similar seroreactivity to all 227 proteins on the array (**Supplementary Table S5**).

### 3.3 Identification and comparison of serologically identified epitopes

**Figure 3:**
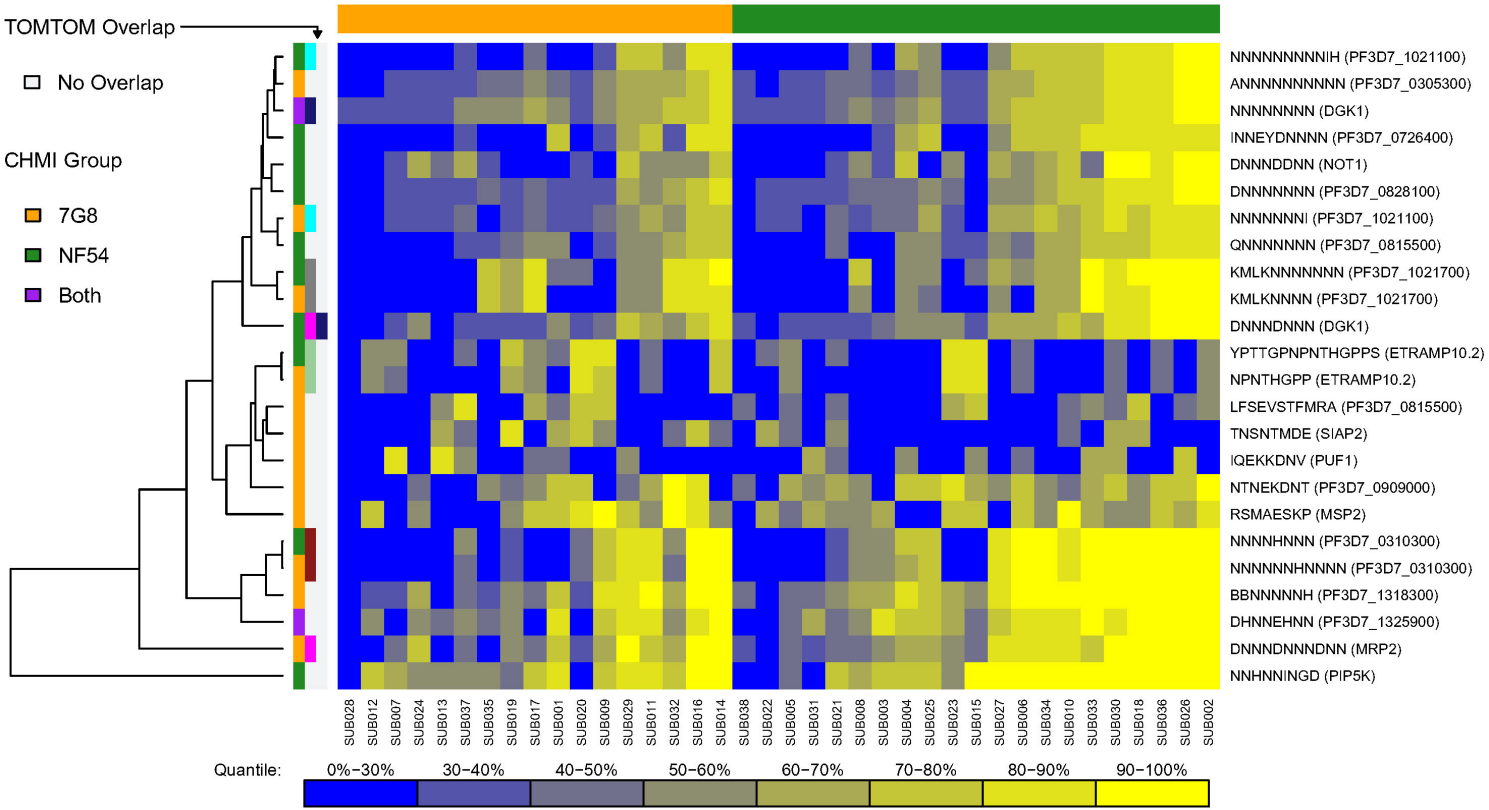
Heatmap of serologically identified epitopes identified with MEME based on breadth of responses [maximum mean peptide variant serorecognition (MMNS)]. Rows represent identified epitopes with the first column of colors to the left of the figure representing whether the epitope was identified for the 7G8 CHMI group (gold), NF54 CHMI group (green), or both (purple). The second and third column of colors indicates epitopes that overlapped between the CHMI groups using TOMTOM with light grey representing no overlap and other colors showing the epitopes that overlapped. One epitope overlapped with two others, and so shows pink in the second column and navy blue in the third, which corresponds to the two other epitopes to which it overlapped. Rows were hierarchically clustered based on complete linkage clustering of Euclidean distances between MMNS values. Each column represents the responses from a single participant in the study with higher responses represented in yellow and lower responses represented in blue. Gradient corresponding to quantiles depicted underneath the heatmap. The participants are grouped by CHMI group and ordered within group by overall response with the graphic above the graph showing which participants were from the 7G8 CHMI group (gold) and which were in the NF54 CHMI group (green). Some overlapping epitopes clustered by Euclidean distances, but some did not. Responses were overall heterogeneous between participants without obvious clustering by CHMI group.

For each CHMI group, along each protein sequence, we identified one epitope that had the maximum mean number of serorecognized peptide variants (MMNS), implying the largest breadth of responses along that protein, and one epitope that had the maximum mean of the mean fold changes (MMMF), implying the highest intensity responses along that protein. We termed these “serologically identified epitopes.” For each CHMI group, any peptide sequences mapping to the MMNS (breadth) or MMMF (intensity) were used as input for MEME to find dominant motifs in each of the 227 proteins. Using MEME, we found 16 and 14 serologically identified epitopes for the 7G8 and NF54 CHMI groups, respectively, based on breadth of responses (MMNS) (**Supplementary Table S6**). Based on TOMTOM analysis, seven of these serologically identified epitopes overlapped between the two CHMI groups with one 7G8 epitope overlapping with two NF54 epitopes (**Supplementary Table S6**). Heatmaps of individual responses segregated by CHMI group are displayed in **Figure 3**. While some of the overlapping epitopes clustered by Euclidean distances, some did not. Responses did not cluster by CHMI group and responses were overall heterogeneous between participants. Three of the overlapping serologically identified epitopes mapped to single proteins for both groups: PF3D7_1021700, PF3D7_1033200, PF3D7_1325900. The others mapped to 3–67 proteins for each group. Many of the serologically identified epitopes found are highly repetitive and contain multiple asparagine residues.

**Figure 4:**
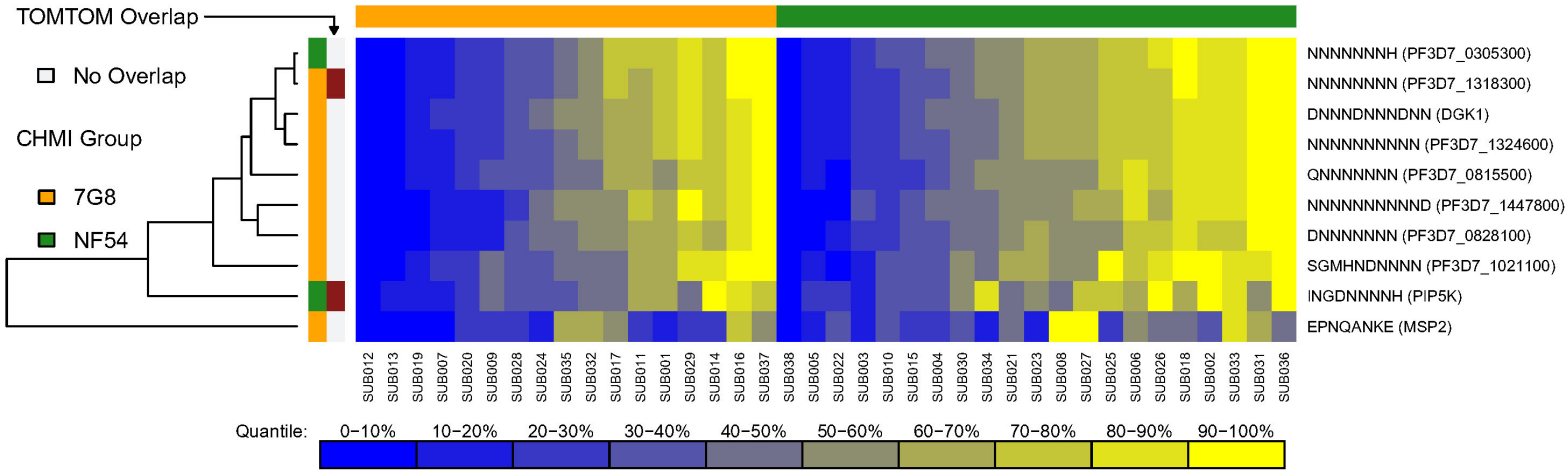
Heatmap of serologically identified epitopes identified with MEME based on intensity of responses [maximum mean of mean log_2_ fold change (MMMF)]. Rows represent identified epitopes with the first column of colors to the left of the figure representing whether the epitope was identified for the 7G8 CHMI group (gold) or NF54 CHMI group (green). The second column of colors indicates epitopes that overlapped between the CHMI groups using TOMTOM with light grey representing no overlap and maroon showing the epitopes that overlapped. Rows were hierarchically clustered based on complete linkage clustering of Euclidean distances between MMMF values. Each column represents the responses from a single participant in the study with higher responses represented in yellow and lower responses represented in blue. The participants are grouped by CHMI group and ordered within group by overall response with the graphic above the graph showing which participants were from the 7G8 CHMI group (gold) and which were in the NF54 CHMI group (green). The overlapping epitope did not cluster by Euclidean distances. Responses were overall heterogeneous between participants without obvious clustering by CHMI group.

Using the second metric, MMMF, which represents the intensity of responses, we found nine and two serologically identified epitopes for the 7G8 and NF54 CHMI groups, respectively (**Supplementary Table S7**). One epitope was shared among the two CHMI groups based on TOMTOM analysis, which mapped to 65 proteins for the 7G8 serologically identified epitope and 1 protein (PF3D7_0110600) for the NF54 serologically identified epitope. Heatmaps of individual responses segregated by CHMI group are shown in **Figure 4**. We did not see clustering of the overlapping epitope by Euclidean distances, nor did we see clustering of responses by CHMI group. Serologically identified epitopes found using maximum mean seroreactivity were repetitive and contained many asparagine residues. For all methods of ascertaining serologically identified epitopes, some serologically identified epitopes were found in multiple proteins (up to 70 proteins). These were highly repetitive epitopes with more than 50% of the epitope comprised of asparagine residues.

### 3.4 Comparison of literature-curated epitopes and ABCPred-predicted epitopes to serologically identified epitopes

We used the TOMTOM motif comparison tool to find similarities between literature-curated epitopes and serologically identified epitopes. We found one 7G8 serologically identified epitope (EPNQANKE) based on intensity of responses (MMMF) that had similarities to two literature-curated epitopes, both in merozoite surface protein 2 (MSP-2, PF3D7_0206800): EPNQANKE in the variable region and ECTDGNKE in the conserved C-terminal region (**Supplementary Table S8**).

**Figure 5:**
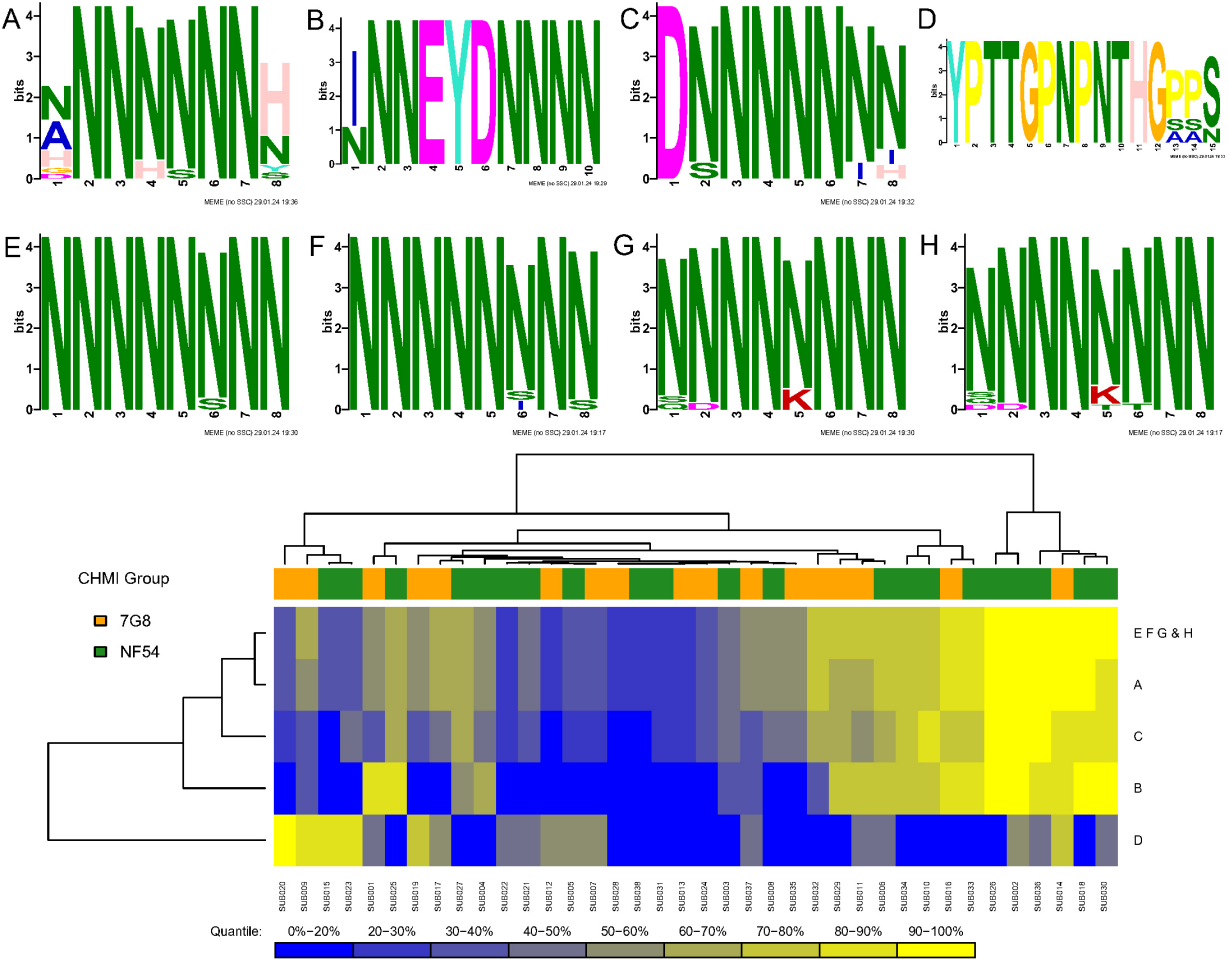
Serologically identified epitopes that overlapped with ABCPred predicted epitopes for highly cross-reactive variants. Serologically identified epitopes were ascertained based on intensity of responses [seroreactivity (MMMF), A and C] and breadth of responses [serorecognition (MMNS), B-H]. **(A)** Shared serologically identified epitope for the NF54 CHMI group based on MMMF that occurs in PF3D7_0305300. **(B)** Shared serologically identified epitope for the NF54 CHMI group based on MMNS occurring in PF3D7_0726400. **(C)** Shared serologically identified epitope for the NF54 CHMI group based on MMNS and the 7G8 CHMI group based on MMMF occurring in PF3D7_0828100. **(D)** Shared serologically identified epitope for the NF54 CHMI group based on MMNS occurring in PF3D7_1033200 **(D)**. **(E, F)** Shared serologically identified epitope for the NF54 CHMI group **(E)** and the 7G8 CHMI group **(F)** based on MMNS occurring in PF3D7_1205500. **(G, H)** Shared serologically identified epitope for the NF54 CHMI group **(G)** and the 7G8 CHMI group **(H)** based on MMNS occurring in PF3D7_1447800. Below the epitopes is a heatmap summarizing the maximum number of serorecognized protein variants (MMNS) for each participant for the epitopes depicted in **(A–H)** (blue-yellow: low-high, in green: NF54, and gold: 7G8). Responses were overall heterogeneous between participants without obvious clustering by Euclidean distances by CHMI group. The asparagine-rich epitopes clustered together and about a third of participants had high breadth of responses (MMNS) versus epitope D, which was not asparagine-rich, for which a distinct set of about 15% of participants had high breadth of responses.

When comparing ABCPred-predicted and serologically identified epitopes with TOMTOM, 65 of the 543 total ABCPred-predicted epitopes from 19 proteins overlapped with serologically identified epitopes from at least one CHMI group based on MNS or MMMF (**Supplementary Table S9**). Six of these 19 proteins with overlapping epitopes had highly cross-reactive protein variants with ≥99% of protein variants serorecognized by at least one CHMI group: PF3D7_0305300, PF3D7_0726400, PF3D7_0828100, PF3D7_1033200, PF3D7_1205500, PF3D7_1447800 (**Table 4**, **Figure 5**). Five were CHMI strain specific: PF3D7_0305300, PF3D7_0726400, PF3D7_0828100, PF3D7_1033200, and PF3D7_1447800, i.e., one CHMI group serorecognized all variants, but the other group serorecognized few or none. One protein, PF3D7_1205500, was highly cross-reactive with 100% of protein variants serorecognized by both CHMI groups.

**Table 4:** Proteins containing ABCPred predicted epitopes that overlapped with serologically identified epitopes from at least one CHMI group.

Protein	Name	Total # of variants on the array	# of protein variants serorecognized by 7G8 CHMI group	# of protein variants serorecognized by NF54 CHMI group	Maximum proportion of variants serorecognized	Lifecycle stage	GO terms
PF3D7_0110600	phosphatidylinositol-4-phosphate 5-kinase	104	3	0	3%	Sporozoite	calcium ion binding, ATP binding, phosphatidylinositol phosphate kinase activity, small GTPase binding
PF3D7_0206800	MSP2	176	12	0	7%	Merozoite	cell adhesion, nucleus, plasma membrane, integral component of plasma membrane, anchored component of plasma membrane
PF3D7_0305300	conserved Plasmodium membrane protein, unknown function	192	5	192	100%	Unavailable	cell surface, membrane, integral component of membrane
PF3D7_0310300	phosphoglycerate mutase, putative	170	101	0	59%	Gametocyte	nucleus, cytoplasm, mRNA binding, intramolecular transferase activity
PF3D7_0420000	Zinc finger protein, putative	201	0	0	0%	Sporozoite, Trophozoite, Gametocyte	nucleus, cytoplasm, membrane, zinc ion binding
PF3D7_0726400	conserved Plasmodium membrane protein, unknown function	200	198	0	99%	Sporozoite, Gametocyte	DNA repair, integral component of membrane, nuclease activity, 5’-flap endonuclease activity, 5’-3’ exodeoxyribonuclease activity, flap endonuclease activity
PF3D7_0731800	alpha/beta hydrolase, putative	128	0	0	0%	Unavailable	Membrane, food vacuole, lipase activity
PF3D7_0828100	conserved Plasmodium protein, unknown function	197	197	0	100%	Unavailable	No data available
PF3D7_0909000	conserved Plasmodium protein, unknown function	128	13	0	10%	Gametocyte	No data available
PF3D7_0930500	diacylglycerol kinase, putative (DGK1)	219	22	0	10%	Sporozoite	Signal transduction, integral component of membrane, kinase activity
PF3D7_1021100	conserved Plasmodium protein, unknown function	239	0	3	1%	Unavailable	No data available
PF3D7_1021700	conserved Plasmodium membrane protein, unknown function	121	3	0	2%	Sporozoite	Integral component of membrane
PF3D7_1033200	early transcribed membrane protein 10.2	50	50	0	100%	Trophozoite	Nucleus, symbiont-containing vacuole membrane, Maurer’s cleft
PF3D7_1205500	zinc finger protein	248	248	248	100%	Merozoite, Gametocyte	Protein ubiquitination, nucleus, cytoplasm, protein binding
PF3D7_1229100	Multidrug resistance-associated protein 2 (MRP2)	274	2	0	1%	Sporozoite	Schizogony, response to drug, xenobiotic transport, transmembrane transport, integral component of plasma membrane, ATP binding
PF3D7_1324600	conserved Plasmodium protein, unknown function	110	96	11	87%	Unavailable	Integral component of membrane
PF3D7_1325900	conserved Plasmodium protein, unknown function	198	137	0	69%	Sporozoite	No data available
PF3D7_1343300	conserved plasmodium protein	99	0	0	0%	Trophozoite, Gametocyte	Nucleus
PF3D7_1447800	calponin homology domain-containing protein	230	230	0	100%	Sporozoite, Gametocyte	Protein binding

## 4 Discussion

Overall, we observed key differences in antibody responses to peptides and protein variants between two groups of malaria-naïve participants infected with either the NF54 or 7G8 strain of *P. falciparum* via CHMI. Because the participants were malaria-naïve and the strain with which they were infected was known, the cross-reactive responses seen were driven solely by the single malaria episode. By contrast, in studies of participants from malaria endemic countries, breadth of responses is driven by both past exposures and cross-reactivity, and is, therefore, more difficult to interpret in the context of identifying responses that could drive the broad protection needed for a highly effective malaria vaccine. Our analysis of peptide-level antibody responses in malaria-naïve participants will be informative to subsequent studies in malaria endemic populations. For 20 of the 227 proteins, one strain group showed greater breadth (cross-reactivity) of antibody responses than the other, with the 7G8 CHMI group having higher serorecognition than the NF54 CHMI group for 17 of these 20 proteins. Aggregate signals across each protein, representing the amount of antibody response, did not differ between CHMI groups. This combination of findings is interesting in the context of the differences in study design. Most (76%) of the NF54 CHMI group samples were obtained from a trial where malaria was diagnosed by thick blood smear (TBS), but all of the 7G8 CHMI group samples were obtained from a trial where malaria was diagnosed by ultrasensitive PCR (usPCR, **Table 1**). Because usPCR becomes positive during malaria infections 1–3 days prior to TBS, and treatment is initiated upon diagnosis by TBS or uPCR, the 7G8 CHMI group participants had presumably shorter exposure to blood stage malaria. The shorter exposure time of the 7G8 CHMI group might therefore be expected to result in lower antibody responses; however, we saw more cross-reactive antibody responses in the 7G8 CHMI group and similar intensity of antibody responses for both CHMI groups. Although it is possible that NF54 has become attenuated over time (44), in a small head to head comparison study of 7G8 and NF54, infectivity was similar for the two strains (36). Even when considering these alternative explanations, our findings suggest that protein variants from some *P. falciparum* strains elicit more cross-reactive antibody responses than others.

We identified protein areas of high antibody binding (either breadth or intensity) and used MEME to identify dominant motifs within these areas. The serologically identified epitopes identified by MEME were highly repetitive and contained many asparagine residues. Repetitive proteins are known to be immunogenic and cross-reactive, but it is uncertain whether high antibody responses to these repeat regions are protective in individuals living in malaria endemic areas (45, 46). Raghavan et al. showed enrichment of repetitive elements in antibody targets when examining serum from children and adults in high and moderate transmission settings, and higher breadth of responses to repetitive peptides in children in high transmission settings compared to moderate transmission settings (47). The moderate and high transmission settings differed both in number of exposures and time between exposure and sample collection, so the authors postulated that the responses to repetitive peptides could be exposure-dependent and/or short-lived. The Raghavan et al. study used the phage-immunoprecipitation sequencing (PhIP-Seq) technology, which results in longer peptides (up to 90 amino acids in length) and can allow for some capture of conformational epitopes but loses the resolution of epitope mapping possible with peptide arrays (47, 48). Additionally, PhIP-Seq uses serial enrichment and amplification, which can result in potential amplification of non-specific phages and decreased specificity (48); whereas the peptide arrays used in the current study are fabricated onto slides such that each antibody-bound peptide has a known sequence. Our study shows that antibodies against repetitive motifs can develop after a single exposure. Follow-up testing in CHMI studies could further elucidate the longevity of responses.

A serologically identified epitope in our study, EPNQANKE, matched two literature-curated epitopes in merozoite surface protein-2 (MSP-2, PF3D7_0206800), EPNQANKE and ECTDGNKE. Prior studies of the association between anti-MSP-2 antibodies and protection from clinical malaria have been conflicting, perhaps due to the use of different methods, including assessment of different IgG subclasses and antigens (49–54). While EPNQANKE lies within the variable region of MSP-2, ECTDGNKE is in the conserved C-terminal region (55). A study of a mouse monoclonal antibody against the conserved C-terminal region of MSP-2 identified a minimal binding epitope (NKENCGAA) that shares the asparagine-lysine-glutamate residues present in both epitopes, evoking the idea that a single cross-reactive antibody could bind both epitopes identified in the current study (56). The paradigm of cross-reactive epitopes within the same protein is exemplified by CSP. CIS43 and L9, two CSP monoclonal antibodies in clinical development for primary malaria prophylaxis, display cross reactivity with the major (NANP) repetitive motif of the central repeat region (immunodominant) and their primary target (immunoprotective) (14, 57). Further work isolating and characterizing monoclonal antibodies targeting MSP-2 could determine whether similar cross-reactivity could be important for protective responses.

Serologically identified epitopes from one or both CHMI groups overlapped with epitopes predicted by ABCPred in 19 proteins. Breadth of responses were high for six of these proteins, PF3D7_0305300 (transporter, putative); PF3D7_0726400 (conserved Plasmodium membrane protein, unknown function); PF3D7_0828100 (conserved Plasmodium protein, unknown function); PF3D7_1033200 (early transcribed membrane protein 10.2, ETRAMP10.2); PF3D7_1205500 (zinc finger protein, putative); and PF3D7_1447800 (calponin homology domain-containing protein, putative), with ≥99% of protein variants serorecognized by one or both CHMI groups. The 7G8 CHMI group had more cross-reactive responses, serorecognizing ≥99% of the variants of four of the six proteins compared to the NF54 CHMI group, which serorecognized none or very few. This result shows that strain choice in the design of malaria vaccines could have significant impact on cross strain protection. Many of the serologically identified epitopes that overlapped with ABCPred predicted epitopes were asparagine rich, so may not be good vaccine candidates (47). But a cross-reactive epitope from PF3D7_1033200 (ETRAMP10.2, YPTTGPNPNTHGPPS) was not asparagine-rich and was unique to this protein. ETRAMP10.2 is expressed in the parasitophorous vacuole membrane during the blood stage and may be involved in red blood cell binding (58, 59). The 7G8 CHMI group serorecognized all 50 variants of ETRAMP10.2 on the array. A portion of the ETRAMP10.2 epitope (NPNTHGPP) was a dominant motif identified by MEME based on serorecognition for the NF54 CHMI group. However, NF54 group antibody responses to the whole ETRAMP10.2 protein were below the serorecognition threshold, which suggests that examining whole protein serorecognition to define antigenicity can be less sensitive than studying individual epitopes. This may have implications for which variant is the best choice should ETRAMP10.2 become a vaccine candidate.

These findings highlight the importance of considering strain-specific responses when designing novel malaria vaccine targets. For several proteins on the array, antibodies elicited by the 7G8 CHMI were more cross-reactive with the non-CHMI strain protein variants that than antibodies elicited by the NF54 CHMI, highlighting that the strain used for CHMI had a notable impact on responses. Because of the size and complexity of the *P. falciparum* genome, it is not possible with current technologies to study humoral responses to all *P. falciparum* proteins encoded in the genome with comprehensive representation of variants with one assay. Our assay was designed to study a broad range of proteins from different lifecycle stages that were predominantly known surface-exposed proteins. However, we recognize that our results are limited to the proteins selected and we likely missed additional proteins with differential responses between the two groups due to the design of the peptide array. Our results are limited to serological responses and, therefore, do not characterize the contributions of cell mediated immunity. While we recognize that serological responses are only a part of the complex human immune response to malaria, antibody responses have been shown to play an important role in protection from symptomatic malaria and in studies of RTS,S- and R21-induced protection (8, 9, 22, 60–64). Our participants were malaria-naïve adults at the time of CHMI, as is the standard, so findings may not be generalizable to endemic countries where first malaria exposures occur early in childhood. But, to our knowledge, this is the first study to compare CHMI with the NF54 and 7G8 strains to identify differences in humoral responses to a vast array of diverse proteins. Because these findings describe responses that develop after a single malaria episode and do not provide information on future protection from malaria, further work is needed to determine their clinical relevance. However, we were able to discriminate important epitopes with overlap in known and predicted epitopes. Our approach has the potential to find important strain-specific and cross-reactive responses highly relevant to malaria vaccine development.

## Data Availability

The datasets generated for this study can be found in the Figshare repository, https://doi.org/10.6084/m9.figshare.28278923.
