# Supplementary material for: Controlled human malaria infection with NF54 and 7G8 strains elicit differential antibody responses to Plasmodium falciparum peptides
Source: Front Immunol. 2025 Sep 15;16:1641280. doi: 10.3389/fimmu.2025.1641280 (PMC12477171; doi:10.3389/fimmu.2025.1641280)
Supplement: Supplementary file 1 [file DataSheet1.pdf]

# Supplementary Material

## 1 Supplementary Methods

### 1.1 Peptide Lab Methods

#### 1.1.1 Array Synthesis

The *in situ* peptide synthesis, quality control, serum probing and slide imaging procedures are similar to those have been detailed previously (1). Microarrays were synthesized by light-directed solid-phase peptide synthesis with a Nimble Therapeutics Maskless Array Synthesizer (MAS) using an amino-functionalized support (Greiner Bio-One) coupled with a 6-aminohexanoic acid linker and amino acid derivatives carrying a photosensitive 2-(2-nitrophenyl) propyloxycarbonyl (NPPOC) protection group (Orgentis Chemicals). Amino acids were pre-mixed for ten minutes to a final concentration of 20 mM in *N,N*-Dimethylformamide (DMF, Sigma Aldrich) with *N,N,N',N'*-Tetramethyl-O-(1H-benzotriazol-1-yl)uronium-hexafluorophosphate (HBTU, Protein Technologies, Inc.; final concentration 20 mM) as an activator, 6-Chloro-1-hydroxybenzotriazole (6-Cl-HOBt, Protein Technologies, Inc.; final concentration 20 mM) to suppress racemization, and *N,N*-Diisopropylethylamine (DIPEA, Sigma Aldrich; final concentration 31 mM) as base. Activated amino acids were then coupled to the array surface for three minutes. The microarray was then washed with *N*-methyl-2-pyrrolidone (NMP, VWR International), and site-specific cleavage of the NPPOC protection group was accomplished by irradiation of an image created by a Digital Micro-Mirror Device (Texas Instruments), projecting 365 nm wavelength light. These coupling cycles were repeated as necessary to synthesize the full peptide library. All arrays passed proprietary QC metrics including amino acid quality control, synthesis drift, background signal, and signal uniformity.

#### 1.1.2 Sample Binding and Detection

Samples were divided into nine batches in such a way that a both samples (baseline and pre-CHMI) for one participant were in the same batch, but that study arms and protection status were balanced across batches.

Before sample binding, arrays were treated with 95% trifluoroacetic acid (TFA, Sigma Aldrich) and 0.5% Triispropylsilane (TIPS, TCI Chemicals) for 30 minutes to remove any side chain protecting groups. Arrays were then incubated two times in methanol for 30 seconds, rinsed four times with reagent-grade water (Ricca Chemical Co.), washed for one minute in TBST (1× TBS, 0.05% Tween-20), washed two times for one minute in TBS, and washed for 30 seconds in reagent-grade water as a final wash.

Samples were diluted 1:100 in binding buffer (0.01M Tris-Cl, pH 7.4, 1% alkali-soluble casein, 0.05% Tween-20) and bound to arrays overnight at 4°C. The arrays were then washed three times for ten minutes each time in wash buffer (1× TBS, 0.05% Tween-20). Primary sample binding was detected via Alexa Fluor® 647-conjugated goat anti-human IgG secondary antibody and Cy3-conjugated goat anti-human IgA secondary antibody (Jackson ImmunoResearch) diluted 1:10,000 (final concentration 0.1 ng/µl) in secondary binding buffer (1x TBS, 1% alkali-soluble casein, 0.05% Tween-20). After incubation with secondary antibody for three hours at room temperature, arrays were washed three times (ten minutes per wash) in wash buffer, washed 30 seconds in reagent-grade water, and dried by spinning in a microcentrifuge equipped with an array holder. Fluorescent signal

of the secondary antibody was captured using an InnoScan 1100AL microarray scanner (Innopsys Inc.) by scanning at 635 nm at 2 μm resolution. Proprietary Nimble Therapeutics software was used to extract fluorescence intensity values for each peptide from scanned array images.

## 1.2 Data Normalization

Other methods of normalization and correction were visualized to find the best fit for the data and minimize potential bias. Data from Nimble Therapeutics included both raw data and spatial and background corrected data (2). Spatial correction was conducted using 2-dimensional loess smoothing and background correction was performed using deconvolution. Median normalization of study data using blank spots on the array was executed using intensities collected for the blank spots included on the arrays. Sample specific medians were calculated for the fluorescence intensities corresponding to the blank spots. The median of all sample specific medians, termed the global median, was then calculated. A scaling factor was then calculated for each sample, which was the difference between the sample specific median global median, to center the sample specific median of the blank spots to the global blank spot median FI. The full data set then was normalized by subtracting the sample-specific scaling factor from each of the individual peptide $\log_2$ intensities.

# 2 Supplementary Figures

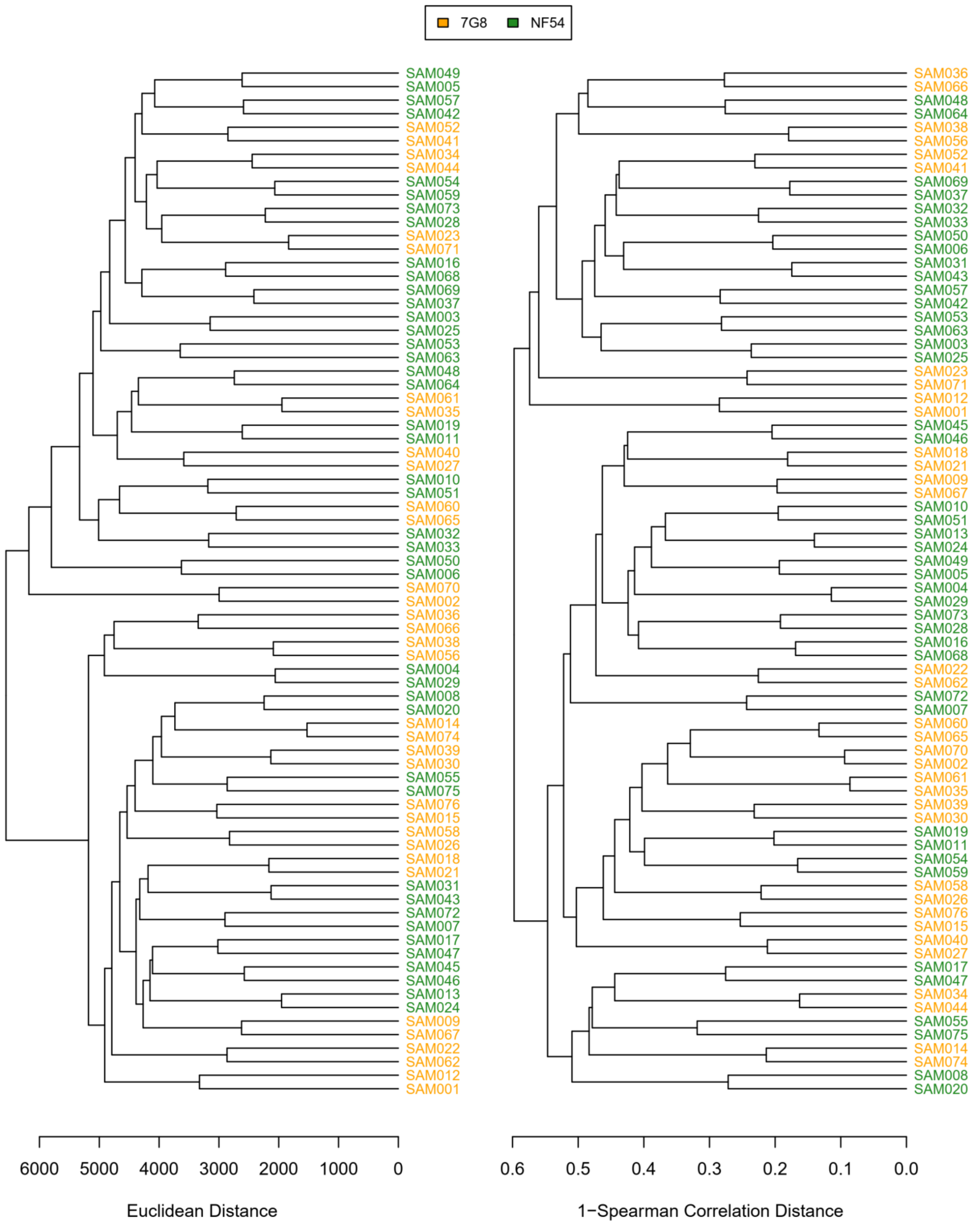


**Supplementary Figure 1.** Hierarchical clustering plots (not normalized, standardized variables). Euclidean distances were hierarchically clustered using the complete linkage clustering algorithm.

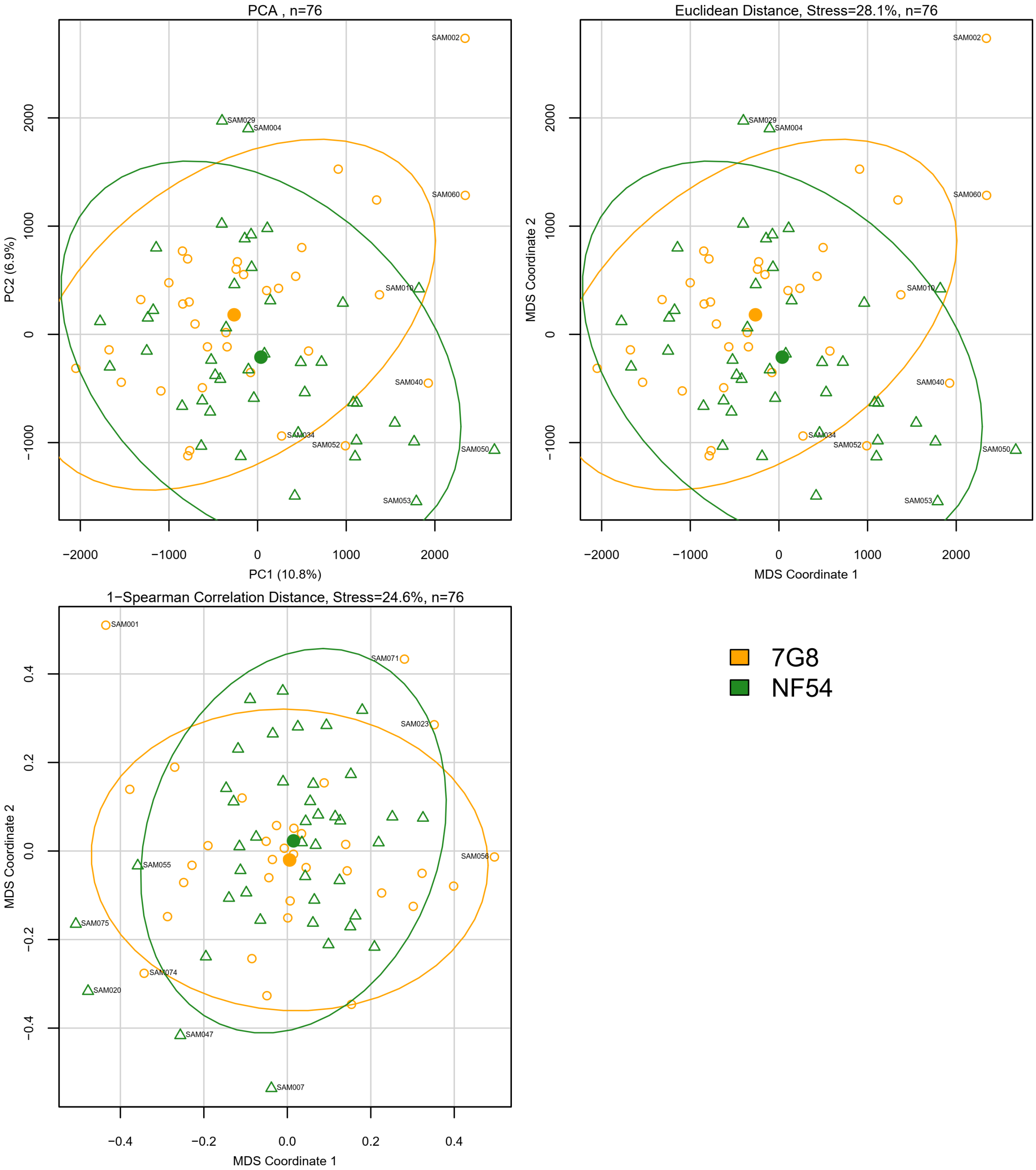


**Supplementary Figure 2.** PCA and non-metric multidimensional scaling biplots (Not Normalized, Standardized Variables) by CHMI group. PCA biplots with bivariate 95% confidence ellipses are shown at the top left. Percent explained variance is printed after each axis label. Non-metric MDS results with bivariate 95% confidence ellipses based on Euclidean distance are shown in the top right. Non-metric MDS results with bivariate 95%confidence ellipses based on 1-Spearman correlation distance are shown at the bottom left. Labels for the five most outlying samples per cohort based on maximum Mahalanobis distance are shown.

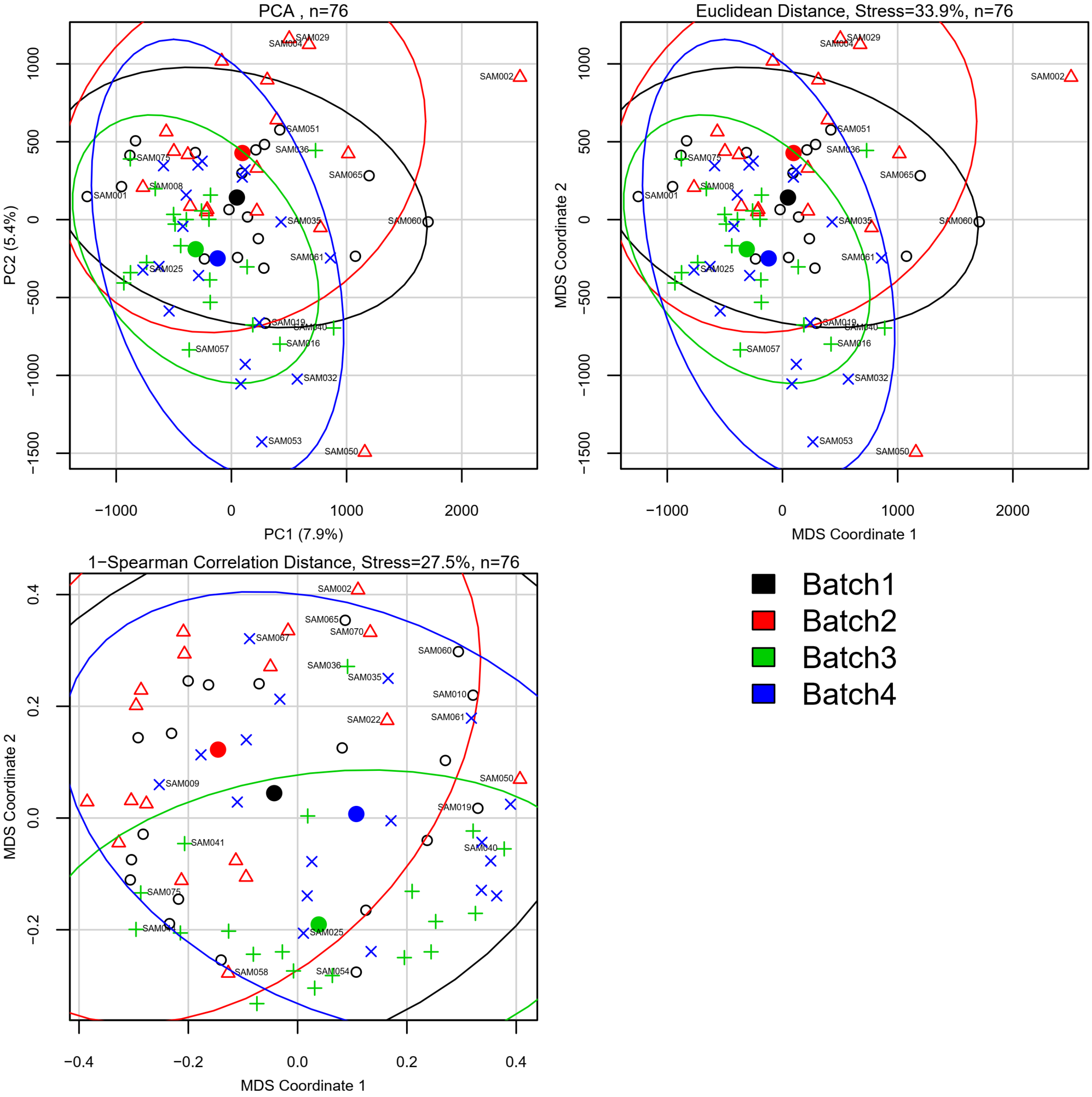


**Supplementary Figure 3.** PCA and non-metric multidimensional scaling biplots (Not Normalized, Standardized Variables) by batch run. PCA biplots with bivariate 95% confidence ellipses are shown at the top left. Percent explained variance is printed after each axis label. Non-metric MDS results with bivariate 95% confidence ellipses based on Euclidean distance are shown in the top right. Non-metric MDS results with bivariate 95% confidence ellipses based on 1-Spearman correlation distance are shown at the bottom left. Labels for the five most outlying samples per cohort based on maximum Mahalanobis distance are shown.

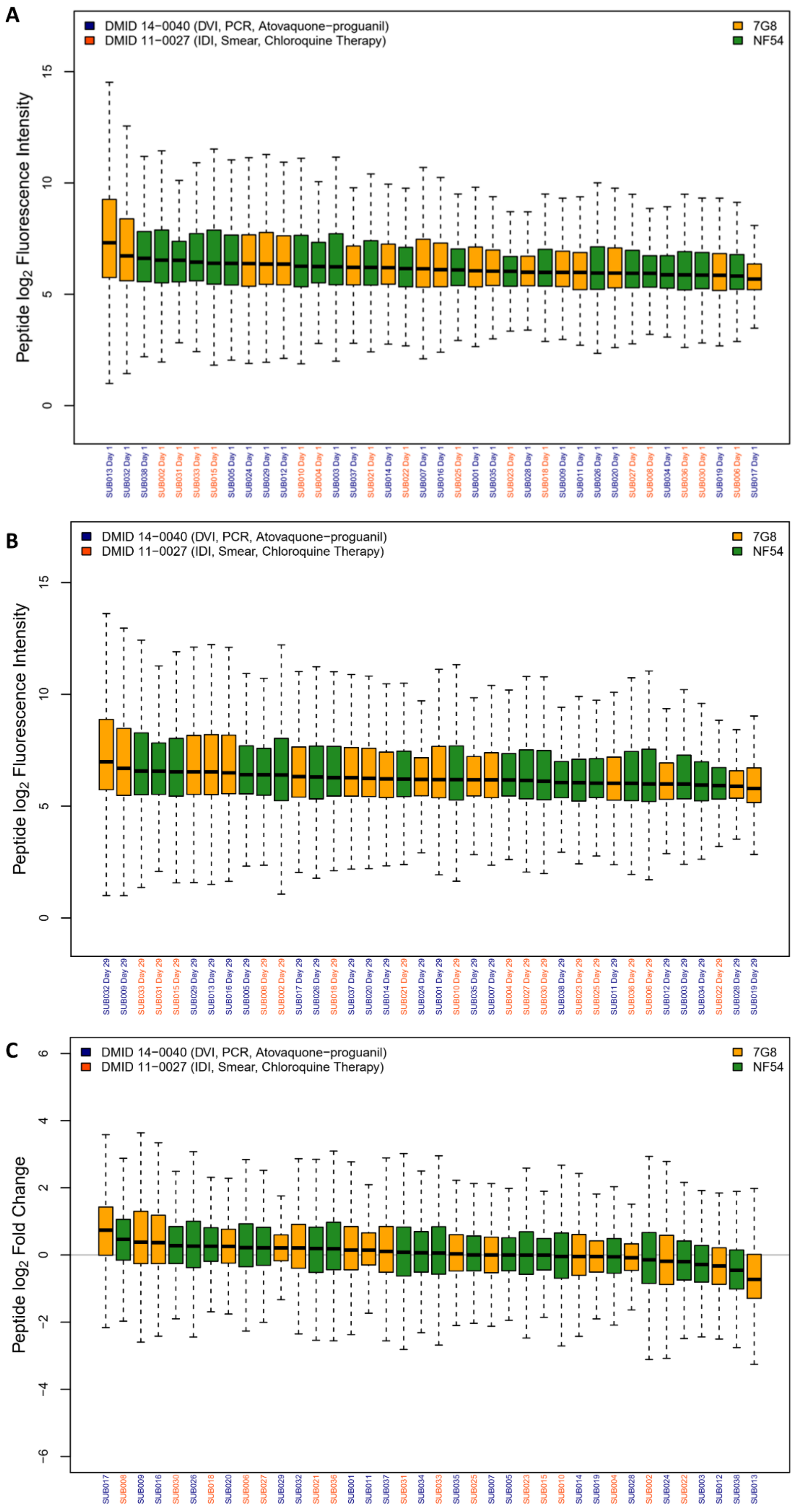
A
B
C
DMID 14−0040 (DVI, PCR, Atovaquone−proguanil)
DMID 11−0027 (IDI, Smear, Chloroquine Therapy)
7G8
NF54
Peptide log2 Fluorescence Intensity
Peptide log2 Fold Change

**Supplementary Figure 4.** Box plots of peptide $log_2$ fluorescence intensities for each subject at (A) baseline (Day 1), (B) post-controlled human malaria infection (CHMI, Day 29), and (C) fold change (Day 29 vs. Day 1). Samples are ordered by median response. Sample labels colored in blue were from the dose ranging study of CHMI given by direct venous inoculation (DVI), diagnosed by PCR, and treated with atovaquone-proguanil. Sample labels colored in orange were from a study giving NF54 by intradermal or subcutaneous inoculation diagnosed by blood smear and treated with chloroquine.

## 3 References

1. Friedman-Klabanoff DJ, Travassos MA, Ifeonu OO, Agrawal S, Ouattara A, Pike A, et al. Epitope-specific antibody responses to a Plasmodium falciparum subunit vaccine target in a malaria-endemic population. J Infect Dis (2020). doi: 10.1093/infdis/jiaa611

2. Lo KC, Sullivan E, Bannen RM, Jin H, Rowe M, Li H, et al. Comprehensive Profiling of the Rheumatoid Arthritis Antibody Repertoire. Arthritis Rheumatol (2020) 72:242-50. doi: 10.1002/art.41089
