# Supplementary material for: Controlled human malaria infection with NF54 and 7G8 strains elicit differential antibody responses to Plasmodium falciparum peptides
Source: Front Immunol. 2025 Sep 15;16:1641280. doi: 10.3389/fimmu.2025.1641280 (PMC12477171; doi:10.3389/fimmu.2025.1641280)
Supplement: Supplementary file 2 [file Table1.pdf]

Supplementary Table S1: Demographics of Study Participants by CHMI Group

		7G8 CHMI Group (N = 17)	NF54 CHMI Group (N=21)
**Variable**	**Characteristic**	**n (%)**	**n (%)**
Sex	Male	6 (35)	15 (71)
	Female	11 (65)	6 (29)
Ethnicity	Not Hispanic or Latino	15 (88)	19 (91)
	Hispanic or Latino	2 (12)	2 (9)
Race	Asian	1 (6)	0 (0)
	Black or African American	9 (53)	16 (76)
	White	7 (41)	6 (29)
	American Indian/ Alaskan Native	0 (0)	1 (5)
Age	Years	31.7 (6.8)	34.9 (7.2)
Study	ID group (1)	17 (100)	16 (76)
	DVI group (2)	0 (0)	5 (24)

1. Lyke KE, Laurens MB, Strauss K, Adams M, Billingsley PF, James E, et al. Optimizing Intradermal Administration of Cryopreserved Plasmodium falciparum Sporozoites in Controlled Human Malaria Infection. Am J Trop Med Hyg. 2015;93(6):1274-84. doi: 10.4269/ajtmh.15-0341

2. Laurens MB, Berry AA, Travassos MA, Strauss K, Adams M, Shrestha B, et al. Dose-Dependent Infectivity of Aseptic, Purified, Cryopreserved Plasmodium falciparum 7G8 Sporozoites in Malaria-Naive Adults. J Infect Dis. 2019;220(12):1962-6. doi: 10.1093/infdis/jiz410
