# Supplementary material for: Controlled human malaria infection with NF54 and 7G8 strains elicit differential antibody responses to Plasmodium falciparum peptides
Source: Front Immunol. 2025 Sep 15;16:1641280. doi: 10.3389/fimmu.2025.1641280 (PMC12477171; doi:10.3389/fimmu.2025.1641280)
Supplement: Supplementary file 5 [file Table4.pdf]

Supplemental Table S4: List of *P. falciparum* B-cell predicted protein epitopes identified for all proteins. Predicted epitopes were filtered to retain only those epitopes with a score greater than or equal to 0.9. The higher the score, the higher the likelihood of a true hit. Proteins in this table are limited to experiment 2 diversity array proteins.

Protein ID	Rank	Sequence	Start Position	Score
PF3D7_0102200	1	DGVYLDHETSDALYTD	396	0.96
PF3D7_0102200	2	LGDIDKKRWYNKYGYD	574	0.95
PF3D7_0102200	3	MHGISTINKEMKNQNE	867	0.93
PF3D7_0102200	4	EHDAEENVEHDAEENV	897	0.92
PF3D7_0102200	4	DEHVEEPTVADEHVEE	467	0.92
PF3D7_0102200	4	FGLPFEKPTFTLESPP	111	0.92
PF3D7_0102200	4	FSQQYMDTKNVKEKNP	11	0.92
PF3D7_0102200	5	QHNAEANVEHDAEENV	889	0.91
PF3D7_0102200	6	VEEVEENVEENVEENV	987	0.90
PF3D7_0102200	6	LSSIMERYAGGKRNDK	837	0.90
PF3D7_0102200	6	VEEVEENVEENVEENV	1037	0.90
PF3D7_0102200	6	VEEVEENVEENVEENV	1014	0.90
PF3D7_0103800	1	TIRISAPPERKFSTFI	331	0.95
PF3D7_0103800	2	IVTSITRADMDLRKTL	283	0.94
PF3D7_0103800	3	KKIWISKQEFDEYGSV	358	0.90
PF3D7_0106300	1	GNKINTTSTHNNNNNN	651	0.97
PF3D7_0106300	2	TALGFNPPEHDVMKCK	1017	0.96
PF3D7_0106300	3	CYSEYDYNFYMCLVNC	503	0.91
PF3D7_0106300	3	KKMKNDLNNNNNNNNN	450	0.91
PF3D7_0106300	3	EGSIDEADPYSDYFSS	432	0.91
PF3D7_0106300	4	KIEICDFIEPLVIVLI	88	0.90
PF3D7_0106300	4	YEKNTTPVQSSNKKDK	566	0.90
PF3D7_0109100	1	GATIDINDCHNNTLNN	729	0.96
PF3D7_0109100	2	KFLIDYGDVFNNYGSF	873	0.95
PF3D7_0109100	2	IIEIENKGNSNNNNNI	513	0.95
PF3D7_0109100	3	KGSRDWDYCRDSINYD	85	0.93
PF3D7_0109100	3	LKTQEHPTYNNFSLSI	464	0.93
PF3D7_0109100	3	QQIIQSHYFFTENIIP	404	0.93
PF3D7_0109100	3	EEVVDMTDENMYHYNQ	302	0.93
PF3D7_0109100	4	TENIIPPTRFSSLDPD	414	0.92
PF3D7_0109100	4	TFTIETDCYIRLLVNN	278	0.92
PF3D7_0109100	5	EFRQQHRKTLDGRLCA	33	0.90
PF3D7_0109100	5	DQLQIDIKEENKNVKQ	185	0.90
PF3D7_0114000	1	FISIITNYYPFKEQNN	1338	0.96
PF3D7_0114000	2	EGEEDDDDDEDDEDDE	1035	0.94
PF3D7_0114000	3	GNIWNDTIYDDNYNNN	1068	0.93
PF3D7_0114000	4	SEDIEDYEELGKEEEP	840	0.92
PF3D7_0114000	5	EEEGEKEESEDKGDKE	492	0.91
PF3D7_0114000	5	EEEGEKEESEDKGDKE	372	0.91
PF3D7_0114000	5	GEDITSFLENKNKIIQ	1018	0.91
PF3D7_0114000	6	PEIGTDIEEENKEKYI	986	0.90
PF3D7_0114000	6	SEDIEDSEEVGKEEEP	800	0.90
PF3D7_0114000	6	DEVEKEETSEDVRDNE	642	0.90
PF3D7_0114000	6	HSMKERRYYSIYKKDN	2	0.90
PF3D7_0206900	1	GQEPPNRLHFDNVDDE	162	0.94
PF3D7_0206900	2	KEEGSLPTKMNEKNSN	63	0.92
PF3D7_0207000	1	HEEDDDDEDDDTYNKD	184	0.91
PF3D7_0207000	2	EGEGEHEEEEEHEEDD	173	0.90
PF3D7_0207000	2	HFFIICTINFDKLYIS	11	0.90
PF3D7_0207100	1	SSTCEKKNKEDNKMND	677	0.95

Protein ID	Rank	Sequence	Start Position	Score
PF3D7_0207100	1	IVPIIKCSNYKIKNNP	119	0.95
PF3D7_0207100	2	DKGIDLHNKMMKIETD	302	0.94
PF3D7_0207100	2	HIFSYAYEDMDKIKKK	1717	0.94
PF3D7_0207100	3	VNIQNEGNGFNNNKNN	644	0.93
PF3D7_0207100	4	YVTLKNKVHVNKYVNN	96	0.92
PF3D7_0207100	4	KKKHDEKYYNNIFLNP	326	0.92
PF3D7_0207100	4	TKTNISYNKMNSFKDN	200	0.92
PF3D7_0207100	5	KRTLKGDGHDDVKTNM	786	0.91
PF3D7_0207100	5	KKKIKKKNKMKNKSNN	585	0.91
PF3D7_0207100	6	EKSQGTNLFYDNYINS	926	0.90
PF3D7_0207100	6	DQFYDSYNMRNEKITD	755	0.90
PF3D7_0207100	6	SKKISNNNNNDNMNNQ	610	0.90
PF3D7_0207100	6	LERVDEPRLYNNVDKI	39	0.90
PF3D7_0207100	6	KGRVDYCIIPNEDDKN	1065	0.90
PF3D7_0209000	1	PKTVIGIKCPNKKLNP	2712	0.97
PF3D7_0209000	1	YIDIKEPFYFMFGCNN	1101	0.97
PF3D7_0209000	2	PGAIVSPMKVLKKKDP	1843	0.96
PF3D7_0209000	2	PIYIKDIYEFNIVCDN	1395	0.96
PF3D7_0209000	3	SEHINYYIHKDKKYNL	2270	0.95
PF3D7_0209000	4	HKDIHFSLEFNNSLSL	2942	0.94
PF3D7_0209000	5	GGESFTYEKSEVDKTD	466	0.93
PF3D7_0209000	5	EEEEYDDYVYEESGDE	301	0.93
PF3D7_0209000	5	AISINNPDEKDNTYTY	2767	0.93
PF3D7_0209000	5	KFSLKPSLVFDDNNND	2107	0.93
PF3D7_0209000	5	TIIIKKNVTNDHVVSS	129	0.93
PF3D7_0209000	6	SGDIGGILFPKNIKST	787	0.92
PF3D7_0209000	6	YGLLISPTVNEKENNF	668	0.92
PF3D7_0209000	6	LKYIYMYLTPSDSFNL	242	0.92
PF3D7_0209000	6	PPIVPKDLHFKVECNN	1868	0.92
PF3D7_0209000	7	NKEHDYTCDFTDKLDK	915	0.91
PF3D7_0209000	7	QIEEDEHNEKIKMKTF	752	0.91
PF3D7_0209000	7	TESGDTAVSEDSYDKY	567	0.91
PF3D7_0209000	7	GEEEGEYVDEKERQGE	438	0.91
PF3D7_0209000	7	KEDITFHLFCGKSTTK	2629	0.91
PF3D7_0209000	7	NEHICDYEKNESLIST	2448	0.91
PF3D7_0209000	7	HIDISNSNRKINGCDF	1901	0.91
PF3D7_0209000	7	EEKIQRDTYENKDYES	173	0.91
PF3D7_0209000	8	YGEYGEAVEDGENVIK	523	0.90
PF3D7_0209000	8	GKDICKYDVTTKVATC	2831	0.90
PF3D7_0209000	8	KIKIDEKKTIGKDICK	2821	0.90
PF3D7_0209000	8	IHMKIEKSTMDKIKID	2810	0.90
PF3D7_0209000	8	TVKIKKGDIFGLKCPK	2230	0.90
PF3D7_0209000	8	TSHTNTIGTMKVTLNK	2019	0.90
PF3D7_0209000	8	DTLIEWFDDNTNEENF	190	0.90
PF3D7_0217100	1	AIAIDAIIHQKNINDN	216	0.91
PF3D7_0217100	2	SELVEIHNEDDKGSVT	83	0.90
PF3D7_0220000	1	HYLQQMKEKFSKEKNN	1500	0.95
PF3D7_0220000	2	DEDIDEDIEEDVEEDI	1305	0.94
PF3D7_0220000	3	LGGIETEEIKDSILNE	600	0.93
PF3D7_0220000	3	DEDIDEDIDEDIGEDK	1329	0.93
PF3D7_0220000	4	KGSILDMLKGDMELGD	1202	0.92
PF3D7_0220000	4	VEHIISGDAHIKGLEE	1177	0.92
PF3D7_0220000	5	TVSIIEEMEENIVDVL	914	0.91

Protein ID	Rank	Sequence	Start Position	Score
PF3D7_0220000	5	NKSWKKNTYVDKKLNK	70	0.91
PF3D7_0220000	6	TESIKDKEKDVSLVVE	958	0.90
PF3D7_0220000	6	EESVEENVEENVEEND	236	0.90
PF3D7_0301700	1	DHTKYHPEYYDEVQEN	92	0.95
PF3D7_0301700	2	YDEVQENYDPYYGVNQ	101	0.93
PF3D7_0301700	3	KMHYDYKHKENNNNNN	268	0.92
PF3D7_0301700	4	LSYIRNGNDNNMQLLP	178	0.91
PF3D7_0303700	1	SGTFATPIVFDNQACI	364	0.94
PF3D7_0303700	2	PGVKRKAKEYKVNLNK	153	0.92
PF3D7_0303700	3	QACIIGIGKMEKKLLL	376	0.90
PF3D7_0303700	3	YTMYKNHNISIAVDTP	295	0.90
PF3D7_0304600	1	KLKQPADGNPDPNANP	93	0.94
PF3D7_0304600	2	GQGHNMPNDPNRNVDE	280	0.92
PF3D7_0304600	3	NVDPNANPNVDPNANP	117	0.91
PF3D7_0304600	3	NVDPNANPNVDPNANP	109	0.91
PF3D7_0304600	4	NPDPNANPNVDPNANP	101	0.90
PF3D7_0305300	1	YLTERSNLEYMEYVNE	712	0.91
PF3D7_0305300	1	NDSIEDMNEDLSYNNN	644	0.91
PF3D7_0305300	1	NHSISHSNNHSNNHNN	211	0.91
PF3D7_0305300	1	DKELYDMSYENNLNNE	144	0.91
PF3D7_0305300	2	QIMVDEQDNNNNYDNN	58	0.90
PF3D7_0305300	2	YEDNEPYNDYNDNNNN	114	0.90
PF3D7_0306400	1	TSAIDNYVPGQIGATV	228	0.95
PF3D7_0306400	2	RKMAQDTLDYILRKYP	474	0.94
PF3D7_0306400	3	AEIIYATRYEFANTIS	579	0.92
PF3D7_0306400	3	YSPCIKRNEMIKKLQE	40	0.92
PF3D7_0306400	4	AGLALDAATRGLKCAL	70	0.91
PF3D7_0306400	5	DLLADLVCYFDKGVPN	169	0.90
PF3D7_0306500	1	YHTIMKRRIIRNVLNI	40	0.94
PF3D7_0306500	1	KPSWQPFWENDCEVIP	118	0.94
PF3D7_0306500	2	DCEVIPRDEFGVPATI	128	0.93
PF3D7_0307700	1	KVDIKRGDENDYIYNN	714	0.96
PF3D7_0307700	2	KDIEQYSNEFKQNNNQ	1674	0.94
PF3D7_0307700	2	LTEEQMGPMFNNHDNE	1590	0.94
PF3D7_0307700	3	ESGEEEYRMMQKKKNT	836	0.93
PF3D7_0307700	3	YYREEDYPEYYNDVPS	572	0.93
PF3D7_0307700	4	YEMVEHKNKYTSNMNN	964	0.92
PF3D7_0307700	4	YEDEEEMNRNNKKNTR	1631	0.92
PF3D7_0307700	4	SSSYQATNTNIQNMNL	1368	0.92
PF3D7_0307700	4	GRAYDKNYYHDNELND	1043	0.92
PF3D7_0307700	4	PYGIEEGRAYDKNYYH	1037	0.92
PF3D7_0307700	5	LNEYNTIDDPDNLNNN	869	0.91
PF3D7_0307700	5	YSNIDSDIYYREEDYP	564	0.91
PF3D7_0307700	5	DDTLKKKEDYNNDNNN	52	0.91
PF3D7_0307700	5	RSMINEKNFQRNEDDH	309	0.91
PF3D7_0307700	5	PMFNNHDNEQGNNLDP	1597	0.91
PF3D7_0307700	5	LNTIINPPFNNQLANA	1402	0.91
PF3D7_0307700	5	IPSIDEKREENSRNFI	1157	0.91
PF3D7_0307700	5	HHLNYNDNYMNTKVNI	1059	0.91
PF3D7_0307700	6	VYLDSYPYEMVEHKNK	957	0.90
PF3D7_0307700	6	NDYKDYYNKGNAVNNY	684	0.90
PF3D7_0307700	6	DKKYDHGDGHHHKYDK	441	0.90
PF3D7_0307700	6	DKKYDHGDGHHHKYDK	427	0.90

Protein ID	Rank	Sequence	Start Position	Score
PF3D7_0307700	6	DKKYDHGDGHHHKYDK	413	0.90
PF3D7_0307700	6	DKKYDHGDGHHHKYDK	399	0.90
PF3D7_0307700	6	DKKYDHDDGHHHKYDK	357	0.90
PF3D7_0310200	1	HVYCYKTYCDDFNLNN	263	0.97
PF3D7_0310200	2	YCHQEDRDDNMNNNDN	1916	0.96
PF3D7_0310200	3	YSSVSSSYEYDDINNV	1616	0.94
PF3D7_0310200	3	QDDIYYDNDFSSICSL	1563	0.94
PF3D7_0310200	4	DYLYDYYKECNNNINN	46	0.93
PF3D7_0310200	4	DKCGDDKCGDDKCGDN	4349	0.93
PF3D7_0310200	4	RKYIKYNTKGNTKGNT	431	0.93
PF3D7_0310200	4	NQTIKEKGGDIKKKDD	3751	0.93
PF3D7_0310200	4	KMSECDMLLQNKRDND	2082	0.93
PF3D7_0310200	5	DKCGDDKCGDDKCGDD	4334	0.92
PF3D7_0310200	5	DKCGDDKCGDDKCGDD	4324	0.92
PF3D7_0310200	5	DKCGDDKCGDDKCGDD	4314	0.92
PF3D7_0310200	5	KKKKEDDNNNNNNNNN	4020	0.92
PF3D7_0310200	5	GKLKGNSTHNNNNRNN	2963	0.92
PF3D7_0310200	5	VQENELSCYPNKNVNV	2674	0.92
PF3D7_0310200	5	KKRDMSNKKGDKSCNK	1756	0.92
PF3D7_0310200	5	NEEITHEQKNNFNKNK	1304	0.92
PF3D7_0310200	6	DDIYDDDDDFNFDHDS	834	0.91
PF3D7_0310200	6	YDDGDDKYDDDDNKYD	816	0.91
PF3D7_0310200	6	TDKINTNDVNVKRENV	3521	0.91
PF3D7_0310200	6	HNMSDHNKEPKKKKNK	1990	0.91
PF3D7_0310200	6	KINIKTDQVYNPLVDS	1468	0.91
PF3D7_0310200	6	RKSINDKEMNDILNNK	1325	0.91
PF3D7_0310200	7	CFSHIDIKTYCNDHTP	4496	0.90
PF3D7_0310200	7	YCCICKKSGGALKKTT	4225	0.90
PF3D7_0310200	7	NNIIIKNDNMNEENNN	4159	0.90
PF3D7_0310200	7	YSFAYPYNYNMNKQND	347	0.90
PF3D7_0310200	7	HVHTNDVKDIDKKKNK	3410	0.90
PF3D7_0310200	7	TQEIQTIIHTNKNSTI	3365	0.90
PF3D7_0310200	7	VQTIDAPNRKPIIDDC	2643	0.90
PF3D7_0310200	7	DEHKKEYNERNDKMSE	2070	0.90
PF3D7_0310200	7	DQLINEDNKKRKINNL	1968	0.90
PF3D7_0310200	7	GDKISKNYHSHNYDNE	1290	0.90
PF3D7_0310200	7	DGKKNNGDDKKKGDNI	1076	0.90
PF3D7_0310200	7	NKYEESANDSDGKKNN	1066	0.90
PF3D7_0310300	1	EKDISSDNKNKNKNND	926	0.94
PF3D7_0310300	1	IEEIFTSDYSSPNLDI	908	0.94
PF3D7_0310300	1	NVLIYDGSEYLIYVPE	723	0.94
PF3D7_0310300	2	IKEEEEEEEYDNDDDF	516	0.93
PF3D7_0310300	2	CSASQNDNSFENKGNK	466	0.93
PF3D7_0310300	2	KGIGETLNTYSCDINP	1093	0.93
PF3D7_0310300	3	EYLIYVPEKGSWENNK	731	0.92
PF3D7_0310300	3	EEEYDNDDDFTVENNK	522	0.92
PF3D7_0310300	3	YDEEYTYNKSSNKKNL	46	0.92
PF3D7_0310300	3	QKLIKLYNESVHGKDE	221	0.92
PF3D7_0310300	4	EEEYVSLNTKNKEQNE	775	0.91
PF3D7_0310300	4	NGLYKNYVLRNKRLGR	707	0.91
PF3D7_0310300	4	PCTVIIYQYDMLIKNE	660	0.91
PF3D7_0310300	5	HNIYSNSLHNNQSYNN	294	0.90
PF3D7_0310300	5	HQKYNTKNINNNHNNN	245	0.90

Protein ID	Rank	Sequence	Start Position	Score
PF3D7_0310300	5	YYYNQDHYMNNNNNNL	24	0.90
PF3D7_0310300	5	SVHGKDEQTGDINTNH	230	0.90
PF3D7_0310300	5	PQSIQNLSVEMFYRDN	1019	0.90
PF3D7_0320800	1	GCHIMVMDEADKLLSP	202	0.95
PF3D7_0320800	2	TEIQPIPNEIDPSLYT	418	0.93
PF3D7_0320800	3	SAMIKELGKHMKVQCM	145	0.92
PF3D7_0320800	4	LKGITQYYAFVKERQK	269	0.91
PF3D7_0322600	1	MKKEKWKYENNNNNNK	902	0.94
PF3D7_0322600	1	GGMIVGDLDEHHMYNE	664	0.94
PF3D7_0322600	2	EQSILPANEDGVSKNN	679	0.93
PF3D7_0322600	2	KNEIEELIKNNKFLNN	148	0.93
PF3D7_0322600	3	DVSVEDEDDSDYDVSF	70	0.92
PF3D7_0322600	3	YGKISYQNDVTKYMNR	451	0.92
PF3D7_0322600	4	NKDIFNKKKTNKMCNN	829	0.90
PF3D7_0322600	4	SYDIKNDVEEDVSVED	60	0.90
PF3D7_0323100	1	DEKGKDKERNDLYFNN	1273	0.93
PF3D7_0323100	1	NSKYMSDLEINNNNNN	1220	0.93
PF3D7_0323100	2	REEKYNKNKKNNNNND	1181	0.92
PF3D7_0323100	2	FFGVMGNVTPLNNNNN	1121	0.92
PF3D7_0323100	3	EGENMKYNNRNYKKNS	912	0.91
PF3D7_0323100	3	GNICMYDIFEDNINNM	807	0.91
PF3D7_0323100	4	SQKIWYTVKYVHVRSK	1348	0.90
PF3D7_0323800	1	ERYPMKYPEFDGTTNE	949	0.96
PF3D7_0323800	2	EKYWDNPPLHYSKKNN	389	0.95
PF3D7_0323800	3	DKEGKMHLTGKEHYNA	296	0.94
PF3D7_0323800	4	MKQISMIKERNKGLDV	585	0.93
PF3D7_0323800	5	YNSQQYYPSHQQQQQQ	899	0.92
PF3D7_0323800	5	MVDYEYNNKGNIRKND	372	0.92
PF3D7_0323800	5	KGMGNKYERSLNYLKK	353	0.92
PF3D7_0323800	5	AASSNEYNHNNKNTNN	311	0.92
PF3D7_0323800	6	NKINNYHNLPDNKNNM	768	0.91
PF3D7_0323800	6	KGNIRKNDSEKYWDNP	380	0.91
PF3D7_0323800	7	YKKIFINNYSNNDGNN	729	0.90
PF3D7_0323800	7	NGNGYTPLYHYYYDDV	70	0.90
PF3D7_0323800	7	GNKYIPRDRSNNNNNI	155	0.90
PF3D7_0323800	7	YCFIRERKYNKPGGNK	142	0.90
PF3D7_0405300	1	NEKIDKTLTFIKEFYP	1767	0.96
PF3D7_0405300	2	VFIIHDNNDSNNNNNN	759	0.95
PF3D7_0405300	2	NCIYKYRNDFDKNNNI	1286	0.95
PF3D7_0405300	3	VEHIDEAEKKPNLDNP	976	0.93
PF3D7_0405300	3	RYEIPMRNLSRNEKDN	723	0.93
PF3D7_0405300	3	DVEINDDVEINNGVEI	1859	0.93
PF3D7_0405300	3	TGKIIIKVEEEKIYKT	1498	0.93
PF3D7_0405300	4	DAIYSMYIEDISNKNI	527	0.92
PF3D7_0405300	4	IINIKYPDYNNVRKKW	1201	0.92
PF3D7_0405300	5	AKNNMDKDDSNNNNNI	1114	0.91
PF3D7_0405300	6	VVYVERDNLFDIERNN	194	0.90
PF3D7_0405300	6	DDDKYNKYHNNNNNNN	1680	0.90
PF3D7_0405300	6	DKFVYEFYTSNKKENI	1056	0.90
PF3D7_0405300	6	RDKEIHKDDRIKGITS	1026	0.90
PF3D7_0408600	1	DGETIVSLSFNVHDNL	890	0.92
PF3D7_0408600	1	VRDKQKPVVSNVRVSS	867	0.92
PF3D7_0408600	2	YSARLHPIGVRDKQKP	858	0.91

Protein ID	Rank	Sequence	Start Position	Score
PF3D7_0408600	2	GQWYKEPLSPSEWATK	500	0.91
PF3D7_0408600	2	VELATSWDEYPKGLQK	406	0.91
PF3D7_0408600	3	SYEITGIVDGMKIGHP	93	0.90
PF3D7_0408600	3	EKWIQIGQWYKEPLSP	494	0.90
PF3D7_0408600	3	LITNMSGYSSNNYQSW	383	0.90
PF3D7_0408600	3	AIKIKVPCEKCKFINS	159	0.90
PF3D7_0408600	3	YGSTYMTPSAIKIKVP	150	0.90
PF3D7_0408700	1	GSSSETDNESDEEEND	101	0.95
PF3D7_0408700	2	QVISESTSTYNTVKCP	673	0.93
PF3D7_0408700	2	KYTIGLPPYIPWDKTT	370	0.93
PF3D7_0408700	3	YIPWDKTTAYKNAVNE	378	0.91
PF3D7_0408700	4	LGEADSLIDPGYRAQI	259	0.90
PF3D7_0414000	1	DSSIYMEEYEDVDIDE	821	0.92
PF3D7_0414000	2	PQGIQELEEYRTYDKQ	922	0.91
PF3D7_0414000	3	QMLIDEKIKEKNIKES	301	0.90
PF3D7_0414000	3	FDEIDAALDTIHRDNL	1114	0.90
PF3D7_0414700	1	RKLITSTGKMYAGQLK	382	0.95
PF3D7_0414700	2	PGLIDRPEDKRNIMEK	506	0.93
PF3D7_0414700	2	VSDIMNDNIINKQNNN	125	0.93
PF3D7_0414700	3	YIDITKPAISIIGCTN	442	0.90
PF3D7_0422300	1	ISSLTASLRFDGALNV	235	0.93
PF3D7_0422300	2	PASMMAKCDPRHGKYM	298	0.92
PF3D7_0422300	3	TNSAFEPASMMAKCDP	292	0.91
PF3D7_0423700	1	GDEKDESKKVDAGKNN	87	0.90
PF3D7_0424100	1	EDSIQDTDSNHTPSNK	275	0.92
PF3D7_0424100	1	KKINETYDKVKSKCND	211	0.92
PF3D7_0424100	2	KKEIDNDKENIKTNNA	61	0.91
PF3D7_0424100	2	SEEIDDKSEETDDETE	257	0.91
PF3D7_0424100	2	YGKCIAVDAFIKKINE	200	0.91
PF3D7_0513000	1	HSQIRIKCNFHFIDNW	165	0.91
PF3D7_0513700	1	KVLIRDHLYDRNGYLN	660	0.93
PF3D7_0513700	2	HSDICKREHYISFNKE	38	0.91
PF3D7_0513700	3	KKTIFHKGGHNNDLVL	575	0.90
PF3D7_0515600	1	DMKYIPNTEMDNEVND	1134	0.96
PF3D7_0515600	2	VGKSDTGTHKNQGDDP	717	0.95
PF3D7_0515600	3	MKIRITPETYNYKIPQ	467	0.92
PF3D7_0515600	3	LKSIQIRCGIDKLQTF	436	0.92
PF3D7_0515600	3	STTNQTMDTQNKQVQN	104	0.92
PF3D7_0515600	4	TYAEQYPLSFYLNYYK	825	0.90
PF3D7_0515600	4	EEEKGDKKDHNNDDDK	314	0.90
PF3D7_0515600	4	NISGEYCKKYNALLNL	218	0.90
PF3D7_0515600	4	NVNAYYKDHNDYRKNK	1210	0.90
PF3D7_0515600	4	YESIMGLLFPNIRSHK	1192	0.90
PF3D7_0515600	4	SQDIPTKITNKNMAYE	1178	0.90
PF3D7_0515600	4	SQHGIYGIKFIHLTNE	1080	0.90
PF3D7_0518700	1	NTIIPYTGTNNNNNNN	604	0.96
PF3D7_0518700	1	LSPYDYYNYYCSGLIP	430	0.96
PF3D7_0518700	2	QQNISYYNTYNSNCYS	719	0.94
PF3D7_0518700	2	KDEGEYTNDDNKYVND	56	0.94
PF3D7_0518700	2	NNSIDTYNENNNNHKM	545	0.94
PF3D7_0518700	3	NFNIQRYLSNNNNNNN	695	0.93
PF3D7_0518700	3	SGLIPTYPIFNYRNVI	441	0.93
PF3D7_0518700	3	PFHNNANTQNDNKCNN	1681	0.93

Protein ID	Rank	Sequence	Start Position	Score
PF3D7_0518700	4	YSSASKTYYCNQYLNE	733	0.92
PF3D7_0518700	4	NNSIISSNYYYNYYNN	288	0.92
PF3D7_0518700	4	DEEQKEPFHNNANTQN	1675	0.92
PF3D7_0518700	5	VSSIDLYNTDNMNYTS	754	0.91
PF3D7_0518700	5	APKIYYHDNMHNLYPI	571	0.91
PF3D7_0518700	5	DENIVKKNYNNNNNNN	1194	0.91
PF3D7_0518700	6	HIIELMVDPFGNYLCQ	863	0.90
PF3D7_0518700	6	KKAEQENTHNNVNMNK	788	0.90
PF3D7_0522400	1	HGSIMKNNSYSEGNTN	2429	0.98
PF3D7_0522400	2	TSSFEKQNTFDKNFNT	2395	0.97
PF3D7_0522400	3	YWAYKYSLESHKKNNS	7151	0.96
PF3D7_0522400	3	QFTYKMPFLYKNYDND	6631	0.96
PF3D7_0522400	3	HVGIINKDLYNVNLNF	2693	0.96
PF3D7_0522400	4	GQRGRNHLFYNEYYNN	918	0.94
PF3D7_0522400	4	YSSIYYTLRHNNEIES	8441	0.94
PF3D7_0522400	4	KEPIFMNPYEDKDKTK	2812	0.94
PF3D7_0522400	5	YKMIGHKKRVDNIYND	8880	0.93
PF3D7_0522400	5	RKTIYEHNWEENQYNY	5873	0.93
PF3D7_0522400	5	IKYIEFYYLYKGGCTK	549	0.93
PF3D7_0522400	5	GFKNDVPPTFNNISNY	515	0.93
PF3D7_0522400	5	KKKGYTSKYADNNNNI	3751	0.93
PF3D7_0522400	5	DEEIDMRNKGIAQNSN	2286	0.93
PF3D7_0522400	6	TKFIIAYATDINKYTK	8397	0.92
PF3D7_0522400	6	FEIINATKFIIAYATD	8391	0.92
PF3D7_0522400	6	EGEEMEDQEFEEHYDE	7688	0.92
PF3D7_0522400	6	ERLLFKRRRMNNNKNK	7592	0.92
PF3D7_0522400	6	FFPIYMPTTDTITTTK	7263	0.92
PF3D7_0522400	6	HSEIIIFYKTRTKNNN	6518	0.92
PF3D7_0522400	6	KKSIYSINSNDNLKCN	6253	0.92
PF3D7_0522400	6	KMCINNKNDYNNKSYD	5470	0.92
PF3D7_0522400	6	KDPNRDDNTYDNNYDN	5223	0.92
PF3D7_0522400	6	HLEKYRTNTNGYRKDT	4725	0.92
PF3D7_0522400	6	NILIRDDNNYDNHNDI	4518	0.92
PF3D7_0522400	6	KLIIYDYNKINICNNI	4320	0.92
PF3D7_0522400	6	YGRNDIYERPSIRKNK	3733	0.92
PF3D7_0522400	6	LSSYPFHIYNNNNNNN	3561	0.92
PF3D7_0522400	6	KGSIVCCYVPVFYKNI	3318	0.92
PF3D7_0522400	6	KKYIDDIDCNNINDKN	2886	0.92
PF3D7_0522400	6	YPKENSYETFRKFSNS	2487	0.92
PF3D7_0522400	6	TKEIKDINGDYTGVSD	2313	0.92
PF3D7_0522400	6	YKEYRKKIKYNKRVKK	217	0.92
PF3D7_0522400	6	YSHLIDREYNSSIDNT	1805	0.92
PF3D7_0522400	6	MERNSADYLSNEKCNK	1737	0.92
PF3D7_0522400	6	ISSALEHKRYDKDDNN	1314	0.92
PF3D7_0522400	7	KSAINTTLKIYSDRNF	8823	0.91
PF3D7_0522400	7	GDKQQLPYTYNGYFYI	8367	0.91
PF3D7_0522400	7	NITYVEKNVSDNNVKK	8249	0.91
PF3D7_0522400	7	NKNIKYGLKMKFSINP	7889	0.91
PF3D7_0522400	7	KVRFWEKYEERKKKNV	7419	0.91
PF3D7_0522400	7	KSLIMFHCYVKVKDVP	6884	0.91
PF3D7_0522400	7	DERIYSKEYFNNIQKG	621	0.91
PF3D7_0522400	7	QQEIEGNKKFSFQMNK	5717	0.91
PF3D7_0522400	7	NESNRHSIYYSNGKNI	5530	0.91

Protein ID	Rank	Sequence	Start Position	Score
PF3D7_0522400	7	HDLDDDDDDDDNDDDS	5202	0.91
PF3D7_0522400	7	NFMSNEGNDPNNNYDN	5185	0.91
PF3D7_0522400	7	TWFIQDDLFMYNNQEN	4990	0.91
PF3D7_0522400	7	NEVIKDDVKPLNVSCS	3539	0.91
PF3D7_0522400	7	HGEIYLQPLIYNYKNS	3212	0.91
PF3D7_0522400	7	SFKIDELSKYKLKLNE	177	0.91
PF3D7_0522400	7	FEEIFYMIVRRNAYNQ	1682	0.91
PF3D7_0522400	8	EINICSGIFGDKQQLP	8358	0.90
PF3D7_0522400	8	KITIKNSAKRKNNKNI	7877	0.90
PF3D7_0522400	8	DNLITEMRKEKNKKDK	7756	0.90
PF3D7_0522400	8	QNRNRKDNYMNNMDNN	7086	0.90
PF3D7_0522400	8	IGLRTYDNMYNIQDNI	6434	0.90
PF3D7_0522400	8	KKKGRTKKEYGGMLNN	6157	0.90
PF3D7_0522400	8	HYKYVNRNKMKKRRNV	5765	0.90
PF3D7_0522400	8	ISEEMDNSHRDERKKQ	5045	0.90
PF3D7_0522400	8	YSHYYMSGHLEKYRTN	4717	0.90
PF3D7_0522400	8	YSLDDTHINNDKNNNN	4502	0.90
PF3D7_0522400	8	RFTIHNKTTMDLINFS	4416	0.90
PF3D7_0522400	8	INMKYHKNDNNNNNNN	3447	0.90
PF3D7_0522400	8	KSDIYENNSFNINYFD	3236	0.90
PF3D7_0522400	8	TVSIFNINLFNCIYLK	3192	0.90
PF3D7_0522400	8	DSCIYKKTKRKKYIDD	2876	0.90
PF3D7_0522400	8	YNNIYSKRKMKKMYDN	2575	0.90
PF3D7_0522400	8	HSIQKYPKLYKAKNKR	2534	0.90
PF3D7_0522400	8	NSCYDLKLYGNKHDNL	2501	0.90
PF3D7_0522400	8	TNKSEYEYKYEYELNE	2443	0.90
PF3D7_0522400	8	DETINEEDLFFINNNN	1925	0.90
PF3D7_0522400	8	DSTEDTFSFNTMERNS	1726	0.90
PF3D7_0522400	8	EIFIEHSILMDPFKSK	1230	0.90
PF3D7_0522900	1	EGTISTATTINNNHYN	998	0.96
PF3D7_0522900	1	CGSKMNHNDMDTKKVI	435	0.96
PF3D7_0522900	2	YCPIKYHNNNNNNNNN	868	0.95
PF3D7_0522900	2	HGEIDYDHNSDDDCNC	1129	0.95
PF3D7_0522900	3	NCMINDNDFNNKNGNF	839	0.94
PF3D7_0522900	3	DTHINMEYRCDSIADD	639	0.94
PF3D7_0522900	3	GHLIYHYIPSIHKVNT	1371	0.94
PF3D7_0522900	4	EKERDYNETYFKYNNE	1209	0.93
PF3D7_0522900	4	NCYEEHNYLYDNIHNN	1143	0.93
PF3D7_0522900	5	HDDNEYDNNFRGKDNN	784	0.92
PF3D7_0522900	5	GMDINHYNTIRESNYD	294	0.92
PF3D7_0522900	5	SSTNQDILENDYGNSF	264	0.92
PF3D7_0522900	6	NMKIDNIRQKDEENCM	826	0.91
PF3D7_0522900	6	KNEIELRGEDGKYANK	602	0.91
PF3D7_0522900	6	MNTKEKCKRFSNNMCP	327	0.91
PF3D7_0522900	6	CKTKMCPYMNTKEKCK	319	0.91
PF3D7_0522900	6	EHETSNDDFFNKELQT	1475	0.91
PF3D7_0522900	6	KGKENGKERNDKNINF	1344	0.91
PF3D7_0522900	6	NFSHRTCSYERNNKDS	1185	0.91
PF3D7_0522900	6	VQKWIHGIHSNKFNDT	1087	0.91
PF3D7_0522900	7	NNINDSTNRYMNNNNS	958	0.90
PF3D7_0522900	7	HGRTEFSPTYTHKNSK	92	0.90
PF3D7_0522900	7	THDKKDISTFDKMLSK	76	0.90
PF3D7_0522900	7	NGDVKSYVTYNHKMNH	728	0.90

Protein ID	Rank	Sequence	Start Position	Score
PF3D7_0522900	7	RCDSIADDESNENMQS	647	0.90
PF3D7_0522900	7	KHHIMFKSKSFNRNIP	237	0.90
PF3D7_0522900	7	THNINHYPTYDKIMEH	1386	0.90
PF3D7_0522900	7	HSNKFNDTHNNNNNNN	1095	0.90
PF3D7_0523400	1	LAKIMKKIIYDFKKNT	493	0.93
PF3D7_0523400	2	KRVIGTGFKFVKTLIK	398	0.90
PF3D7_0532400	1	PHVIDYIYKVGYGDNA	342	0.93
PF3D7_0532400	2	LEASYDAIAYRNAQNR	392	0.91
PF3D7_0532400	2	SMKYIWNEVMDKEKKR	182	0.91
PF3D7_0603600	1	HILSEDDNKRNINVDK	782	0.96
PF3D7_0603600	2	YSFYDYYRKYKKRKVK	741	0.94
PF3D7_0603600	2	DERIKDVLKQKKRKNI	681	0.94
PF3D7_0603600	2	RYGEYCPYLYNNKLSP	448	0.94
PF3D7_0603600	2	SKEGRISNTRDVIKNE	1085	0.94
PF3D7_0603600	3	YGNVLYDNTYMNYYNN	502	0.93
PF3D7_0603600	3	ASSIEWDHKNNIIKNK	1569	0.93
PF3D7_0603600	3	YVLNNYYDDMNYYNIP	1486	0.93
PF3D7_0603600	4	KGKIVTKRYRNCKSNN	98	0.92
PF3D7_0603600	4	VKKIKNKDTSRNRKGS	755	0.92
PF3D7_0603600	5	HNMNNMNICRNENNNE	264	0.91
PF3D7_0603600	5	LALNMDTIHNMNNMNI	256	0.91
PF3D7_0603600	5	ICMEEKHSSHDKDTNR	1762	0.91
PF3D7_0603600	6	YEKYKEYYGDDISDIP	7	0.90
PF3D7_0603600	6	TYMGMMNNGNGNGYGN	489	0.90
PF3D7_0605300	1	SSPIKATCRDMALKSS	6	0.95
PF3D7_0605300	2	SCIKSYATQPRGKQGP	315	0.93
PF3D7_0605300	3	ESLIQHPFIFIHNKNR	299	0.92
PF3D7_0608600	1	YLLIYPHISHNSIAPK	259	0.91
PF3D7_0608600	1	TMYHFNPEKFGYKYNN	236	0.91
PF3D7_0608600	1	YKMNQEDLVNDNKKYS	138	0.91
PF3D7_0608600	2	NSSKHEKNRYDNNLVF	182	0.90
PF3D7_0610200	1	KMEIMKFIERVNEESD	195	0.93
PF3D7_0610200	2	KKKKDDDNYKRKMTNT	728	0.92
PF3D7_0610200	2	DSHIKKSNDKVKNINN	25	0.92
PF3D7_0610200	2	HKERDMQYEENEKSEE	141	0.92
PF3D7_0610200	3	SISDDADYTVDKKKKK	367	0.91
PF3D7_0610200	4	SSEYDSYEKRRKKEEK	712	0.90
PF3D7_0610200	4	GNVIKWQRQRNPSTNE	64	0.90
PF3D7_0616800	1	GKNIIFNITPSPGATT	467	0.92
PF3D7_0616800	2	QSEIYDTVVIGGGVTG	68	0.91
PF3D7_0616800	2	QFLEERYPVFRQLFNS	178	0.91
PF3D7_0616800	2	CGDIETNYSFEKAKFI	124	0.91
PF3D7_0620400	1	KNTSQKKITYDKYNKN	32	0.93
PF3D7_0620400	2	TTAIIDETVYKFEQLI	381	0.92
PF3D7_0620400	3	CSKNNGGCDVNAECTI	464	0.91
PF3D7_0620400	3	YICEYSKCGPNSRCYI	416	0.91
PF3D7_0620400	3	NSSIMNSESYNNIINS	177	0.91
PF3D7_0628200	1	DKKYDTPYYSNERVDF	451	0.97
PF3D7_0628200	2	GTPGYTAPEGGALCDE	2901	0.96
PF3D7_0628200	3	VGSIFMTDVHRKDTSF	1154	0.95
PF3D7_0628200	4	YEQYEDNNDNDNNKND	968	0.93
PF3D7_0628200	4	YSNIEEKDDERKVLKK	670	0.93
PF3D7_0628200	4	DDDDDDDDDNNNNNNN	2693	0.93

Protein ID	Rank	Sequence	Start Position	Score
PF3D7_0628200	4	GELTYYDYVGKNEVIP	2606	0.93
PF3D7_0628200	4	NVIIKHRNEDDKNGLD	2473	0.93
PF3D7_0628200	4	SSDIINVNTNDKISNI	222	0.93
PF3D7_0628200	4	KIKRMRKNYNNNNNNN	1731	0.93
PF3D7_0628200	4	YRRIIKRRKGSSKGNK	1664	0.93
PF3D7_0628200	5	GFVCEKDDKINNYDND	947	0.92
PF3D7_0628200	5	SVMKDEGGEYKKKENM	2643	0.92
PF3D7_0628200	5	YAWGGNHKHMNVERTS	1836	0.92
PF3D7_0628200	6	HMEILKDNEMNITKYY	604	0.91
PF3D7_0628200	6	DEELYYSSYHNNHHNN	477	0.91
PF3D7_0628200	6	KQREKEKCHRDEKCDR	379	0.91
PF3D7_0628200	6	PGSPTYAVKFIYLKVS	2169	0.91
PF3D7_0628200	6	SLIQTSHIPYDAPLAD	2120	0.91
PF3D7_0628200	6	DGYNSSGSRYNNINDD	1990	0.91
PF3D7_0628200	6	LVDILARHARDSTHND	1801	0.91
PF3D7_0628200	6	NKLILRPIEKDKMKST	1179	0.91
PF3D7_0628200	7	STRSDKPLHFTYSDKK	2791	0.90
PF3D7_0628200	7	MGFTQSFQEYDPFDNG	2423	0.90
PF3D7_0628200	7	FYSDNDGLTSKNKENP	2316	0.90
PF3D7_0628200	7	NDKISNILWNDKYIDT	231	0.90
PF3D7_0702300	1	TTTSTTKVTDNNKTNI	109	0.94
PF3D7_0702300	2	LSIWTTLLYSNKNLKC	13	0.91
PF3D7_0702300	3	ESSSTYTNTRLAANNS	93	0.90
PF3D7_0702300	3	NNTKTTSTDNNNNTNT	413	0.90
PF3D7_0702300	3	TTTNTSTTEHNNNINT	209	0.90
PF3D7_0702300	3	TTTNTSTTEHNNNINT	164	0.90
PF3D7_0708800	1	CQGSINRETFEELCSN	326	0.91
PF3D7_0708800	2	DKTEDKGEKKDAKDQE	544	0.90
PF3D7_0708800	2	KSVIKSKDEKKKADDK	530	0.90
PF3D7_0708800	2	KLTAYYENTPDLPSNC	472	0.90
PF3D7_0720400	1	TNEYITCPWHDAKFDI	156	0.96
PF3D7_0720400	2	TTGISPSPSPMKNMNL	330	0.94
PF3D7_0720400	3	CPHYSAPLKSGVLTNE	143	0.93
PF3D7_0720400	4	GECINGPSFDDIPKYE	174	0.92
PF3D7_0720400	5	NGKISTIIKSDKLKTR	9	0.91
PF3D7_0720400	5	SNIIYKNNVYVEKVDT	294	0.91
PF3D7_0720400	6	ASIITLGNNKMASLNE	598	0.90
PF3D7_0726400	1	RTLIKENNYNDVKNNP	3708	0.95
PF3D7_0726400	1	RNDIQTHIIHDELNNT	3188	0.95
PF3D7_0726400	1	IILWTIYNFMNNKKTI	2871	0.95
PF3D7_0726400	2	DDILSGNNTYNNNDNT	900	0.94
PF3D7_0726400	2	CQHEKDSWTFLKYMSP	68	0.94
PF3D7_0726400	2	PSTECSDLEFGDILNE	4321	0.94
PF3D7_0726400	2	EQVIKDNDWYVIYNNN	4250	0.94
PF3D7_0726400	2	EYYISSYEDMNIELDK	295	0.94
PF3D7_0726400	2	LGSSSSDIDMMKTTTT	2628	0.94
PF3D7_0726400	2	SKLITKGYEKTNHKNT	2164	0.94
PF3D7_0726400	3	EVEIFNKDMNDKIDNI	4418	0.93
PF3D7_0726400	3	NEIDSTYITFHEKCNE	3261	0.93
PF3D7_0726400	3	SSEQEDYEKYSYSNNI	2531	0.93
PF3D7_0726400	3	YKEYIEHIQSVTNNKP	2031	0.93
PF3D7_0726400	3	YKIIILYTHHYKDNNF	1968	0.93
PF3D7_0726400	3	GILWQMDPANINNNTK	152	0.93

Protein ID	Rank	Sequence	Start Position	Score
PF3D7_0726400	3	NNEYDINNEYDNNNNN	1486	0.93
PF3D7_0726400	3	NIFIISWTKFKYAYTP	1164	0.93
PF3D7_0726400	4	LFKDDEDDERIKRLTN	733	0.92
PF3D7_0726400	4	NDSYNDINSYDNNNNN	4076	0.92
PF3D7_0726400	4	VKNIFDPTEDDYLKYV	3359	0.92
PF3D7_0726400	4	KESYDDNNVKDKYNEQ	2784	0.92
PF3D7_0726400	4	FFKQIYYDTYIKIKNE	2212	0.92
PF3D7_0726400	4	KKTNKIYRYMNKNKDN	1691	0.92
PF3D7_0726400	4	HNDDDEHNDDDEHNND	1105	0.92
PF3D7_0726400	5	LSRSSFSLTSDVIQNE	956	0.91
PF3D7_0726400	5	NYYCIKKISFDKELCK	795	0.91
PF3D7_0726400	5	FLKYMSPNKIDKALCI	77	0.91
PF3D7_0726400	5	KKRIFDNQLNDNDNDD	579	0.91
PF3D7_0726400	5	DSFLYEPEYNRNKYTS	436	0.91
PF3D7_0726400	5	HKYIQTNHYNDNNKSV	2906	0.91
PF3D7_0726400	5	KGNIKNKNSHTNVGNN	2481	0.91
PF3D7_0726400	5	ETHNEHKNYMVNMKNN	1451	0.91
PF3D7_0726400	6	YSKNQRTKEYDIIINN	4739	0.90
PF3D7_0726400	6	KKEIYNTNDFVDTKKN	3927	0.90
PF3D7_0726400	6	DVVQHKYSSHDIDNNN	3614	0.90
PF3D7_0726400	6	PFFYIYDKDYENVKNI	3347	0.90
PF3D7_0726400	6	YETDIISNHSSNELNN	2579	0.90
PF3D7_0726400	6	ISSYQNFYYYDDDDYP	2546	0.90
PF3D7_0726400	6	CDVERKEQYDDNHKNI	1888	0.90
PF3D7_0726400	6	KKKTRESYDMNKNVFE	1871	0.90
PF3D7_0726400	6	DYIINYYNNNNNNVVI	1845	0.90
PF3D7_0726400	6	YKYIDEHIKYTSCYVT	1622	0.90
PF3D7_0726400	6	EQYISSTQVEKKMENN	1280	0.90
PF3D7_0726400	6	CKSIDTSKGKNSINKI	1051	0.90
PF3D7_0729900	1	DDYIYYTNDSNNKNNI	1035	0.97
PF3D7_0729900	2	LEEIIKTWTCEFVNWP	939	0.96
PF3D7_0729900	2	NVTWEDARKIMKGQDF	3534	0.96
PF3D7_0729900	2	IWLQYMRYYWDSKKKE	1927	0.96
PF3D7_0729900	3	RKLSETINTFEFMVDE	856	0.95
PF3D7_0729900	3	NVDINMNEEYNSMRTS	72	0.95
PF3D7_0729900	4	DSEVDFSPSFNLFLTS	3867	0.94
PF3D7_0729900	5	YQIIVITLEFNEKSCI	4851	0.93
PF3D7_0729900	5	TWGEIIKMHYIKYRND	3732	0.93
PF3D7_0729900	5	AVAILIMNEGDKNVTW	3522	0.93
PF3D7_0729900	5	TFAGACNPPTDAGRNP	2906	0.93
PF3D7_0729900	6	ESLIIKAIRPDKLENC	4273	0.92
PF3D7_0729900	6	ARKIMKGQDFINKVLY	3540	0.92
PF3D7_0729900	6	LWSNVDPKDVKHRLNN	1434	0.92
PF3D7_0729900	6	IVGIMNTYDMDILSKT	1230	0.92
PF3D7_0729900	7	CLSIEGAEWSNKDNCL	4970	0.91
PF3D7_0729900	7	TLLVYDVEKIDAILNS	3833	0.91
PF3D7_0729900	7	LKIIDEKKEEVSSQKN	3383	0.91
PF3D7_0729900	7	YMTPRDFLDFIKHFLK	3369	0.91
PF3D7_0729900	7	NWVKIERITFAGACNP	2898	0.91
PF3D7_0729900	7	EILEMDMIECTKIENN	1808	0.91
PF3D7_0729900	7	NEIKQTENTFDTLDTE	1136	0.91
PF3D7_0729900	8	AVKPQTSSQIKKRINN	3560	0.90
PF3D7_0729900	8	NIPKKNFDELRAMANP	3497	0.90

Protein ID	Rank	Sequence	Start Position	Score
PF3D7_0729900	8	SSYIIRTIPYRAVNII	2588	0.90
PF3D7_0729900	8	YGDNDYPRKMDKFKLL	2482	0.90
PF3D7_0729900	8	KEEIYGKLDNINLEWT	2356	0.90
PF3D7_0729900	8	HILSMTPKDIVGIMNT	1221	0.90
PF3D7_0731600	1	HMLEKTGITEDNYNDR	636	0.94
PF3D7_0731600	2	AVVEHDNGEPNNYLTY	80	0.93
PF3D7_0731600	2	TGPIFVQDTSDNNSES	483	0.93
PF3D7_0731600	3	SGSIFSGYFLEKENTD	532	0.91
PF3D7_0731600	4	MGIPFSPNTKFKVKTW	499	0.90
PF3D7_0731600	4	RWLVKRIISMRKANNN	407	0.90
PF3D7_0731800	1	YGSHKLSCEPDNKVTS	648	0.98
PF3D7_0731800	2	AGNIKSHIHSNNIKNN	543	0.94
PF3D7_0731800	3	HGLSEASRNERCFVED	309	0.91
PF3D7_0802000	1	GECIYKYIVEGANVFI	1113	0.94
PF3D7_0802000	2	SGSYESPYYHHNKDSK	500	0.93
PF3D7_0802000	2	LEEDEKKDTSNNEKNK	1214	0.93
PF3D7_0802000	3	KGANNTTITFDNVMSS	692	0.92
PF3D7_0802000	3	ASSISVHSTKGANNTT	683	0.92
PF3D7_0802000	4	DEKAKDKEMSNNRKNE	788	0.91
PF3D7_0802000	5	EKDGDMNGHNNNNNDN	847	0.90
PF3D7_0802000	5	KQYIDYMCSPDSDILQ	1170	0.90
PF3D7_0802000	5	GGVISSSLEVLAGLVL	1152	0.90
PF3D7_0802900	1	DIEEDHKNEINANINI	29	0.94
PF3D7_0812300	1	YGEISNSVNKYAEQNV	323	0.94
PF3D7_0812300	2	ISAIQFMNLNSDNCNN	345	0.90
PF3D7_0812300	2	IDIIKDDKLNNQYNND	194	0.90
PF3D7_0815500	1	DEERGENDEEDKENDK	46	0.95
PF3D7_0815500	2	LFLITSYYSFIYIYNN	135	0.94
PF3D7_0815500	3	GSKKDMKNEYKSLNNE	27	0.92
PF3D7_0815500	4	FDVITYKLRKEALKNS	192	0.90
PF3D7_0820700	1	QITYESYNIFDSLKTP	692	0.97
PF3D7_0820700	2	LFSMYPHAEMDNYNNP	63	0.95
PF3D7_0820700	3	RDIIWAQEEHMNMGPW	947	0.93
PF3D7_0820700	3	YEIGYSYEHPDALVIW	725	0.93
PF3D7_0820700	4	CKRKMSYVDFGFNEDD	163	0.92
PF3D7_0820700	5	KKMLKMRMAFDKIENF	855	0.91
PF3D7_0820700	5	DEDSQRYIHMGIVDNS	362	0.91
PF3D7_0820700	5	LGVEIDYYDEDSQRYI	354	0.91
PF3D7_0820700	5	DKKYEYDTKMKKRILE	241	0.91
PF3D7_0820700	6	SRRIEASIKQLKKDKP	966	0.90
PF3D7_0820700	6	TYCGTIGFEYMHITNE	213	0.90
PF3D7_0825900	1	AGLEFEAKLGDKKLNK	617	0.95
PF3D7_0825900	1	DFLKNLPYTMNNNSNN	524	0.95
PF3D7_0825900	2	MFEITNDNNPQKKYNN	315	0.93
PF3D7_0825900	3	NKIESTYMEHDFLKNL	514	0.92
PF3D7_0825900	4	LKRANDIIGFNNNNNN	473	0.91
PF3D7_0825900	5	PNKIFTIRKGKKNKTT	563	0.90
PF3D7_0825900	5	FYMYSTLNASFNRLNS	439	0.90
PF3D7_0825900	5	NQHIYENMYENLNQNI	288	0.90
PF3D7_0825900	5	HRRRIYKNPYEHRKTF	260	0.90
PF3D7_0825900	5	RYKSEEYLYPKKKLSK	169	0.90
PF3D7_0828100	1	SGSIYYNGDRRFINCD	45	0.96
PF3D7_0828100	2	NLLYKEPFHFNIYDND	747	0.93

Protein ID	Rank	Sequence	Start Position	Score
PF3D7_0828100	2	NRYIYNYQKEDKNFNE	688	0.93
PF3D7_0828100	2	ESYIQTMGYQRDYLNN	566	0.93
PF3D7_0828100	2	DIIIQMYVTYYNNMLQ	323	0.93
PF3D7_0828100	3	DQTYGQLNENNPEQNN	588	0.91
PF3D7_0828100	4	NKKRKTSTCYNNNNNN	72	0.90
PF3D7_0830300	1	DEEEYEILEGVKNNSD	71	0.92
PF3D7_0830600	1	TVETRNEEDEDNEDDE	307	0.90
PF3D7_0903500	1	NFLTPTTTTNNNNNNN	555	0.95
PF3D7_0903500	1	NTTTNTTTTNNNNNNN	302	0.95
PF3D7_0903500	2	ANMNTTTTTSNNNNSS	758	0.93
PF3D7_0903500	3	TPTVPTLNVLDKGATT	589	0.92
PF3D7_0903500	3	IGNAQEYEENNKNKSN	1078	0.92
PF3D7_0903500	4	VKSNESPFLFNNNNNN	509	0.91
PF3D7_0903500	4	TFLQNDSLFSQKNNNS	410	0.91
PF3D7_0903500	5	DKNIVNKSDEDKNNDD	904	0.90
PF3D7_0903500	5	GEHKENKTEDDKNIVN	894	0.90
PF3D7_0903500	5	MGTGTTSNTAINTNTT	289	0.90
PF3D7_0903500	5	LEEIIDSLQNAHKDDI	1335	0.90
PF3D7_0904100	1	TDLQQRCYEYDLILNN	724	0.97
PF3D7_0904100	2	INTIQKDLKSDNYLEI	112	0.94
PF3D7_0904100	2	DEEYEDDEEENEEYVD	1028	0.94
PF3D7_0904100	3	HMNNQKNYEMNNNNNN	522	0.93
PF3D7_0904100	4	DVNISSDNNNNNNNNN	552	0.92
PF3D7_0904100	5	AKKIIDKYKNSKITDL	711	0.91
PF3D7_0904100	5	YSKEFNRFPSDNYHNK	1105	0.91
PF3D7_0904100	5	EDEYDDDDEEDEEYED	1012	0.91
PF3D7_0904100	6	KGKIYKLNVMGPKKWK	857	0.90
PF3D7_0904100	6	TEDIIDLLCECLEKNF	667	0.90
PF3D7_0904100	6	ERYTPDDIWFLNKINT	415	0.90
PF3D7_0904100	6	EVLQKTMQRADFGINV	274	0.90
PF3D7_0904100	6	GEARSKQEEDRIICNE	21	0.90
PF3D7_0906400	1	LFEAIHSNTSLKTLNQ	480	0.93
PF3D7_0906400	1	TKDIDEEKKCNIYDNI	328	0.93
PF3D7_0906400	2	NKTIEKGEKRDHHKNE	509	0.92
PF3D7_0906400	3	KSNIYTEETNENNLNK	782	0.91
PF3D7_0906400	3	REDNMDPLKKDNNENF	196	0.91
PF3D7_0906400	3	HVHASISNKFKREDNM	185	0.91
PF3D7_0906400	4	HNSINNNNTINMNNNN	557	0.90
PF3D7_0906700	1	ISDSTYKNQHDNDNNN	413	0.92
PF3D7_0906700	2	VFDIDINNEMDNENMN	94	0.91
PF3D7_0906700	2	IVKIEEGLQRNIYNTE	63	0.91
PF3D7_0906700	2	DEEITINVDHTIEQTS	383	0.91
PF3D7_0909000	1	YESIKTSTNYNNVLNI	263	0.96
PF3D7_0909000	1	NGSYDDKYENIKEQTN	175	0.96
PF3D7_0909000	2	NKSGDSNTENNSDCNS	38	0.93
PF3D7_0909000	2	YENKMRKIERRKKKNN	304	0.93
PF3D7_0909000	3	CNSIIEENTINVEDTK	51	0.90
PF3D7_0909000	3	SDLYNDICVCINNLNN	481	0.90
PF3D7_0909000	3	DNTNEKDNTNEKNNTN	390	0.90
PF3D7_0909000	3	DNTNEKDNTNEKDNTN	384	0.90
PF3D7_0909000	3	DNTNEKDNTNEKDNTN	378	0.90
PF3D7_0909000	3	DNTNEKDNTNEKNNTN	360	0.90
PF3D7_0909000	3	DNTNEKDNTNEKDNTN	354	0.90

Protein ID	Rank	Sequence	Start Position	Score
PF3D7_0909000	3	DNTNEKDNTNEKDNTN	348	0.90
PF3D7_0909000	3	DNTNEKDNTNEKDNTN	342	0.90
PF3D7_0909000	3	NKQIIEHNKNNIQNNI	131	0.90
PF3D7_0917500	1	IGIESTTNESNEKDNI	464	0.96
PF3D7_0917500	2	TLANDNKDEMTKFLNE	654	0.93
PF3D7_0917500	3	HRPIYGNEPMNLIPDD	547	0.92
PF3D7_0917500	3	GTTGLHPYYPTQVYYP	497	0.92
PF3D7_0917500	4	QFIIDRYNEYTWFDKN	364	0.90
PF3D7_0922100	1	VVEPDDDDDYDNNNDN	1317	0.97
PF3D7_0922100	2	VERIRRDDRFRNEYDN	523	0.95
PF3D7_0922100	2	TGEKNNTTTTNNNNNN	228	0.95
PF3D7_0922100	3	VGPYQAPHKFVHNNNF	564	0.94
PF3D7_0922100	3	YERDDTKYESDEKKYK	365	0.94
PF3D7_0922100	4	YPFIGAPTIIKDLKNE	584	0.92
PF3D7_0922100	4	KSTNQRDYYKDNKNDT	322	0.92
PF3D7_0922100	4	TNVEEKGNVNDDFVND	1268	0.92
PF3D7_0922100	5	HDNINENDHNNEYNNN	1119	0.91
PF3D7_0922100	6	VVRKKDNNTNNNNNNS	77	0.90
PF3D7_0922100	6	GGIEDSYFSKNNYANI	716	0.90
PF3D7_0922100	6	NEEEQEDEDEDENEDE	448	0.90
PF3D7_0922100	6	KNEIRAKHEYNSNNSG	413	0.90
PF3D7_0922100	6	NMMNQNMTDMNMNSNQ	208	0.90
PF3D7_0922100	6	YFTHMRLSRNDNMEND	147	0.90
PF3D7_0922100	6	YEHVNEPLNFNFIPNM	109	0.90
PF3D7_0927300	1	LGSIGGPGAILAKNNI	622	0.93
PF3D7_0927300	2	SFGPTTAGRMDAYAEV	575	0.90
PF3D7_0930300	1	PPPANSGNTPNTLLDK	343	0.94
PF3D7_0930300	1	INEIKNPPPANSGNTP	337	0.94
PF3D7_0930300	1	CNENNGGCDADATCTE	1655	0.94
PF3D7_0930300	2	NYTGNSPSENNKKVNE	1215	0.93
PF3D7_0930300	3	CTKPDSYPLFDGIFCS	1684	0.92
PF3D7_0930300	3	GPSGPSGTSPSSRSNT	101	0.92
PF3D7_0930300	4	EETVGHTTTVTITLPP	752	0.91
PF3D7_0930300	4	TLSEVSIQTEDNYANL	1154	0.91
PF3D7_0930300	5	TGTSSTSSPGNTTVNT	908	0.90
PF3D7_0930500	1	TTTTTTTITNNNDNNN	777	0.96
PF3D7_0930500	1	GESIYHNVYYDHNNKN	698	0.96
PF3D7_0930500	2	KSHIDDETFFDSCDKS	898	0.94
PF3D7_0930500	2	FECIMCRNKCHIECAP	150	0.94
PF3D7_0930500	3	KKWGGGTPTSQANNNT	525	0.93
PF3D7_0930500	3	HEKADHHHHNNKKKKK	1136	0.93
PF3D7_0930500	3	SEYISDHEKADHHHHN	1130	0.93
PF3D7_0933600	1	GLWISSGSKYENKKNN	66	0.93
PF3D7_0933600	2	VGCADYFTSFNTCYNN	339	0.91
PF3D7_0933600	2	CGSEIIIRDDDSGPNA	268	0.91
PF3D7_1002000	1	SEEEKTPTSNNIRKKR	238	0.90
PF3D7_1002100	1	MKIYYDNIDMNALSNE	95	0.92
PF3D7_1002100	2	KGSSGGKTSSNKESNE	325	0.90
PF3D7_1002100	2	IVTITYNNYTYNGYNN	32	0.90
PF3D7_1002100	2	TEEKNDTGDIKKEENK	184	0.90
PF3D7_1010700	1	YWEIGENVYLKNINQN	143	0.93
PF3D7_1012300	1	REHGLMYDDMYSDKDP	67	0.93
PF3D7_1012300	2	FKFIRAPYERWQFCAT	47	0.91

Protein ID	Rank	Sequence	Start Position	Score
PF3D7_1012300	3	HPSEQNYDPYIPYLAP	117	0.90
PF3D7_1020300	1	NNVVEEYPDPNKMLCN	66	0.94
PF3D7_1020300	1	DLSIYSEYDYDNKKKT	109	0.94
PF3D7_1020300	2	VYVEHNDNRFNAYNNS	322	0.93
PF3D7_1020300	3	HNLITADTDGRLCNWS	530	0.92
PF3D7_1020300	3	KKEIKGGNKNNEINKN	274	0.92
PF3D7_1020300	4	KHTNDTSIHNDDKKKE	261	0.91
PF3D7_1020800	1	LGDIIMTVESDKADMD	82	0.93
PF3D7_1020800	1	VNIGNALGLPDSLLTP	504	0.93
PF3D7_1020800	1	MVLPSASELMRQNKLN	356	0.93
PF3D7_1020800	1	YGYINFGTFSNVVNNS	24	0.93
PF3D7_1020800	2	PKTVDMTNIQKSIKNN	419	0.90
PF3D7_1020800	2	TPNRITYEDVDAFLNG	380	0.90
PF3D7_1020800	2	DGDINKTENEIKVLNP	141	0.90
PF3D7_1021100	1	YDHNMDTNTNNNTNNN	625	0.96
PF3D7_1021100	2	DEKSGMHNDNNNNNNN	234	0.95
PF3D7_1021100	3	LFMKNDPLEANSFLNV	1663	0.93
PF3D7_1021100	4	FKYYDTIITINNLCNE	814	0.92
PF3D7_1021100	5	HIMIDVFNEFFKYYDT	804	0.91
PF3D7_1021100	5	ICLYNEPIYFRYLLNF	176	0.91
PF3D7_1021100	5	EDTYEFLTYFNKNINT	1134	0.91
PF3D7_1021100	6	KSEINELNPNNNLLYD	390	0.90
PF3D7_1021100	6	LKEIFYNISNENYSNM	293	0.90
PF3D7_1021100	6	MSGSSELLYMKNNVCT	1369	0.90
PF3D7_1030200	1	PMSSPMGSHMGPNLNP	369	0.95
PF3D7_1030200	2	MGSPMGPNMGSPMSSP	358	0.94
PF3D7_1030200	3	IGTYWYYETDMSWNSI	186	0.91
PF3D7_1030200	4	SASYSNIMPFVKGMNN	281	0.90
PF3D7_1030900	1	YVLINEDTCGKKVVCD	50	0.91
PF3D7_1030900	1	GYLIQMSNHYECKCIE	33	0.91
PF3D7_1030900	1	YYTCKEDPSSNGGGNT	172	0.91
PF3D7_1033200	1	TGQIPTPTESSHGISD	216	0.94
PF3D7_1033200	2	ESKGTTPESNFDSKTP	262	0.92
PF3D7_1033200	2	DGKKDTSTNDMDPLNP	231	0.92
PF3D7_1033200	3	EPIIVPSYYPTTGPNP	284	0.91
PF3D7_1033200	3	NSSEDKPTSESKGTTP	253	0.91
PF3D7_1033200	3	TVKIVSKRVPVKSKSS	110	0.91
PF3D7_1033200	4	QEIIDEKDEKVKEKPA	89	0.90
PF3D7_1034000	1	QFMYIDPKTPPNSNNN	583	0.98
PF3D7_1034000	2	KITIQAVNCNNNNMNN	280	0.96
PF3D7_1034000	3	SVMIQHAWTYQALIHD	257	0.92
PF3D7_1034000	4	KKIIYGTTDFVRPENF	636	0.91
PF3D7_1034000	4	HDKIWKILIYDKEGQN	37	0.91
PF3D7_1034000	5	KMKNINKNEHTNNANN	354	0.90
PF3D7_1034000	5	SYDIDTNDTFFKNNCH	319	0.90
PF3D7_1034000	5	HEKIYELLNMRYTNNY	220	0.90
PF3D7_1034400	1	IMEIDKPNIPKINANT	453	0.93
PF3D7_1034400	1	RFMQSSFCRFSNIKTK	10	0.93
PF3D7_1034400	2	AHARDDFPERDDKNYL	573	0.91
PF3D7_1035300	1	HETVEHEETVSQESNP	453	0.94
PF3D7_1035300	1	TEEIIDDVPSPKHSNH	416	0.94
PF3D7_1035300	2	KAIPQEPVVPTLNENE	1088	0.93
PF3D7_1035300	3	DLQIIEPNFVDSQPNP	727	0.92

Protein ID	Rank	Sequence	Start Position	Score
PF3D7_1035300	3	HHENFSSEVSNSELNE	542	0.92
PF3D7_1035300	3	SNPEKADNDGNVSQNS	466	0.92
PF3D7_1035300	4	KDTISTEPFPNQKHKD	170	0.91
PF3D7_1035300	4	DQEGQTHSELNPETSE	105	0.91
PF3D7_1035300	5	QIKIVQENKPNKKETP	1121	0.90
PF3D7_1035300	5	SELNPETSEHSKDLNN	112	0.90
PF3D7_1036400	1	DGSIKPEQKEDKSADI	1606	0.93
PF3D7_1036400	2	LQEQQSDLERTKASTE	227	0.91
PF3D7_1036400	2	YDEHIKKYKNDKQVNK	1739	0.91
PF3D7_1036400	2	LQEQQSDLERTKASTE	1247	0.91
PF3D7_1036400	2	LQEQQSDLERTKASTE	1196	0.91
PF3D7_1103800	1	ETNIIDPNIFNINDNK	3295	0.95
PF3D7_1103800	2	KSTYYTYNNNDKSLSE	2610	0.93
PF3D7_1103800	2	ASHANIKNQFNNNNNN	2587	0.93
PF3D7_1103800	2	DEEEYTDCVGNNENTT	2397	0.93
PF3D7_1103800	2	TECGSANVDIKEKNNI	2207	0.93
PF3D7_1103800	3	KRDINSKLTHGFNQNN	454	0.92
PF3D7_1103800	3	PSKISNHISFNTQNNT	3241	0.92
PF3D7_1103800	3	KSEQEKKDTEDGNNTS	2291	0.92
PF3D7_1103800	3	HLRQLLQPTSTKDCND	1162	0.92
PF3D7_1103800	4	GKLIKNNLLMDYGNSL	90	0.91
PF3D7_1103800	4	YDQIQKKLPNNLNANN	587	0.91
PF3D7_1103800	4	SQHINNDISSNINDNI	353	0.91
PF3D7_1103800	5	KDTEDGNNTSEKKKDK	2297	0.90
PF3D7_1103800	5	NGSATAEISDNKDNTI	2248	0.90
PF3D7_1103800	5	TVCIYKAVEGACTVLN	2182	0.90
PF3D7_1103800	5	DDTYKNDIKYDENKND	1841	0.90
PF3D7_1103800	5	EQKEQKGQEEQKGQKE	1760	0.90
PF3D7_1106300	1	SWYWEYPYHYAPLCSD	609	0.96
PF3D7_1106300	2	PNCITTGTDFMYKINL	164	0.93
PF3D7_1106300	3	NGVIHHCTHANKEKLP	82	0.91
PF3D7_1106300	3	TKEYYMKYESLNSNDF	426	0.91
PF3D7_1106300	3	NVEYENNEEINNEINN	324	0.91
PF3D7_1106300	3	YKFIKSYNLYNKRKHQ	25	0.91
PF3D7_1107900	1	GKMSQTKKENDKNVEE	614	0.95
PF3D7_1107900	2	APLNIADGYNNNNNNN	1382	0.94
PF3D7_1107900	3	KKDINDTCEKDYTSNK	768	0.93
PF3D7_1107900	3	DCVKHFTYLHDKKQNI	1787	0.93
PF3D7_1107900	3	YFSYDSSSGYDSYDCV	1774	0.93
PF3D7_1107900	3	GYMYESYLQKNEDNND	1537	0.93
PF3D7_1107900	4	YAFTTTPIHNNNNNNN	437	0.92
PF3D7_1107900	4	QPKINYPPHGNNNFPT	1424	0.92
PF3D7_1107900	4	HDPISEPKYNRKFIYG	113	0.92
PF3D7_1107900	4	KRYREEHNKDRYKVCK	1034	0.92
PF3D7_1107900	5	CEKDYTSNKMNKIKNN	775	0.91
PF3D7_1107900	5	DKDIKDGCHFYDMTNS	732	0.91
PF3D7_1107900	5	KSPIQLTLSGNEYITK	1086	0.91
PF3D7_1107900	6	DERIMKQIERDDISKK	311	0.90
PF3D7_1107900	6	SRSYMTKQDRINALNL	26	0.90
PF3D7_1107900	6	SLMIDMSRSYMTKQDR	20	0.90
PF3D7_1107900	6	TEFETTTGKIVIYENS	1247	0.90
PF3D7_1108100	1	NYDIQSDDDDNNNINI	1191	0.94
PF3D7_1108100	2	ERTNIYSDKDDVSNNS	523	0.93

Protein ID	Rank	Sequence	Start Position	Score
PF3D7_1108100	2	KKDIDEKDNIDNKDNI	405	0.93
PF3D7_1108100	3	HSDIFFSSYPSKSKDN	634	0.92
PF3D7_1108100	4	RGIYMSNECKREMKNF	928	0.91
PF3D7_1108100	4	YHVYDKNDHMESLKNN	884	0.91
PF3D7_1108100	4	TYNNTYKDEYNNTYNN	675	0.91
PF3D7_1108100	4	KENTTDPYHYTKGCKK	1280	0.91
PF3D7_1108100	4	HKFYYRHIHSNIKDNE	1210	0.91
PF3D7_1108100	5	MCNISRDINNNNNNNN	61	0.90
PF3D7_1121600	1	TNKGTGSGVSSKKKNK	25	0.92
PF3D7_1121600	1	DVTPEQPQGDDNNLVS	143	0.92
PF3D7_1125100	1	CPSRDDFYVFTKKVPI	55	0.94
PF3D7_1127500	1	KFLIFNLLLYLFRCNT	4	0.90
PF3D7_1128300	1	NFCIDYDDKSLKFRNK	893	0.95
PF3D7_1128300	1	KSSQMATMKHNANTWA	1528	0.95
PF3D7_1128300	2	LCEPTDSTTNNQNKNA	825	0.94
PF3D7_1128300	3	TDPYYTIHMSDKKMNT	83	0.92
PF3D7_1128300	3	KHLIKEQQEFFKTLPD	596	0.92
PF3D7_1128300	4	NGKINKAEKPTKKNKT	763	0.91
PF3D7_1128300	4	CEILKNYVQEDKIYKP	356	0.91
PF3D7_1128300	4	KELYCYKKHMNTISSP	1405	0.91
PF3D7_1128300	4	SNLIYYINDFHELCKD	1305	0.91
PF3D7_1128300	4	CLNINTYYYFIKIHTN	1231	0.91
PF3D7_1128300	4	SIPISLYNSFDKNLIE	1193	0.91
PF3D7_1128300	5	LEEICSQQFHYARLST	618	0.90
PF3D7_1128300	5	SIVSAPTCVYNEMKNK	440	0.90
PF3D7_1128300	5	HELCKDVDENIKNVQE	1315	0.90
PF3D7_1128300	5	ISKIIKSCKNNNITNL	1148	0.90
PF3D7_1129100	1	KFFQIYRDPNNKNQNE	150	0.94
PF3D7_1129100	2	FGIIQNPFENGQPTFP	400	0.91
PF3D7_1129100	2	NFENMMKGVFGIIQNP	391	0.91
PF3D7_1129100	3	VFSITTSNGFTCSINK	65	0.90
PF3D7_1129100	3	EQTQEQTEEQEEEQNE	307	0.90
PF3D7_1130700	1	HIDQQDQNESNVHLNN	675	0.96
PF3D7_1130700	2	DDSIITPSNHNNLKCD	223	0.95
PF3D7_1130700	3	DMKISNQDVSNNNNNM	712	0.94
PF3D7_1130700	4	NKLINEKEECINNINE	1172	0.93
PF3D7_1130700	4	DVGGDDDYDYNDFQNT	1039	0.93
PF3D7_1130700	5	DVGGDGDVGGDAGGDD	1023	0.92
PF3D7_1130700	5	MERRGKNQTNDATLNE	1	0.92
PF3D7_1130700	6	KKCKETYLYLCANSNI	775	0.91
PF3D7_1130700	6	YEDIKEKLKEKQINCK	458	0.91
PF3D7_1130700	6	SDSEQEYNTSNSNNTS	1523	0.91
PF3D7_1130700	7	KSNIMDCICFALGINN	325	0.90
PF3D7_1130700	7	GSYEQEEIEDNEYINI	188	0.90
PF3D7_1133400	1	MDEPQDYGKSNSRNDE	579	0.94
PF3D7_1133400	2	RFFVCKCVERRAEVTS	503	0.92
PF3D7_1133400	3	PEASFWGEEKRASHTT	598	0.91
PF3D7_1133400	3	YKRKGNAEKYDKMDEP	567	0.91
PF3D7_1133400	3	ADIPEHKPTYDKMKII	533	0.91
PF3D7_1133400	3	MVSNSTCRFFVCKCVE	496	0.91
PF3D7_1133400	3	APRIFISDDKDSLKCP	476	0.91
PF3D7_1133400	4	IEIVERSNYMGNPWTE	97	0.90
PF3D7_1133400	4	MKIIIASSAAVAVLAT	545	0.90

Protein ID	Rank	Sequence	Start Position	Score
PF3D7_1133400	4	NEHREHPKEYEYPLHQ	42	0.90
PF3D7_1133400	4	AFKADRYKSHGKGYNW	384	0.90
PF3D7_1133400	4	CEDIPHVNEFPAIDLF	320	0.90
PF3D7_1133400	4	LMSPMTLDEMRHFYKD	189	0.90
PF3D7_1136900	1	TGKNNDNNDPNNNNNK	515	0.95
PF3D7_1136900	2	YGEIHIPIKIVTDVNI	1078	0.94
PF3D7_1136900	3	NETEDTLIYHNKNDNS	492	0.92
PF3D7_1136900	4	DEEINYNNNNNNKESP	460	0.91
PF3D7_1136900	4	GSTPDLKQYSDIDLQN	1231	0.91
PF3D7_1136900	5	SVHILAPGHHIYSTIP	934	0.90
PF3D7_1136900	5	KRTINDSLVNAEGAVL	1005	0.90
PF3D7_1143700	1	LFKIWKDIEYDNKLKN	706	0.95
PF3D7_1143700	2	TSYIIEHEKNNNILIF	312	0.92
PF3D7_1143700	3	RYTIQEQEYINNLLNI	48	0.91
PF3D7_1143700	3	MSCKDSANIHILNNND	346	0.91
PF3D7_1143700	4	HFKSITPYFINVKLKD	85	0.90
PF3D7_1143700	4	KFIISYYIPEDNVVEL	179	0.90
PF3D7_1147000	1	KSTQDYDLYHNNMENF	902	0.96
PF3D7_1147000	1	YNTPQSYNTPQSYNTP	257	0.96
PF3D7_1147000	2	NNTPQSYNTPQSYNTP	251	0.95
PF3D7_1147000	3	YNTPQSYNTPQSYNTT	263	0.94
PF3D7_1147000	3	NDSISTCCDNINNNNN	2090	0.94
PF3D7_1147000	3	APEKMNCPQMMANHNF	1802	0.94
PF3D7_1147000	3	DEKIITAYLNNRVVQN	1471	0.94
PF3D7_1147000	4	YKYICNSNSNNNNNNN	1989	0.93
PF3D7_1147000	4	NKLHDMNNERDCNINK	162	0.93
PF3D7_1147000	5	NSNEQNYNDHNKNNNP	2563	0.92
PF3D7_1147000	5	NCTGENEITYLNDLNS	2549	0.92
PF3D7_1147000	5	HVNVDGNNKNDPIDNN	1829	0.92
PF3D7_1147000	5	HDIRTYYNDSTKITNK	1749	0.92
PF3D7_1147000	6	EYTKISKNHQMNNINP	716	0.91
PF3D7_1147000	6	NAHINDNKEYNYQKYP	2510	0.91
PF3D7_1147000	7	HDHNIIYNDVYNHMNP	2581	0.90
PF3D7_1147000	7	TSNIIKSQYNNTNKNM	2049	0.90
PF3D7_1147000	7	IESGNNKLTNDAIHTN	1767	0.90
PF3D7_1147000	7	KKNIYEPNSQNSPNSQ	1611	0.90
PF3D7_1149200	1	FGLPSEKPTFTLEGTP	110	0.95
PF3D7_1149200	2	FSKQYMGTKSVKAKNP	11	0.93
PF3D7_1149200	3	RAAKDAQEEMRKRAEK	316	0.91
PF3D7_1149200	4	SGEQQSDDESGEHQSV	465	0.90
PF3D7_1149200	4	AINYYDAVKDGKYLDD	387	0.90
PF3D7_1149200	4	YEAIPHTREFNPLIVD	152	0.90
PF3D7_1149200	4	EENIEENIEENAEENV	1003	0.90
PF3D7_1205500	1	AQFIQYPERSISLHNT	920	0.95
PF3D7_1205500	2	ERSISLHNTNNPPNTD	927	0.94
PF3D7_1205500	2	SERLHAWVERYITQNP	625	0.94
PF3D7_1205500	2	CSGEFNHITENNHNNN	1696	0.94
PF3D7_1205500	3	HNTNNPPNTDNNNNNN	933	0.93
PF3D7_1205500	3	AGMPYKGYVWNKNANK	754	0.93
PF3D7_1205500	3	HDMISPPIFFDDLTSC	569	0.93
PF3D7_1205500	3	SGSIDYLNDNEESDNN	1246	0.93
PF3D7_1205500	4	LGRRNDQSRCDQGNTI	80	0.92
PF3D7_1205500	4	NELQDDENYNNNNNNI	248	0.92

Protein ID	Rank	Sequence	Start Position	Score
PF3D7_1205500	5	HTCISNNSYHRNSGNT	1072	0.91
PF3D7_1205500	6	GSNNSSGSTNNNGGNN	978	0.90
PF3D7_1205500	6	HCLIYYDGSKDAGERW	861	0.90
PF3D7_1205500	6	FSELIGYDKDDKETTS	610	0.90
PF3D7_1205500	6	DEHDDDDDDDNDEEND	305	0.90
PF3D7_1205500	6	QRRQQEDEDNHRSINE	234	0.90
PF3D7_1205500	6	NSMINNINSHNHIYNN	1717	0.90
PF3D7_1206100	1	TKRLYAPPTFNEVRHI	136	0.95
PF3D7_1206100	2	GSSEQDPYDFFTLSDR	18	0.90
PF3D7_1212100	1	TYMINNINCFNEIMNI	321	0.95
PF3D7_1212100	2	DSNIEYLRTFSNDLTK	431	0.94
PF3D7_1212100	3	TELIKKDNLNNKNDVK	214	0.92
PF3D7_1212100	4	KEEKKTKLTNVLKQNN	161	0.91
PF3D7_1220000	1	KGSIYDSLIHLKPDNE	16	0.95
PF3D7_1220000	2	EHIIMNSCESSNLNTT	221	0.94
PF3D7_1220000	3	TLPPKYSDENDIRNNI	88	0.92
PF3D7_1220000	4	TDNILRPPYNNYESNE	397	0.90
PF3D7_1229100	1	LGTIRSMILFGNKYDE	839	0.95
PF3D7_1229100	2	TAQRSNDNTPNNNNTD	937	0.94
PF3D7_1229100	3	TVLIIAHDASTLSCCD	2058	0.93
PF3D7_1229100	3	ASTIGYCITFSCSLGF	1671	0.93
PF3D7_1229100	3	SGDIKYHKFMVLKQFK	1249	0.93
PF3D7_1229100	4	SKDSTNSTTYIKNNNN	287	0.92
PF3D7_1229100	4	SCEIKTQDHYNNIHCS	1210	0.92
PF3D7_1229100	4	KSITQKETTKNYDNNN	1148	0.92
PF3D7_1229100	5	CSTKNHTNKFHKRKKE	98	0.91
PF3D7_1229100	5	TSSIEHYSTNLNRNVR	53	0.91
PF3D7_1229100	6	NIVINMKNCYFSSKNN	755	0.90
PF3D7_1229100	6	LRKYNKYNKYKNYCCS	72	0.90
PF3D7_1229100	6	DPCISKDIFYNLFCDK	1028	0.90
PF3D7_1245600	1	GDKINIPDDNNNNNNN	972	0.94
PF3D7_1245600	1	NEPIITERQNDLIVTT	1171	0.94
PF3D7_1245600	1	NKCYQDNNDNDNYITG	1028	0.94
PF3D7_1245600	2	TWYIDNKIDEKKKKNE	41	0.93
PF3D7_1245600	2	FHLCKDYNTCHNNENK	207	0.93
PF3D7_1245600	2	RELINYKNKMLSYLNG	1577	0.93
PF3D7_1245600	3	TGSIKIETTMINKSLT	430	0.92
PF3D7_1245600	3	KDIIPDHTTNQNETTC	1211	0.92
PF3D7_1245600	3	YPHIIHMKYMPSINNN	1156	0.92
PF3D7_1245600	3	YITGDDKNMNNNIKNK	1040	0.92
PF3D7_1245600	4	DQHNDIHLTYDELTNC	260	0.91
PF3D7_1245600	4	HDKINHKDNNDNNKDA	227	0.91
PF3D7_1245600	5	DETHIHDNDNDNDDDV	882	0.90
PF3D7_1245600	5	YDDDDDDDDDDDEKNN	684	0.90
PF3D7_1245600	5	FKEPIEINVSDIIENE	1523	0.90
PF3D7_1245600	5	SFSNIAPTNKNNKNYD	1285	0.90
PF3D7_1250100	1	SPHIEHGTLHDDELNP	674	0.97
PF3D7_1250100	2	DGDSITYNEFKKKNPE	2250	0.95
PF3D7_1250100	3	HFIYSLPPYMRNVVFH	2521	0.94
PF3D7_1250100	3	HEERGLKNEHNNYISE	2176	0.94
PF3D7_1250100	3	SEDIYDYITWVRDNTA	1640	0.94
PF3D7_1250100	4	RFRYANYTWERANLNE	2313	0.93
PF3D7_1250100	4	DQKIYDYDDHLIFVYN	1669	0.93

Protein ID	Rank	Sequence	Start Position	Score
PF3D7_1250100	4	HGDIIKGEDNDEVLLI	1411	0.93
PF3D7_1250100	5	DHHIDHHDHHIDHHNH	925	0.92
PF3D7_1250100	5	YRHIKYYMDHYILQYS	3028	0.92
PF3D7_1250100	5	SGTINNLLKDPNNLPP	3011	0.92
PF3D7_1250100	5	HNYIHDDYYMYEKDLN	2824	0.92
PF3D7_1250100	5	DQHHDHHHDHDQHHNN	1921	0.92
PF3D7_1250100	6	FNEQQEQRERKKKAEE	995	0.91
PF3D7_1250100	6	DHHIDHHNHHIDHKSN	932	0.91
PF3D7_1250100	6	DHHIDHHNHHIDHHNH	904	0.91
PF3D7_1250100	6	DHHIDHHNHHIDHHNH	869	0.91
PF3D7_1250100	6	EGYIPHTYDDDSFFTD	729	0.91
PF3D7_1250100	6	LSTNIQNGEGDHINNN	1554	0.91
PF3D7_1250100	6	DSEGSESDVPKDKVKP	1061	0.91
PF3D7_1250100	7	NKTIDNLDKFFNELLN	550	0.90
PF3D7_1250100	7	PTLAELYLYIDKRVTI	3068	0.90
PF3D7_1250100	7	HPELKEEIYFDDNYND	2293	0.90
PF3D7_1250100	7	VLHIYSILRNIKNNNI	1772	0.90
PF3D7_1250100	7	KFREEYELPNVVELTP	176	0.90
PF3D7_1251000	1	HFLIKDKILGDGNDCE	119	0.92
PF3D7_1302100	1	RVSIRRYVRKNQNRYN	70	0.91
PF3D7_1306500	1	YEEIYDAIEDDNNEKK	573	0.97
PF3D7_1306500	2	FYTYDYSIDYDIKKNK	1401	0.95
PF3D7_1306500	3	GKKKFTKKIMNKIQNT	3683	0.94
PF3D7_1306500	3	CKDEYCTLLHDNHLNN	3136	0.94
PF3D7_1306500	3	EDKINNPYLYNNEKDI	303	0.94
PF3D7_1306500	4	SHFIYDLKIYRKKINM	818	0.93
PF3D7_1306500	4	NSLIYDGEWRNCEKNG	3527	0.93
PF3D7_1306500	4	KINIYCYRTYKSYLNN	278	0.93
PF3D7_1306500	4	ENYIYESYFYKFYCTN	242	0.93
PF3D7_1306500	4	KVRIERNMLYNNKNKE	1419	0.93
PF3D7_1306500	5	EKYGRNRYKMDNHYNR	448	0.92
PF3D7_1306500	5	QNTVMANYSYTNNVDC	3696	0.92
PF3D7_1306500	5	IYHINIVEVEDKINNP	294	0.92
PF3D7_1306500	5	KKEIKKNYKMKNCRNK	2748	0.92
PF3D7_1306500	6	DKMGKYYTEYDKLKVY	4214	0.91
PF3D7_1306500	6	IGYWDHNNYSHFGSCN	4153	0.91
PF3D7_1306500	6	TKIIYADKSEYFGAVN	3899	0.91
PF3D7_1306500	6	YPTIYNYPYFPFFINK	2818	0.91
PF3D7_1306500	6	KYEIYHVYLKNNKKNI	2231	0.91
PF3D7_1306500	6	GTYITSKKNNDNYQNH	2032	0.91
PF3D7_1306500	6	KKIKSDIIQFDVEKND	103	0.91
PF3D7_1306500	7	YFKNDKIDKMGKYYTE	4207	0.90
PF3D7_1306500	7	IGYWSNGYRQGNGCLH	3963	0.90
PF3D7_1306500	7	GGENMANNIENNVAND	3618	0.90
PF3D7_1306500	7	NVLIDIIPNYTNNINE	3274	0.90
PF3D7_1306500	7	FIQINYTCEERLRNNL	3211	0.90
PF3D7_1306500	7	KSFSTSNPCNNNNMNE	2981	0.90
PF3D7_1306500	7	SLLIRTKYKHIKNYNF	2889	0.90
PF3D7_1306500	7	SKEQKYRNIQNNHVNE	2004	0.90
PF3D7_1306500	7	TIPIYLNDEMKKEQKR	1936	0.90
PF3D7_1306500	7	QDNQQFPPSSNKVNTS	1189	0.90
PF3D7_1311000	1	YFSNKAKEKFREFCSD	31	0.91
PF3D7_1312600	1	HSTSDDSSLYRPKEEN	318	0.92

Protein ID	Rank	Sequence	Start Position	Score
PF3D7_1314500	1	SCIISIFNRSDKKKEP	59	0.95
PF3D7_1318300	1	AHLINKRNTMDNIVNN	817	0.97
PF3D7_1318300	2	IGPIDFPDFNIKRKND	780	0.96
PF3D7_1318300	3	GGIVENNIKNDKYLDD	623	0.95
PF3D7_1318300	3	NEEINSHDLRRNNKDI	468	0.95
PF3D7_1318300	4	KKSSYIDNVYNKKLNL	44	0.93
PF3D7_1318300	4	DDMKTSKNEEDALLTH	1188	0.93
PF3D7_1318300	5	TYTKQTYNQKMKRENS	1322	0.91
PF3D7_1320600	1	AQIWDTAGQERYRAIT	63	0.90
PF3D7_1320800	1	YESINERTERRVRMLP	184	0.96
PF3D7_1320800	2	TFTISNGGVFGSMLST	336	0.93
PF3D7_1320800	3	TITIIDTDKVSVDINS	78	0.91
PF3D7_1320800	3	LCEIDTSVEPPEDICK	116	0.91
PF3D7_1325200	1	IVEIIEAYLKDLKRVL	248	0.92
PF3D7_1325900	1	TGNIISPDKKDFNCQT	1088	0.96
PF3D7_1325900	2	TGNIILPDKKDFNCQT	1058	0.95
PF3D7_1325900	2	TGNIILPDKKDFNCQT	1043	0.95
PF3D7_1325900	3	NAYTQTMNTFYNNNNN	1278	0.94
PF3D7_1325900	3	TTNIISPDKKDFNCQT	1133	0.94
PF3D7_1325900	4	KKEIIMKDDKDEYTYT	2439	0.93
PF3D7_1325900	4	YSKIDSYIKHINNSCN	2336	0.93
PF3D7_1325900	4	YGELIDMDNMDNMNNV	225	0.93
PF3D7_1325900	4	QVEGKDGDKKHNNMND	19	0.93
PF3D7_1325900	4	KKIQVTPTNMNAYTQT	1268	0.93
PF3D7_1325900	4	TYNIISPDKKDFNCQT	1118	0.93
PF3D7_1325900	5	NESLQGYYYFEKGNNL	667	0.92
PF3D7_1325900	5	ENMIMSYNNMNESLQG	657	0.92
PF3D7_1325900	5	LLRIISINQHDKIDTF	633	0.92
PF3D7_1325900	5	IKYIIHTILFFKNNDP	1846	0.92
PF3D7_1325900	5	MDTNVYKDENNNNNNN	1797	0.92
PF3D7_1325900	5	PKDIQDNINHDNDLRI	1557	0.92
PF3D7_1325900	5	TKLCDHNFYGDTKLCD	1458	0.92
PF3D7_1325900	5	EKAIIKYSERDNMMMS	1367	0.92
PF3D7_1325900	5	KKYIYIYNTNDEYHTD	1297	0.92
PF3D7_1325900	5	NCYIKCTQTNDVQRND	1225	0.92
PF3D7_1325900	5	TDNIILPDKKDFNCQT	1103	0.92
PF3D7_1325900	5	TDNIILPDKKDFNCQT	1073	0.92
PF3D7_1325900	6	IFYIYKKIYKNAYINI	2600	0.91
PF3D7_1325900	6	PSRISSNSIFHFKQTD	2512	0.91
PF3D7_1325900	6	IKPIDDNTKRDIHQGE	2114	0.91
PF3D7_1325900	6	DKYKYTQNVYNKKVCV	1904	0.91
PF3D7_1325900	6	IIEKKITTTRTNTLDP	1663	0.91
PF3D7_1325900	6	MFLINIFNEYINYYNI	1601	0.91
PF3D7_1325900	6	AKMCDDNFYDDTKLCD	1447	0.91
PF3D7_1325900	7	QGESKDINHMKFEKKD	91	0.90
PF3D7_1325900	7	ENYISDLNKNNKNNKN	440	0.90
PF3D7_1325900	7	NTSIQSSTSPQKKKKN	2716	0.90
PF3D7_1325900	7	HKKINNCINQINKLND	2462	0.90
PF3D7_1325900	7	DKDEYTYTTNDEHNNH	2447	0.90
PF3D7_1325900	7	EIMIEEYRTCLHENKR	2270	0.90
PF3D7_1325900	7	VVIPNDKLYMNTYTNN	2242	0.90
PF3D7_1325900	7	TKLCDDNFYDDTNLCD	1469	0.90
PF3D7_1325900	7	YNTNDEYHTDMKYSNK	1303	0.90

Protein ID	Rank	Sequence	Start Position	Score
PF3D7_1325900	7	KDIINVPETYCKNNYD	105	0.90
PF3D7_1326500	1	HIHIYDKRDNNVYNNI	1868	0.97
PF3D7_1326500	2	YVSNDNTPYYLNNKNE	79	0.95
PF3D7_1326500	3	GITINNTTNNNNNNNI	237	0.94
PF3D7_1326500	4	SPEIYSDNTSTKLYNL	1973	0.93
PF3D7_1326500	5	NEDIIKDMSSNSKNND	693	0.92
PF3D7_1326500	5	ETICEHKPTFHYNENN	383	0.92
PF3D7_1326500	5	NQIIDETNLRNNNSYE	2047	0.92
PF3D7_1326500	5	LGEHNHDITSDTNNSP	1959	0.92
PF3D7_1326500	5	TNLIEDCCNNINKLNS	1589	0.92
PF3D7_1326500	6	KGCEHSRNSNDNSLFD	426	0.91
PF3D7_1326500	6	TKLINSCNTSMNPNET	125	0.91
PF3D7_1326500	6	CSLYNEHILYNKYHKQ	1176	0.91
PF3D7_1326500	6	YNKRIIKYEHDMIANN	1037	0.91
PF3D7_1326500	7	EQHIDIKYNNHVKDNN	956	0.90
PF3D7_1326500	7	GCLNIDKQHMNELNNI	831	0.90
PF3D7_1326500	7	SSKIIDSPLSYEQDNM	452	0.90
PF3D7_1326500	7	HYFISQKEGSDNINNQ	1840	0.90
PF3D7_1326500	7	KECVKQDIHHNNELTQ	1809	0.90
PF3D7_1326500	7	KSKIVKVIERNMNKNI	1621	0.90
PF3D7_1326500	7	KKFKEYYNIYDNKNNN	1560	0.90
PF3D7_1326500	7	QDTINDYRQYEKDINT	1330	0.90
PF3D7_1335900	1	EGKGENPNGFDLDENP	334	0.97
PF3D7_1335900	2	HPEREEHEKPDNNKKK	488	0.95
PF3D7_1335900	2	PNPPNPPNPPNPPNPP	353	0.95
PF3D7_1335900	3	DRYIPYSPLSPKVLDN	425	0.94
PF3D7_1335900	3	NFAVEKPNENIIDNNP	310	0.94
PF3D7_1335900	4	SGSIRRHNWVNHAVPL	56	0.93
PF3D7_1335900	5	ERKQSDPQSQDNNGNR	441	0.92
PF3D7_1335900	5	ENPENPPNPPNPPNPP	347	0.92
PF3D7_1335900	6	PEDSEKEVPSDVPKNP	380	0.91
PF3D7_1335900	6	PDSIQDSLKESRKLSD	165	0.91
PF3D7_1335900	7	QGINVAFNRFLVGCHP	192	0.90
PF3D7_1342100	1	RRCIRKYCINKKIVNP	4	0.95
PF3D7_1342100	2	IGMSVLPLQFINNQSP	821	0.94
PF3D7_1342100	2	DVIIIAGKEYGCGSSR	777	0.94
PF3D7_1342100	3	VVGTDSHTTMINGLGI	214	0.92
PF3D7_1342100	4	AGMIHKTSEAYKFLKT	695	0.91
PF3D7_1342100	4	ATISNMAPEYGATVGF	308	0.91
PF3D7_1342100	5	YGCGSSRDWAAKGPNL	786	0.90
PF3D7_1342100	5	NSTYIHKPPYFENMTL	651	0.90
PF3D7_1342100	5	GLPISMTLPEVVGINV	245	0.90
PF3D7_1343300	1	DSPRSKKKRFDAHKNI	372	0.96
PF3D7_1343300	2	LGAIGAYNNSNNNNNQ	773	0.95
PF3D7_1343300	2	NWVNKKHNEFLENLNP	663	0.95
PF3D7_1343300	2	YTGIDHPSTLKKYKTP	208	0.95
PF3D7_1343300	3	AHLARTPQESNADDFI	714	0.94
PF3D7_1343300	3	CITINNYVNPPKYINN	240	0.94
PF3D7_1343300	4	ERHNYEYNHNKNMMSP	139	0.92
PF3D7_1343300	5	THTVIEIQTFAKKLSD	855	0.91
PF3D7_1343300	5	KSDPCDLDSIDYEVNP	431	0.91
PF3D7_1343300	6	KIALNDDESNNKSVNE	498	0.90
PF3D7_1343700	1	DSYIITGGENGEVLNS	680	0.97

Protein ID	Rank	Sequence	Start Position	Score
PF3D7_1343700	2	SCHFFSPDTNEWQLGP	695	0.91
PF3D7_1343700	2	KQIKKEKEEHRKRFDE	289	0.91
PF3D7_1346000	1	KCDTYDKNDMNYPVNI	441	0.93
PF3D7_1346000	2	KDILNCYNECNNNNNN	131	0.92
PF3D7_1346000	3	QREIETNEYKTNHENN	19	0.91
PF3D7_1346000	4	HIHVTDLLTMEKKITR	513	0.90
PF3D7_1346400	1	KQMIIDHDEYHYVKYI	5274	0.97
PF3D7_1346400	1	KEEYTAEDTFSERRNS	1935	0.97
PF3D7_1346400	2	LQSVVDHNDHDNFQWK	767	0.96
PF3D7_1346400	2	KGTTTTTTTSGSNNNN	5473	0.96
PF3D7_1346400	2	DIIAGGPTTNIANNNN	4353	0.96
PF3D7_1346400	3	NRMYDKRGDHRKDYND	4778	0.95
PF3D7_1346400	3	TKIIEIMNEANKSRNQ	474	0.95
PF3D7_1346400	3	GSKIYSDSSSRVKGNK	4548	0.95
PF3D7_1346400	3	KKEIDHYNDKDNKYVS	3485	0.95
PF3D7_1346400	3	IKTAFDTNTPVAIITF	2493	0.95
PF3D7_1346400	4	CGEYNDEDDEDKNNDN	988	0.94
PF3D7_1346400	4	TIKQDDHKNMNADSNK	954	0.94
PF3D7_1346400	4	SQTVCAYLEINHLVTE	831	0.94
PF3D7_1346400	4	NSSSSSHNYYGNKSNI	4374	0.94
PF3D7_1346400	4	NRKEMERNDHNKIKNI	3212	0.94
PF3D7_1346400	4	VGPVIPPLTFIHKKIK	3144	0.94
PF3D7_1346400	4	CTRSQYKNTSNENKNK	2787	0.94
PF3D7_1346400	5	DYMYEERNKIDALKNK	4508	0.93
PF3D7_1346400	5	TDGIMADDDITNGSIN	4121	0.93
PF3D7_1346400	5	NNCIGEWEPFLEKCNI	3403	0.93
PF3D7_1346400	5	DRTIDKEMDHGKNKLD	2433	0.93
PF3D7_1346400	5	NSEWFKKGYMNFNNFN	2347	0.93
PF3D7_1346400	5	DVLEEDHFMMGNNNNI	1443	0.93
PF3D7_1346400	6	KESISGTRKRSNKNKK	920	0.92
PF3D7_1346400	6	LFMIQEAMTNDDNYDV	621	0.92
PF3D7_1346400	6	HGLIVRTNTSTNTVYK	5933	0.92
PF3D7_1346400	6	LDRSSYYYMMNNKSNI	5170	0.92
PF3D7_1346400	6	LPPIGSYTETNVICKN	4845	0.92
PF3D7_1346400	7	KKRIIDNRNVNNHHNN	660	0.91
PF3D7_1346400	7	DVEIEMDAETLNGINL	5536	0.91
PF3D7_1346400	7	SFTINESDLLRFKKTF	5437	0.91
PF3D7_1346400	7	DVQIDCQLESCEKCVL	5376	0.91
PF3D7_1346400	7	AKLEISDKWEGITLTK	455	0.91
PF3D7_1346400	7	CGIPLEFCFFDGSRNP	4247	0.91
PF3D7_1346400	7	DDDITNGSINNNNNNN	4127	0.91
PF3D7_1346400	7	YESTDDTHSIDERDNL	4015	0.91
PF3D7_1346400	7	ISKTDHTSRSNNMDNN	3913	0.91
PF3D7_1346400	7	NKDIETSEQMNSLNDF	3524	0.91
PF3D7_1346400	7	KRERIHYDEEIIEQNV	2837	0.91
PF3D7_1346400	7	RKIIKMEIEQLWICDP	1370	0.91
PF3D7_1346400	7	PTSITRYLTGVYSKNK	1241	0.91
PF3D7_1346400	8	QQSWWSWVTGNKKEIE	587	0.90
PF3D7_1346400	8	SDLLRFKKTFDRNSND	5443	0.90
PF3D7_1346400	8	ISGGDMSGSNNVYQNY	5416	0.90
PF3D7_1346400	8	GGTGSYKNNSISSSTN	5294	0.90
PF3D7_1346400	8	DKSVTSNNDIKLGLNK	5209	0.90
PF3D7_1346400	8	KGNKDNSNSSNSNNSE	4560	0.90

Protein ID	Rank	Sequence	Start Position	Score
PF3D7_1346400	8	DCHIEEKKLDQKEYNE	3242	0.90
PF3D7_1346400	8	SSKIKDSVYNNLDSND	292	0.90
PF3D7_1346400	8	GDGAINNNVYDSDRTI	2421	0.90
PF3D7_1346400	8	DKNSIGRNTYNSNNIP	2146	0.90
PF3D7_1346400	8	DDIINNHNYNILNDNI	1783	0.90
PF3D7_1346400	8	GGKYMSTNRFSASGIN	1525	0.90
PF3D7_1346400	8	FRSLSTYLEEKSHNNN	1407	0.90
PF3D7_1346400	8	YIHICGPILHFDNLTN	1063	0.90
PF3D7_1346700	1	EIEENDTNPNYNERTI	127	0.92
PF3D7_1346700	2	PGDIIPDCFFQVYQPE	345	0.91
PF3D7_1346700	3	HNLKPDMRERRSIFCT	57	0.90
PF3D7_1346800	1	NVVIKMPSIFINNINC	245	0.94
PF3D7_1346800	2	GCTINNPTLGDHIVLI	61	0.93
PF3D7_1346800	2	QKTYKTFLLSDVLDTP	354	0.93
PF3D7_1346800	3	ELLSSTQYVCDFYFNP	27	0.92
PF3D7_1346800	4	GSSEIYEEVGCTINNP	52	0.90
PF3D7_1354200	1	KNKIQTKNYFDNLFQS	155	0.94
PF3D7_1354200	2	QECITSKNTIIENDNK	48	0.93
PF3D7_1354200	3	YDEISYRRIWINNANA	602	0.92
PF3D7_1354200	4	MNELDKKNDMNKKKKD	237	0.91
PF3D7_1354200	5	KKLNIYSNKNNNNDNN	262	0.90
PF3D7_1360500	1	PQKIEENKTFCIKMNN	277	0.95
PF3D7_1360500	1	YSSYSSPKYGDNENNF	2082	0.95
PF3D7_1360500	1	AYEIDLPYKFVRRLPY	1041	0.95
PF3D7_1360500	2	LEEEYTRNYYREIINI	2718	0.94
PF3D7_1360500	2	IGSINKQTERKPKKKN	2353	0.94
PF3D7_1360500	3	LHLIGDIVDNNIKRKK	1983	0.93
PF3D7_1360500	4	SRSISDIKEGQIIDKE	1829	0.92
PF3D7_1360500	4	IHVSYDTYKYLKEDNT	1708	0.92
PF3D7_1360500	4	FCDIDDFQNMVSTLQP	1551	0.92
PF3D7_1360500	4	SQTIIDINTKSNIHYI	1320	0.92
PF3D7_1360500	5	SIEYEDICLYNIIRNT	625	0.91
PF3D7_1360500	5	NENVDYAYQFDNYDKK	2672	0.91
PF3D7_1360500	5	SGKYSHNNKKNHHHNN	2609	0.91
PF3D7_1360500	5	ESEIMNEEDNKNMLNL	2503	0.91
PF3D7_1360500	5	KKEIVGNLSYDKTKKI	2029	0.91
PF3D7_1360500	5	TFIWKERKVFIKGKGK	1723	0.91
PF3D7_1360500	5	MIDICTHFCSNPYLSI	1434	0.91
PF3D7_1360500	6	TVLGIGTTLNDAYLLK	926	0.90
PF3D7_1360500	6	NTDSEKPKVFNKNKND	371	0.90
PF3D7_1360500	6	LFTIGDAYVATSQPNS	3012	0.90
PF3D7_1360500	6	NNKISKNRYNNNNNNN	2585	0.90
PF3D7_1360500	6	TSSSKDGVSYNFLSDS	2151	0.90
PF3D7_1360500	6	YSKRIDKESRDKISDK	1861	0.90
PF3D7_1360500	6	EHFSDNSTSYDKYINK	1646	0.90
PF3D7_1360500	6	IYKIKDDPTYYNKSKR	1250	0.90
PF3D7_1361700	1	NFHWDDYWANTPKQNI	105	0.92
PF3D7_1361700	2	HHIDHHHGDPRAHLNE	78	0.91
PF3D7_1361700	3	DGTRKPEYDFNNFHWD	94	0.90
PF3D7_1361700	3	HGDPRAHLNEDGTRKP	84	0.90
PF3D7_1365300	1	NHNVSNKNTPNKNKNK	58	0.92
PF3D7_1405400	1	TISIYDNITFDVLNVY	401	0.96
PF3D7_1405400	2	PDQYDNSLVYDDKLNV	1376	0.93

Protein ID	Rank	Sequence	Start Position	Score
PF3D7_1405400	2	DKHEIQYNTPSNMENN	1296	0.93
PF3D7_1405400	2	AEEIKKMYKEKNYVND	1006	0.93
PF3D7_1405400	3	DEIKEYTLTKNVNKND	956	0.92
PF3D7_1405400	3	TKHIRYINEYIKKNVK	631	0.92
PF3D7_1405400	3	LFKIENYYLFNNFKSS	442	0.92
PF3D7_1405400	4	EFLNNYDKTRIHNDNP	1356	0.90
PF3D7_1405400	4	FLEYIQYRKDGKCLND	1266	0.90
PF3D7_1405400	4	TYIIGAKPYNIIKHNL	1024	0.90
PF3D7_1407000	1	KSWIGAPYDKNVKVNI	250	0.98
PF3D7_1407000	2	TKEIIIGNENDGLLNK	80	0.97
PF3D7_1407000	3	RLQIYYHGVFGSVCSE	544	0.94
PF3D7_1407000	3	HAGIYKPNMGNKNNTF	1112	0.94
PF3D7_1407000	3	TIDGRDCVTPCKSKNM	1030	0.94
PF3D7_1407000	4	GCVGGDNNSHGEEQNK	640	0.93
PF3D7_1407000	5	HITSSMKPTFQWIAPT	815	0.92
PF3D7_1407000	5	TGTIEIILLGNNKRSN	174	0.92
PF3D7_1407000	6	TFQWIAPTGFVGFNGK	823	0.91
PF3D7_1407000	6	KSAIHSGVLPNNIAED	722	0.91
PF3D7_1407000	6	SVLCKAAIHSGALSNQ	1214	0.91
PF3D7_1407000	7	CNIIKDKIYGTNIYSP	1197	0.90
PF3D7_1408700	1	HQRIHHDMTHENNKNE	6826	0.97
PF3D7_1408700	2	DEEDDDHNDINNNNNN	3184	0.96
PF3D7_1408700	2	CACVHDNTHNDNNNNN	1638	0.96
PF3D7_1408700	3	SFMKKHDTNDDKNNNN	5666	0.94
PF3D7_1408700	3	NEHIQKDTSDDSSDTT	5024	0.94
PF3D7_1408700	3	LCIGYNDIVSNKGRNN	4842	0.94
PF3D7_1408700	3	TGCVVTSGDIVKALNN	4707	0.94
PF3D7_1408700	3	HVGIYTIEDIKNEKNK	1705	0.94
PF3D7_1408700	3	GGSVKKNVYYNNRMNK	1663	0.94
PF3D7_1408700	3	HKDIIRPSEEEKEKKK	1364	0.94
PF3D7_1408700	4	NIGIQNKGNNNNNNNN	6771	0.93
PF3D7_1408700	4	EEMIINPDLLMKGYQS	5510	0.93
PF3D7_1408700	4	NVKISITNEMKANDNL	4597	0.93
PF3D7_1408700	4	NNGINHHNNYNNNSNN	4229	0.93
PF3D7_1408700	4	HIEKRERKRMFNFLNK	1423	0.93
PF3D7_1408700	5	MYTSTSGNDMLNLLFP	7005	0.92
PF3D7_1408700	5	NSLIDTGYLINNISPK	5436	0.92
PF3D7_1408700	5	SSSSSYYSSSDSRNDV	5117	0.92
PF3D7_1408700	5	DFNVNYYYTYDPRLLI	4277	0.92
PF3D7_1408700	5	SSSSSNFYSFDKNNNL	3975	0.92
PF3D7_1408700	5	DNIIISNNDNMNKDNI	2297	0.92
PF3D7_1408700	5	GMKLKKKLKYDNYIDN	1861	0.92
PF3D7_1408700	5	KRTISNYGNCDIKKDN	1379	0.92
PF3D7_1408700	6	DSSYYMNQKRRKKQKK	846	0.91
PF3D7_1408700	6	TVGPQSPPLNKIRAIP	7066	0.91
PF3D7_1408700	6	PIEISDKNNLRKYLSR	6553	0.91
PF3D7_1408700	6	SGSDSDPILFSSTDSS	5727	0.91
PF3D7_1408700	6	HFHIINYKWYDNIVWI	4797	0.91
PF3D7_1408700	6	NISVRDPMKYRLIINI	3687	0.91
PF3D7_1408700	6	NFIIYIKLFNDKGENQ	2572	0.91
PF3D7_1408700	6	NSEGYEKYHVNISRNI	2504	0.91
PF3D7_1408700	6	SSLINTTSESSYSNCS	2231	0.91
PF3D7_1408700	6	DTESINSNVMNNYLSL	1896	0.91

Protein ID	Rank	Sequence	Start Position	Score
PF3D7_1408700	6	KLKYDNYIDNNNNNNN	1867	0.91
PF3D7_1408700	6	YGNCDIKKDNNGINNN	1385	0.91
PF3D7_1408700	6	YSIYHTYNHRKNGYLD	107	0.91
PF3D7_1408700	7	YNKIELKIYSDLTLNV	648	0.90
PF3D7_1408700	7	KDHINNKSTKNFMNNK	6085	0.90
PF3D7_1408700	7	YSKYKSPLYNKKKEDL	4677	0.90
PF3D7_1408700	7	AKTSINNNNYNNINNN	4475	0.90
PF3D7_1408700	7	NFIRNMKNDYNKYLDY	3866	0.90
PF3D7_1408700	7	EKMDNEDKKRNKILNF	3835	0.90
PF3D7_1408700	7	HVGIDTKLNHDKKDIG	3759	0.90
PF3D7_1408700	7	IHDIDGKDSPMDNSNL	3401	0.90
PF3D7_1408700	7	NKHRNDKNYYDKDDTR	3117	0.90
PF3D7_1408700	7	DYSIYSDVDSCACRLK	2997	0.90
PF3D7_1408700	7	IGEIKHNKKNKKNKNF	2558	0.90
PF3D7_1408700	7	EEQIIVQYERNNMDNM	1821	0.90
PF3D7_1408700	7	NKLIKKRKSYREKSYP	1348	0.90
PF3D7_1409800	1	IGELEKYGVNMNNANE	134	0.95
PF3D7_1409800	2	RMSEQCSYQMNSPYNP	33	0.93
PF3D7_1409800	3	VVFIKDKKPNVNRSNV	83	0.91
PF3D7_1409800	3	SKEEGRPYYHNEITGE	362	0.91
PF3D7_1409800	3	DNMENMDNFDNNNNNN	264	0.91
PF3D7_1409800	3	SNGISKRCAFVNYAYK	187	0.91
PF3D7_1410400	1	GVTYQEYPETMLYNCP	339	0.94
PF3D7_1410400	1	GADEEAPPAPKNTLTP	189	0.94
PF3D7_1410400	2	TSTPSGTQTSGLKSSS	131	0.92
PF3D7_1410400	2	LEEDDSKDDMEFKASP	111	0.92
PF3D7_1410400	3	LTWIYDNIYMIKRYYT	563	0.91
PF3D7_1410400	3	QKEEFEYDENMEKAKQ	241	0.91
PF3D7_1410400	4	KKYSTSHIYFDPTVAS	664	0.90
PF3D7_1410400	4	GTCIGSFGEHHLRLYE	409	0.90
PF3D7_1410400	4	SGLKSSSPSSTKSSSP	140	0.90
PF3D7_1419600	1	LVHIYMQDYMNNVQNK	443	0.94
PF3D7_1419600	2	GEMYQKYLDSVCDNDN	265	0.91
PF3D7_1419600	2	SKELIHKRVHNKNVDE	203	0.91
PF3D7_1430700	1	LGPYKGGLRFHPTVNL	149	0.93
PF3D7_1430700	2	NGYILEPNGFTKEQLN	326	0.92
PF3D7_1430700	3	TTLPMGGGKGGSDFDP	181	0.91
PF3D7_1430700	4	CATQNEINENDADLFI	383	0.90
PF3D7_1433200	1	DEEIKTKNKNNNNNNN	608	0.97
PF3D7_1433200	2	DISSTTTLTYKAVENN	682	0.95
PF3D7_1433200	3	DENGIWNNTESKILNT	737	0.94
PF3D7_1433200	3	SYIIYFTETYIKKKTK	1172	0.94
PF3D7_1433200	4	NIKIKTSIRYRKKTNL	283	0.92
PF3D7_1433200	5	KSLIIINRKGNNEKKK	942	0.91
PF3D7_1433200	5	EFLITEDLSTDTDDNF	815	0.91
PF3D7_1433200	5	KESIESVICQDGIINT	327	0.91
PF3D7_1433200	5	RLTNDDDDDNDVNVVD	139	0.91
PF3D7_1433200	6	KEEKITNTWKMKTQNL	894	0.90
PF3D7_1433200	6	TNCQCEKNENNIKDIK	752	0.90
PF3D7_1433200	6	KWSNHFPNTFNISLDL	311	0.90
PF3D7_1433200	6	ISLYENYYEENIKIKT	273	0.90
PF3D7_1433200	6	HYLQDGYYTNDEKYIK	198	0.90
PF3D7_1433200	6	TETYIKKKTKKKVQDK	1178	0.90

Protein ID	Rank	Sequence	Start Position	Score
PF3D7_1435300	1	NCRKDNHTDHDYDDNN	1717	0.95
PF3D7_1435300	1	HCEIRGQLEINYKKCP	1116	0.95
PF3D7_1435300	2	HIIIDMTIDLDKLQSM	789	0.94
PF3D7_1435300	2	NSDINSNSEFLSNVND	373	0.94
PF3D7_1435300	2	TGRVCPAPCEDACVLS	2612	0.94
PF3D7_1435300	2	TPTCHYPNSSGGGCPL	2564	0.94
PF3D7_1435300	2	TKSISNTNDMNNVSNM	2365	0.94
PF3D7_1435300	2	FSDINFTDDSNLKLNE	171	0.94
PF3D7_1435300	2	NSKQADTIERVNGQNE	1572	0.94
PF3D7_1435300	2	VGLISPPPHHDMYSIE	1268	0.94
PF3D7_1435300	2	TGEGGEAEERIKTANN	1189	0.94
PF3D7_1435300	3	KGAISYPSDSSIFDNA	433	0.93
PF3D7_1435300	3	GWRSPIPVRRDVISCS	126	0.93
PF3D7_1435300	4	YGAILNKDNYYHNNNT	615	0.92
PF3D7_1435300	4	VGSGPAGLTAAQQLNK	2668	0.92
PF3D7_1435300	4	RVLTEACIEFVKTGNE	2077	0.92
PF3D7_1435300	4	CDEADAENESDQNDNN	1594	0.92
PF3D7_1435300	4	AVKQIASARFGVTAYS	1211	0.92
PF3D7_1435300	4	CDSIIGGVTFEFVEKE	1024	0.92
PF3D7_1435300	5	PVTNMENIRMNAYKYG	601	0.91
PF3D7_1435300	5	PPCNTYYYYPKNYKNV	3083	0.91
PF3D7_1435300	5	ELSIIEYGFIKKWIQP	2639	0.91
PF3D7_1435300	5	SPTWYAAHPFRRICHN	257	0.91
PF3D7_1435300	5	MVNIQKDDENDIRIAN	2348	0.91
PF3D7_1435300	5	IRETDDYIHMDQKQLS	2264	0.91
PF3D7_1435300	5	CSFSSRTIVYKGLLTP	211	0.91
PF3D7_1435300	5	LFPGWKYYAEDEMKDD	1494	0.91
PF3D7_1435300	6	ASKIDEYLTNSVSILP	3068	0.90
PF3D7_1435300	6	KENIDNKGLYDSRNEK	3	0.90
PF3D7_1435300	6	SQSQYAKTSGDGKIKK	2459	0.90
PF3D7_1435300	6	DGSDDSDDSDDSDGND	1647	0.90
PF3D7_1437700	1	VESIGDMNRFVAVCKK	248	0.96
PF3D7_1437700	2	GIEVILGYYYDNNFGP	649	0.95
PF3D7_1437700	3	HHTIKNTKIYKALAGN	694	0.94
PF3D7_1437700	3	IGIIGATERKGSVGNS	46	0.94
PF3D7_1437700	4	TFSKIYPIDFISFNDK	779	0.92
PF3D7_1437700	4	KKTIDAIIKDDQYKNI	396	0.92
PF3D7_1437700	5	NKLIEEYYYMKNKSDI	510	0.91
PF3D7_1437700	6	YVCGDKIIALDARFTL	748	0.90
PF3D7_1438800	1	NQLYSTMDDPSARNTN	81	0.94
PF3D7_1438800	2	RNMDTTPNFGDKYYDT	30	0.93
PF3D7_1438800	3	DKYYDTEYVFPNNMTM	40	0.92
PF3D7_1438800	4	PSTYDMYDSYYKSSTY	606	0.91
PF3D7_1438800	4	EESTQNDNPNDKTDDV	579	0.91
PF3D7_1438800	4	SMSISKTYVSKLHVNE	393	0.91
PF3D7_1438800	5	KRKINKYFPDCPILNN	237	0.90
PF3D7_1439400	1	VIKWRGKPVFVKHRTP	246	0.95
PF3D7_1439400	2	PESINSDHHPDFREYR	167	0.94
PF3D7_1439400	3	PEDIQRAKEDDKLIQT	261	0.93
PF3D7_1439400	4	CHGSHYDNSGRIRQGP	319	0.92
PF3D7_1439400	4	YSGYFCPCHGSHYDNS	312	0.92
PF3D7_1439400	5	GRIRQGPAPSNLEVPP	328	0.91
PF3D7_1439400	6	LVAGGTTELDMRTVNP	226	0.90

Protein ID	Rank	Sequence	Start Position	Score
PF3D7_1442300	1	GVKIGERILFHNMDPN	333	0.94
PF3D7_1442300	2	NVIPDKNLSSDKEKNP	348	0.93
PF3D7_1442300	3	PRDICSGLRNKKNAED	275	0.91
PF3D7_1442300	4	TKWISSQGDITCVLNQ	383	0.90
PF3D7_1442300	4	EIHPDADTLYCLKINL	255	0.90
PF3D7_1442300	4	KVEETKKLDDISRLNV	231	0.90
PF3D7_1444100	1	CEEIIKPNDYGSFQIV	6754	0.96
PF3D7_1444100	1	EGTIKTYNEKEGNTND	3402	0.96
PF3D7_1444100	1	RSSIIHTNEKDSKNNM	1610	0.96
PF3D7_1444100	2	DVMIEDFDENNKNAKQ	7062	0.95
PF3D7_1444100	2	HIVIYNPLDENVEMEP	6953	0.95
PF3D7_1444100	2	DVYIQLYPKFNKINSK	6603	0.95
PF3D7_1444100	3	KGLIMGKSVSPKPKGP	7137	0.94
PF3D7_1444100	3	LGSIQMNEVSFTNVCN	7041	0.94
PF3D7_1444100	3	DHIIIYPYQHKFIDIC	4587	0.94
PF3D7_1444100	3	ENVIQMKYVPVNEAKI	4158	0.94
PF3D7_1444100	3	YCDIIWYIDSNSIKDN	4011	0.94
PF3D7_1444100	3	KPRIYTNPYFLFNNNV	3696	0.94
PF3D7_1444100	3	TKSYNDHTTYLKEENV	2995	0.94
PF3D7_1444100	4	SKLIIYYPNETAKNYT	6804	0.93
PF3D7_1444100	4	NSENNDHGKYNNYDDG	6646	0.93
PF3D7_1444100	4	YEIKFAPKKYIKLQIP	6450	0.93
PF3D7_1444100	4	YEYEYEYEYDNNIMNK	5814	0.93
PF3D7_1444100	4	EGLVKSCYRKNKYINN	5613	0.93
PF3D7_1444100	4	DIKIPIKMTFDNYDKE	5383	0.93
PF3D7_1444100	4	FEIIDEKILYDHNHTL	4102	0.93
PF3D7_1444100	4	EEKISGKSKGNYKKNL	3493	0.93
PF3D7_1444100	4	TFHSKMKNDSINKKNN	1494	0.93
PF3D7_1444100	4	DNMQDLDTTFHSKMKN	1486	0.93
PF3D7_1444100	4	VPIYFDINYMNNKLNT	1208	0.93
PF3D7_1444100	5	TNKILGSYSFKFILNI	7011	0.92
PF3D7_1444100	5	SSMIKYPNITNNDKNK	5474	0.92
PF3D7_1444100	5	NKIIKIYPKYQKLYND	436	0.92
PF3D7_1444100	5	HTLIEDDIKMKENNFL	4115	0.92
PF3D7_1444100	5	YSIKDEPNESMEYTNN	3903	0.92
PF3D7_1444100	5	IEFQQIDTSYDLKDNI	3627	0.92
PF3D7_1444100	5	LYDYETDEYYVNENND	354	0.92
PF3D7_1444100	5	NENIDEKKRKRKKKDK	3372	0.92
PF3D7_1444100	5	EEYININLTGDHVLKE	2594	0.92
PF3D7_1444100	5	FEEIKIKLKSTNSCNI	1445	0.92
PF3D7_1444100	5	YELVGQTIYPNIIMNK	1242	0.92
PF3D7_1444100	6	QNLTISYQTCDKEGNP	6857	0.91
PF3D7_1444100	6	GKYNNYDDGEDNNNNN	6653	0.91
PF3D7_1444100	6	KCYIENYDKEHITLNL	5905	0.91
PF3D7_1444100	6	TDIYFPTNHMLNYRND	5759	0.91
PF3D7_1444100	6	TFIIHQKYTNVKKNIK	574	0.91
PF3D7_1444100	6	EGDSISHNSYNDNMTF	5664	0.91
PF3D7_1444100	6	IYMINMSNKHIEYCFE	5451	0.91
PF3D7_1444100	6	NKMYDPNNDNNNNNNN	3580	0.91
PF3D7_1444100	6	TEECKNMLDNNNNNNY	2733	0.91
PF3D7_1444100	6	SRDVEDKSVYDKKNES	2688	0.91
PF3D7_1444100	6	CNIKDTSDDMKNIDNI	2377	0.91
PF3D7_1444100	6	EVIIDDTPIYMKSEKN	169	0.91

Protein ID	Rank	Sequence	Start Position	Score
PF3D7_1444100	6	FCHGYTTNEFILKYLK	1683	0.91
PF3D7_1444100	6	KDKMEDINLMNKKDNT	1587	0.91
PF3D7_1444100	6	YPKSHANYEYDLEVHS	131	0.91
PF3D7_1444100	7	GKKIQLHNTGDMNAQF	6542	0.90
PF3D7_1444100	7	YSESSHCNKIRNNKNN	5734	0.90
PF3D7_1444100	7	NNTITKYITINAKSNK	5340	0.90
PF3D7_1444100	7	ELSITPNNYMDDILST	5209	0.90
PF3D7_1444100	7	SQHEISINKKNIKLNN	5138	0.90
PF3D7_1444100	7	KLKYIESNESQNETNP	4881	0.90
PF3D7_1444100	7	KDIIYNHRCWDRTNNM	4834	0.90
PF3D7_1444100	7	DIKCEYDYMFYNNANI	3561	0.90
PF3D7_1444100	7	NYKKNLYNSYDKNTNT	3503	0.90
PF3D7_1444100	7	YKVIKYQVYEYKYDND	297	0.90
PF3D7_1444100	7	DSQIIKYNKKINKIND	2830	0.90
PF3D7_1444100	7	DKEIIAHDIYINKSKE	267	0.90
PF3D7_1444100	7	KKNQTKYNYFHEYLNI	2148	0.90
PF3D7_1444100	7	CDIIIKPLLYNSGHSY	1930	0.90
PF3D7_1444100	7	PFYILNPSEFIRNKEK	19	0.90
PF3D7_1444100	7	EYSITIKNVSNKIVTL	1725	0.90
PF3D7_1444100	7	FIEIIYNIKFPSLLYT	1392	0.90
PF3D7_1447800	1	KGRIIMYPSSDNERKE	52	0.96
PF3D7_1447800	2	DCFGRRPIHFFKHLNK	560	0.95
PF3D7_1447800	3	YNMCTYKYNSNNNNNN	516	0.94
PF3D7_1447800	3	KEEIKYDKKYDQSDTV	364	0.94
PF3D7_1447800	3	TIHKEDQGKGNNLLND	1539	0.94
PF3D7_1447800	3	YKCEDDKGVSNKVEYT	1157	0.94
PF3D7_1447800	4	NNKEIKKCDMNNNVNN	750	0.93
PF3D7_1447800	5	QIIIGEQNEREKYGTD	727	0.92
PF3D7_1447800	5	LFEYIWPKVMKKYKGR	39	0.92
PF3D7_1447800	5	THKCYNDNENTNYKNI	1307	0.92
PF3D7_1447800	6	EHTPNNDILYNNISNE	853	0.91
PF3D7_1447800	6	HVIDDDKGENDDRGKN	169	0.91
PF3D7_1447800	6	NEVIHNENNMDNKETI	1137	0.91
PF3D7_1447800	7	DIYNEMYNQFNNILST	240	0.90
PF3D7_1447800	7	LFHFYSYDEPNNFITN	1642	0.90
PF3D7_1447800	7	KEIINHNNIYMENNNT	1519	0.90
PF3D7_1447800	7	KSEKKNEECYNKYMND	1387	0.90
PF3D7_1449000	1	EDKIEEYSQENKINVE	205	0.92
PF3D7_1449000	2	ILLEDDADIFDASAYE	82	0.91
PF3D7_1451600	1	IFSISPGHHHYVLKTP	641	0.91
PF3D7_1451600	2	HSCYTSSYESQMFDCN	558	0.90
PF3D7_1451600	2	FSGYPTAIHNDKYINF	230	0.90
PF3D7_1451900	1	YGGMNDDNSSNNNNNN	516	0.96
PF3D7_1451900	2	YGIIDGTKCADVVLCL	146	0.95
PF3D7_1451900	3	NDDISDDITDNITVNE	580	0.94
PF3D7_1451900	4	EQLQNYANGNNNNNNN	817	0.91
PF3D7_1451900	4	NDSTTTTNNSNNNNNN	430	0.91
PF3D7_1451900	5	MWTYISNEKHEANDDF	494	0.90
PF3D7_1451900	5	HNPIHITDVGDYYIDN	286	0.90
PF3D7_1451900	5	RGYMMADSCAYNSEND	255	0.90
PF3D7_1455800	1	HKSGSIDVAFHKECTS	287	0.97
PF3D7_1455800	2	RGSGYWCSEGKHNVND	62	0.96
PF3D7_1455800	2	KSCIESPNEELKKSCE	1555	0.96

Protein ID	Rank	Sequence	Start Position	Score
PF3D7_1455800	3	TSTKELGQTSDNPADN	544	0.95
PF3D7_1455800	4	PKCPKDDITSNINNNN	833	0.94
PF3D7_1455800	4	HPFDFKPYTPTPTLDT	1408	0.94
PF3D7_1455800	4	KSIISGYKEHIWNRVT	1340	0.94
PF3D7_1455800	5	CDMKMGATYYIPSVDN	590	0.93
PF3D7_1455800	5	NKPIYELPKPDIIIND	1156	0.93
PF3D7_1455800	6	KQVGSTDTTFLALINK	903	0.92
PF3D7_1455800	6	INSINLPEPFVNLDCQ	714	0.92
PF3D7_1455800	6	LDTAPDSVHFDVNLGS	332	0.92
PF3D7_1455800	7	TFFDSSSSEMSAFVCS	696	0.91
PF3D7_1455800	7	VDIINYCNQYDKENDN	1493	0.91
PF3D7_1455800	7	HSFEDSTRQMKKDAYY	1470	0.91
PF3D7_1455800	8	DSPIDKHSFEDSTRQM	1464	0.90
PF3D7_1465800	1	SDLSQSTTTPNNNINI	2899	0.96
PF3D7_1465800	1	QMTYDIDLEYKNKCNL	2855	0.96
PF3D7_1465800	2	VNLIDEKNRWEKYVNN	4878	0.95
PF3D7_1465800	2	NMENIDDDDDNNNNNN	4111	0.95
PF3D7_1465800	2	KFYIMYNYLFPKRKTF	3873	0.95
PF3D7_1465800	2	VKDIHDTETVDKNLNI	1387	0.95
PF3D7_1465800	3	CVTNDDRNDDDEGENN	4536	0.94
PF3D7_1465800	3	KEDISSYYTFISKLYN	3584	0.94
PF3D7_1465800	3	DEKGNIPNRFSGEDNN	2167	0.94
PF3D7_1465800	3	YQKNLPPYYNNNNNNN	1751	0.94
PF3D7_1465800	3	AVCSIEFVPSEKRTTP	1708	0.94
PF3D7_1465800	4	YGSKMDDYFDKKLLNV	6095	0.93
PF3D7_1465800	4	EDKVEDPNEEDYRFDV	5520	0.93
PF3D7_1465800	4	LKTIRKSNADRNGEKK	5408	0.93
PF3D7_1465800	4	DELIKEYIKKKKNLYS	5080	0.93
PF3D7_1465800	4	IMEIQGTYEKDEDNKD	4164	0.93
PF3D7_1465800	4	KLVNKNDNDFNNMNNP	4032	0.93
PF3D7_1465800	4	YCEIIKNNTMKDKNDL	2838	0.93
PF3D7_1465800	5	LSKYITKIFFNKGVKE	6065	0.92
PF3D7_1465800	5	TYSITKYKSNNKTLSS	5287	0.92
PF3D7_1465800	5	YNMNKTKNRFGNMHNN	3426	0.92
PF3D7_1465800	5	EWLIDSYLKSNKSNIE	3117	0.92
PF3D7_1465800	5	SSFETFCNEYDPDNNT	2505	0.92
PF3D7_1465800	5	TSKAEYSNDPDMINKN	2300	0.92
PF3D7_1465800	5	NNTITTNLNDNNMDNL	2280	0.92
PF3D7_1465800	5	KSILSSSNEMKPHLNN	1597	0.92
PF3D7_1465800	5	LMIKYWYTTFDFIKNV	1291	0.92
PF3D7_1465800	6	PREIYVDELMKKQYNP	6221	0.91
PF3D7_1465800	6	IKSIREWRRWFNYLQV	5697	0.91
PF3D7_1465800	6	NVEEDEEDEEDEEDDE	5454	0.91
PF3D7_1465800	6	YVESYSKYFNNFLDNP	540	0.91
PF3D7_1465800	6	NKGISDKEEQPNKLNT	4227	0.91
PF3D7_1465800	6	SIHHIDYIQNDNMKCN	4212	0.91
PF3D7_1465800	6	KVKTDDDGYDDDKSNS	2490	0.91
PF3D7_1465800	6	SEKRTTPYQKNNSIVT	1717	0.91
PF3D7_1465800	7	LYICEEKNVMDIKDNY	6441	0.90
PF3D7_1465800	7	EVKTIASKKNNSLVNT	629	0.90
PF3D7_1465800	7	VKEIHWPYYYFYICDI	6078	0.90
PF3D7_1465800	7	PFEYIYKYALDNNQKL	5790	0.90
PF3D7_1465800	7	ENNYEDYSEFDNMNDN	5638	0.90

Protein ID	Rank	Sequence	Start Position	Score
PF3D7_1465800	7	LCMWVHALKMYAEVYR	4790	0.90
PF3D7_1465800	7	NQFIDYNIYFNKDSDS	3700	0.90
PF3D7_1465800	7	NIIISICQLYDMLCNL	3166	0.90
PF3D7_1465800	7	SKTNNNMNTFDIFSNN	2578	0.90
PF3D7_1465800	7	VKLKEYSSKTNNNMNT	2571	0.90
PF3D7_1465800	7	YRKGGSKNEIDNDVTN	2026	0.90
PF3D7_1465800	7	KGSISLATKKNYQKNL	1740	0.90
PF3D7_1465800	7	NINYDKKEYYNVRNNT	1356	0.90
PF3D7_1465800	7	KKAIFYKDIPIYINNN	1219	0.90
PF3D7_1468100	1	HGKIYAYIGAVVGHDI	522	0.97
PF3D7_1468100	2	VHEIRTRTRVKNEKNL	2160	0.95
PF3D7_1468100	3	YNDYQYENEYSNKRNT	1382	0.94
PF3D7_1468100	4	IKKNIESYKMNNIKNI	777	0.93
PF3D7_1468100	4	HQHIQNQNASNQQINQ	460	0.93
PF3D7_1468100	4	NMKKEHPNEKDDDNNN	1730	0.93
PF3D7_1468100	4	ENKVGSHNAYDNDQNN	1456	0.93
PF3D7_1468100	4	PLRIQEYDTSRERIST	1246	0.93
PF3D7_1468100	4	GEWYRENYEGDIGCRS	1105	0.93
PF3D7_1468100	5	HDLISAATRNFNIQCQ	746	0.92
PF3D7_1468100	5	NGHINNSHLSNKKGNK	271	0.92
PF3D7_1468100	5	DGSIENNEEADKEAKD	2107	0.92
PF3D7_1468100	5	DKDASTKDENMKKEHP	1721	0.92
PF3D7_1468100	6	SQKNNTYNLHNHLQNE	409	0.91
PF3D7_1468100	6	GSFIMMGFLNDNNSPS	2251	0.91
PF3D7_1468100	6	EEEEDDDDEEDDDDDD	1992	0.91
PF3D7_1468100	6	STSNRDKSDADNMNND	1931	0.91
PF3D7_1468100	6	RADIKRKGNFDEENVK	1673	0.91
PF3D7_1468100	6	NDGTSYNNGMDKTQND	1043	0.91
PF3D7_1468100	7	METRKWTRRSVKGKIP	904	0.90
PF3D7_1468100	7	KSSNEEENEGTPADNE	2461	0.90
PF3D7_1468100	7	KNESDNDDENDYKITN	2398	0.90
PF3D7_1468100	7	QNENQDDNENEEKPNE	2081	0.90
PF3D7_1468100	7	CFFESKNVLGRANRNP	1202	0.90
PF3D7_1468100	7	YCEISKTHKERTLLDD	1011	0.90
PF3D7_1469600	1	GQEIEISAEYDKNNNK	1248	0.95
PF3D7_1469600	2	KEYIIYHNNNNNNNNN	372	0.94
PF3D7_1469600	2	HSYYMDYNNDNIYWNH	1795	0.94
PF3D7_1469600	3	AGTIEYLYDQINKKYF	796	0.93
PF3D7_1469600	3	SQIINMPNDHFNIITS	448	0.93
PF3D7_1469600	3	GSSTHSRYVMDNKEYI	360	0.93
PF3D7_1469600	3	QKLIQAPCVFFPDNSF	3065	0.93
PF3D7_1469600	3	DMEESNNYSNNFYLNS	218	0.93
PF3D7_1469600	3	YELIDNRTIFPNILDD	1399	0.93
PF3D7_1469600	3	DGDIKTGTKYLAKILE	1107	0.93
PF3D7_1469600	4	DTTIIALNEKMARCEN	3197	0.92
PF3D7_1469600	4	YDHIDDSNIIDLIKGT	2990	0.92
PF3D7_1469600	4	GQMCDDRMCDDQMCDD	1495	0.92
PF3D7_1469600	4	KDEIYHGQMCDDRMCD	1489	0.92
PF3D7_1469600	5	GKKILHSIFRNNNNNN	77	0.91
PF3D7_1469600	5	NVDAVWPGWGHSSENP	583	0.91
PF3D7_1469600	5	FKTAQSIEDFNKENLP	3080	0.91
PF3D7_1469600	5	TDEYEEGRDVIFIIND	2610	0.91
PF3D7_1469600	5	DNTISEPIFNNNSSDE	1733	0.91

Protein ID	Rank	Sequence	Start Position	Score
PF3D7_1469600	6	HIEIQVVGDMYGNVCS	733	0.90
PF3D7_1469600	6	GKLGSIPVGFIAVNKN	3033	0.90
PF3D7_1469600	6	ETIKEAYKVFMIKYNE	2336	0.90
PF3D7_1469600	6	TKIFFGIYKNNKNDNN	2227	0.90
PF3D7_1469600	6	TIDINKILESDIKINN	2103	0.90
PF3D7_1469600	6	DQMCDDQMCDDENVVK	1505	0.90
PF3D7_1475500	1	DVSIYSNSTLDDDHNP	303	0.96
PF3D7_1475500	2	RGTGCLGVVFRAKDDF	1107	0.95
PF3D7_1475500	3	KVSWAYSPEFVKISVS	89	0.93
PF3D7_1475500	4	KYFVEYITEFDKDLTN	457	0.92
PF3D7_1475500	4	MSEESGSSAYDAKRAI	39	0.92
PF3D7_1475500	5	IESGMDHYVGSINNNI	825	0.91
PF3D7_1475500	5	GILPGDSYNFPANDCA	549	0.91
PF3D7_1475500	5	ISMIKTHPKHGELGDN	408	0.91
PF3D7_1475500	5	KIYWEYPPLHYNISVS	359	0.91
PF3D7_1475500	6	CETTGMEDSFHSIVNT	756	0.90
PF3D7_1475500	6	KHANIDIYEVDKDNNM	1171	0.90
PF3D7_1477500	1	TEDYDDYEDYEEDLYP	417	0.96
PF3D7_1477500	2	TLKGIYINKYDPEINR	174	0.91
PF3D7_1477500	3	TEVQRSYTFSRNLSDN	77	0.90
PF3D7_1477500	3	YFSELYTDLRNSHKSP	225	0.90
PF3D7_1337500	1	MEEYSSSQLNDENQCE	622	0.95
PF3D7_1337500	1	TQHINEKNVPINTFNN	1438	0.95
PF3D7_1337500	2	KYVILYSNTNIKKENT	347	0.94
PF3D7_1337500	2	NGHNNHHGDSDHNADN	1886	0.94
PF3D7_1337500	2	HDMINYNNDMNNYQHD	1756	0.94
PF3D7_1337500	2	KERFGTTPLYNHGYNN	1617	0.94
PF3D7_1337500	3	KFMDTANNEKNKNNNI	675	0.93
PF3D7_1337500	3	NNITETTNTFINEKNQ	3158	0.93
PF3D7_1337500	3	FMKIEGDNIFDNSTSN	1986	0.93
PF3D7_1337500	3	DESTDDNDENDKEEDE	1927	0.93
PF3D7_1337500	4	KREINEINSYQNGNVT	367	0.92
PF3D7_1337500	4	HKVINKDESNENNMNN	2798	0.92
PF3D7_1337500	4	YKTSDGRNKERFGTTP	1609	0.92
PF3D7_1337500	4	SKIICSDQHHVERINN	1498	0.92
PF3D7_1337500	4	YHEIVTRCCRNNTCTC	1351	0.92
PF3D7_1337500	4	YHPQNDSNYYKEIRNT	1093	0.92
PF3D7_1337500	5	NYSIENDNVYDENYNI	3025	0.91
PF3D7_1337500	5	SRSISMYEDINIPHNV	3006	0.91
PF3D7_1337500	5	TFDISTKNKDKKRKID	2596	0.91
PF3D7_1337500	5	DNDCDDNDCDDNDCDD	205	0.91
PF3D7_1337500	5	DNDCDDNDCDDNDCDD	195	0.91
PF3D7_1337500	5	DEENEEKDEENEEEND	1945	0.91
PF3D7_1337500	5	DNDCDDNDCDDNDCDD	185	0.91
PF3D7_1337500	5	DNDCDDNDCDDNDCDD	175	0.91
PF3D7_1337500	5	DEHENDNDDDDNDCDD	165	0.91
PF3D7_1337500	5	GNFIYNMRDHENIQDE	1581	0.91
PF3D7_1337500	5	YSNICALNNGQNSCKP	1550	0.91
PF3D7_1337500	5	EYVICEPTKSTQHINE	1428	0.91
PF3D7_1337500	5	YRNISSSNHHNINNIP	1176	0.91
PF3D7_1337500	6	SREINDQGHDKNKCSH	991	0.90
PF3D7_1337500	6	CISIDNKINMKNKSIP	646	0.90
PF3D7_1337500	6	TKTDLNHSEYDKGIKD	515	0.90

Protein ID	Rank	Sequence	Start Position	Score
PF3D7_1337500	6	NINKKTKEVYNNECNN	274	0.90
PF3D7_1337500	6	MVTINNMNSINNMNNI	2172	0.90
PF3D7_1337500	6	HGDSDHNADNNYNDNN	1892	0.90
PF3D7_1337500	6	HHMVNYKNDMDNYNHH	1686	0.90
PF3D7_1337500	6	HNYINKDINYNNEKNE	1654	0.90
PF3D7_1337500	6	PITQKDSLSNDLLRNN	1072	0.90
PF3D7_0110600	1	DGDDDEDGDDDNDDND	931	0.95
PF3D7_0110600	2	DEEEGEDGEDDEEEND	909	0.94
PF3D7_0110600	3	SKEYERKVTYHKECCS	799	0.92
PF3D7_0110600	3	HDDIKKEYDHKVTKNN	734	0.92
PF3D7_0110600	3	LMSIEDYLKKEKNRDY	1144	0.92
PF3D7_0110600	4	KSLLKNYNYHIKKKNK	707	0.91
PF3D7_0110600	4	SSTELIPNECDHKPNE	490	0.91
PF3D7_0110600	4	DGKNISCIHPNAYAKR	1685	0.91
PF3D7_0110600	5	YFIIDDNNIFDNGPII	667	0.90
PF3D7_0110600	5	SSYIYNKNIFCSKYNT	611	0.90
PF3D7_0110600	5	TSDVLTPIFFNNGDEK	510	0.90
PF3D7_0110600	5	LGTGDEIITSQIKYTF	307	0.90
PF3D7_0110600	5	TISQKYTSSYDSLDDS	1256	0.90
PF3D7_0206800	1	KTTTTTTTTNDAEAST	100	0.95
PF3D7_0206800	2	STSSENPNHKNAETNP	116	0.94
PF3D7_0206800	3	GQHGHMHGSRNNHPQN	206	0.93
PF3D7_0206800	3	KGEVQEPNQANKETQN	134	0.93
PF3D7_0206800	4	QKECTDGNKENCGAAT	226	0.90
PF3D7_0207600	1	YGETKDTTENNKVDVR	280	0.95
PF3D7_0207600	2	RKYIDTQDVNKKHSCT	934	0.94
PF3D7_0207600	3	TGTVRGDTEPISDSSS	194	0.93
PF3D7_0207600	4	FVKIIKTEVMNKGSVI	718	0.92
PF3D7_0207600	4	CYKGEHKDRCDEGSSP	627	0.92
PF3D7_0207600	5	LFSEKEDNENNKKLGN	864	0.91
PF3D7_0207600	6	CEEKTSPGLCLSKLDT	973	0.90
PF3D7_0207600	6	KHSCTRSYAFNPENYE	945	0.90
PF3D7_0207600	6	SWGPYWGDEGYFKVDM	788	0.90
PF3D7_0207600	6	SSEPSNPVSSGHSVST	78	0.90
PF3D7_0207600	6	GSTGASQPGSSEPSNP	69	0.90
PF3D7_0207600	6	PELNYDLEYFNEHLYN	514	0.90
PF3D7_0207600	6	TGESQTGNTGGGQAGN	23	0.90
PF3D7_0420000	1	QESIKMKNEKDNEKNN	636	0.98
PF3D7_0420000	2	NKTIMSDTDMNSKDNV	470	0.96
PF3D7_0420000	2	KESINNHIIYDLKQTP	341	0.96
PF3D7_0420000	3	HIIYDLKQTPNTHQNN	347	0.95
PF3D7_0420000	3	HDHIYTSNTYKNNKYD	2997	0.95
PF3D7_0420000	3	DEEKYMKQRYDSKCDG	2882	0.95
PF3D7_0420000	3	HTEENYKPPSINNNNN	2692	0.95
PF3D7_0420000	3	HEEYDENYTYDENNKA	1799	0.95
PF3D7_0420000	3	NTCIDTYNTDDHKIMD	1355	0.95
PF3D7_0420000	4	DNTIDTNNVPINNTVP	772	0.94
PF3D7_0420000	4	APEIDDNECENLPEQP	408	0.94
PF3D7_0420000	4	KRYIDTPPEKKKVATK	2279	0.94
PF3D7_0420000	4	RRRYYSCSRFDKGIYN	1221	0.94
PF3D7_0420000	5	KKKNKDTYENMKYKNI	324	0.93
PF3D7_0420000	5	RSFILGDNTYKNPRNS	3117	0.93
PF3D7_0420000	5	SSRIINSSKFNTNDCN	2309	0.93

Protein ID	Rank	Sequence	Start Position	Score
PF3D7_0420000	5	HNEIESNNIYLNYANN	2193	0.93
PF3D7_0420000	5	AHNITNRNNSNNNNNI	183	0.93
PF3D7_0420000	5	GKYIFHPNINNINNNN	1108	0.93
PF3D7_0420000	6	YDYKYNHETNNKYQGP	3344	0.92
PF3D7_0420000	6	HDDKYDDKYYDKYDDK	3224	0.92
PF3D7_0420000	6	DYHIYNNDRYVDRYDD	3183	0.92
PF3D7_0420000	6	DEEKYSHYENNNIHDS	3135	0.92
PF3D7_0420000	6	PHIIKDKNKKRTLKNN	2563	0.92
PF3D7_0420000	6	KEKHIYPDEKNNKKNI	1879	0.92
PF3D7_0420000	6	KITDKNTISFNNNNNN	151	0.92
PF3D7_0420000	7	HAVEKSKNDCKHRVNK	907	0.91
PF3D7_0420000	7	VEYNTNSLKYNNNGND	659	0.91
PF3D7_0420000	7	YEREHKYVHHHNNYNP	3263	0.91
PF3D7_0420000	7	YERRKTSIFSNKYEKP	3155	0.91
PF3D7_0420000	7	YEKYDKYEKYDKYEKY	3084	0.91
PF3D7_0420000	7	YHYEKNYREEKNRRND	3016	0.91
PF3D7_0420000	7	HKDIQDEYYYEKRKMS	2827	0.91
PF3D7_0420000	7	HADASKNNDNNSELNN	2778	0.91
PF3D7_0420000	7	FCHIKELLTHNKDNKQ	258	0.91
PF3D7_0420000	7	KLSMDDYVKIRKYLNF	1730	0.91
PF3D7_0420000	7	GGDDEYPINFNHDKNE	1620	0.91
PF3D7_0420000	7	KMFILNDEMFNNINNN	1586	0.91
PF3D7_0420000	7	DHHIDEENMERNYLSD	1238	0.91
PF3D7_0420000	7	PKVWYCKLNNDVRYNS	119	0.91
PF3D7_0420000	8	HMDQMKNVKRKKSQTN	617	0.90
PF3D7_0420000	8	TGKEDEQKNEDENENT	545	0.90
PF3D7_0420000	8	QGLYKNDSLGKKTKND	385	0.90
PF3D7_0420000	8	KVDIQNKNEYINNNIK	3306	0.90
PF3D7_0420000	8	YDKYDKYDKYDKYEKY	3069	0.90
PF3D7_0420000	8	LKKRKTYQEEDGKKND	2619	0.90
PF3D7_0420000	8	YPDIIKKVEQNIGVSK	2380	0.90
PF3D7_0420000	8	HSEVDDNINEDDKNQT	1284	0.90
PF3D7_0420000	8	EVTYDDKNVMNNDNEM	1043	0.90
PF3D7_0423600	1	KSSILTTGTSNNNNNV	1093	0.97
PF3D7_0423600	2	QICVKDDNELDNNNNK	985	0.96
PF3D7_0423600	2	EHHINMEDEKNEKLND	1549	0.96
PF3D7_0423600	3	EKLIDKLDDYKKKINI	1451	0.94
PF3D7_0423600	4	KEHIQKNITKNITKNE	244	0.92
PF3D7_0423600	5	NVKNMNQTKDDNGCTK	411	0.91
PF3D7_0423600	5	EEAEEEEEEEDKFLTP	1587	0.91
PF3D7_0423600	6	YKKIKTIIEHEIELNS	642	0.90
PF3D7_0505000	1	MKKINDNNKNDNNKNN	955	0.94
PF3D7_0505000	1	EELIEEYICPEKVMFT	5	0.94
PF3D7_0505000	1	KNDIIHKINYDVIRNA	2139	0.94
PF3D7_0505000	2	SYDYSSHNSSDNEDNN	833	0.93
PF3D7_0505000	2	TSHIYNTLFPNVYNID	2488	0.93
PF3D7_0505000	2	KEIIHDKTKYDLYLTH	2308	0.93
PF3D7_0505000	2	DGDEDEDNDGDEDEDN	1980	0.93
PF3D7_0505000	3	GHREETYKEYEKKSHS	68	0.92
PF3D7_0505000	3	YKSIIMKNVMKMLVSV	2900	0.92
PF3D7_0505000	3	YGDKYGDKYDDKYDNK	2630	0.92
PF3D7_0505000	3	DGDEDNGGDEDNDGND	2010	0.92
PF3D7_0505000	3	YEHVLKRIYYDNEITE	190	0.92

Protein ID	Rank	Sequence	Start Position	Score
PF3D7_0505000	4	YAEPSDYNSSDEEDYI	360	0.91
PF3D7_0505000	4	YETIQDINWYLSIASN	2942	0.91
PF3D7_0505000	4	KTKYDLYLTHDYYSDK	2314	0.91
PF3D7_0505000	4	IPENDGNNDKDKNLNM	1710	0.91
PF3D7_0505000	4	KEMEDEKNKMRKNKRN	1636	0.91
PF3D7_0505000	4	EEHMDSYIELLKKLCP	1243	0.91
PF3D7_0505000	5	TRDYKKKNRKRKGKKE	2783	0.90
PF3D7_0505000	5	DVTIGDNNNNNNNDGN	237	0.90
PF3D7_0505000	5	TNKIIDEKTEDCNKKI	2349	0.90
PF3D7_0505000	5	HDYYSDKIDYKKNCYT	2323	0.90
PF3D7_0505000	5	VTHGDEDNDGDEDEDN	1972	0.90
PF3D7_0505000	5	CIINLDPVKGDIYCDT	1834	0.90
PF3D7_0505000	5	EKDIKKPDFEEIKDNN	1500	0.90
PF3D7_0725100	1	YEHISFRNLRNNLRDP	333	0.96
PF3D7_0725100	1	EKEIYYYVSINNKKNF	1368	0.96
PF3D7_0725100	2	MNKIEGYTKCMFHRNN	661	0.94
PF3D7_0725100	3	VHMIMSNHIFDNFMNT	800	0.93
PF3D7_0725100	3	NKSGTDEKEHNNDSNN	552	0.93
PF3D7_0725100	4	HNHINNHNHINNHNNN	912	0.92
PF3D7_0725100	4	KGIKITPSEIKNKSKK	214	0.92
PF3D7_0725100	5	NSSACDDKTFHYNNNK	593	0.91
PF3D7_0725100	5	CFYMCPPLLYDTNKQT	1227	0.91
PF3D7_0725100	5	KIMGVNYIKHMEMSNK	1047	0.91
PF3D7_0725100	6	LKEKQRGVLERKDDNN	839	0.90
PF3D7_0725100	6	EYLYIEKNEKDKNKDD	725	0.90
PF3D7_0725100	6	TKMEIYKKLFNCLYNF	363	0.90
PF3D7_0728600	1	SSSYMNHNTFYSLNNS	1106	0.96
PF3D7_0728600	2	LFCIKNLYHNNNNNNN	1063	0.94
PF3D7_0728600	3	SKKSDDTNEGKNKIDE	1672	0.93
PF3D7_0728600	3	KEDYLKKNTMNALLNT	1306	0.93
PF3D7_0728600	4	SKQINNTNSYDYNMNS	324	0.92
PF3D7_0728600	4	DDDGDDDDDGDDDDDD	1428	0.92
PF3D7_0728600	5	DDDNDDDNDDDYNDDD	1498	0.91
PF3D7_0728600	5	DDDDDDYNDDDNDDDN	1482	0.91
PF3D7_0728600	5	EFSSYSSGEENFKKNM	1280	0.91
PF3D7_0728600	6	IKCIIPGNNMYEHNNS	363	0.90
PF3D7_0728600	6	CGHAICKNCVDKIHAQ	2122	0.90
PF3D7_0728600	6	KGRKGTDKKPNNDKKK	1954	0.90
PF3D7_0728600	6	DDDGDDDDDDDDYNDD	1476	0.90
PF3D7_0728600	6	EDDIKDKGGRNKKGGT	1405	0.90
PF3D7_0728600	6	ILPQEKKNEERNKCDD	1195	0.90
PF3D7_0728600	6	EKKDVTTLSMEKKCTP	1169	0.90
PF3D7_0728600	6	KILNRTSQFYDHEDTK	1008	0.90
PF3D7_0801000	1	SCDIIKPSKFNKKNLF	480	0.96
PF3D7_0801000	2	YASEQEYFDRGEQLND	305	0.94
PF3D7_0801000	3	GRGSRYPEEERYNYNN	946	0.93
PF3D7_0801000	4	DSSSTTTSTGTKLQNV	217	0.92
PF3D7_0801000	5	SHNIINRRSRNLGANP	81	0.91
PF3D7_0801000	5	NQHVNDRRNFDERNQN	694	0.91
PF3D7_0801000	5	DRTNIKKGLNEQHVNP	430	0.91
PF3D7_0801000	5	KDEPVDDNTSNKLKDE	358	0.91
PF3D7_0801000	5	KNIWMERLTYKMRKYS	1202	0.91
PF3D7_0801000	6	NFSGSFKCLFKNKRNK	22	0.90

Protein ID	Rank	Sequence	Start Position	Score
PF3D7_0801000	6	PELELYTNDQNSRNNT	136	0.90
PF3D7_0815300	1	LKCIYENSVINNEVND	160	0.94
PF3D7_0815300	2	LGILYNYYFFNIKVNK	126	0.93
PF3D7_0815300	3	DKSIEKIPKAHYYNNQ	37	0.92
PF3D7_0815300	4	CSSYNFQNNYDKEENK	895	0.91
PF3D7_0815300	4	DSAHKLPPSGGFGLNL	513	0.91
PF3D7_0815300	4	HVQINDYVLFKKENGE	1053	0.91
PF3D7_0815300	5	HIPYLTKREMLLYSNP	449	0.90
PF3D7_0815300	5	IGGGPTGITSGLYLQK	11	0.90
PF3D7_1021700	1	HNEHNHHNEHDNNNNK	667	0.97
PF3D7_1021700	1	TEHIKSDYVNIKGDCI	4492	0.97
PF3D7_1021700	1	KMEIEETTEFRYFNTN	1907	0.97
PF3D7_1021700	2	KGHIYMDDDMYIDVDV	6271	0.96
PF3D7_1021700	2	TETSDYYIEHNENKKN	5116	0.96
PF3D7_1021700	2	LTMITDHNDMQQNMDN	1046	0.96
PF3D7_1021700	3	HFKISYDEEKKKRKKK	6307	0.95
PF3D7_1021700	3	DKDINDYTTKYNRKNN	5994	0.95
PF3D7_1021700	3	HPKIDDDNEKNNHENN	379	0.95
PF3D7_1021700	3	KYSIYDNNKYHIYDNN	3522	0.95
PF3D7_1021700	3	SFNITHCEEENANKNK	1647	0.95
PF3D7_1021700	4	MIYIQHDYTDNIPADD	4429	0.94
PF3D7_1021700	4	HQSYRTHQSYKRHANN	2019	0.94
PF3D7_1021700	4	EEENNDHTDNNNKNNN	1551	0.94
PF3D7_1021700	5	KHVEEYSLDNNKRKKS	623	0.93
PF3D7_1021700	5	KDYYYYYYSSMNILND	5727	0.93
PF3D7_1021700	5	MEYIDYYNVDIKINNI	5405	0.93
PF3D7_1021700	5	TILIDDLTLFNKNNKH	4453	0.93
PF3D7_1021700	5	KREIEELEEHREKETI	4362	0.93
PF3D7_1021700	5	IQLYKYANTPDKIHQI	3816	0.93
PF3D7_1021700	5	KKEEIEYNYNNKDNCE	3611	0.93
PF3D7_1021700	5	KYHIYDNNESNNKKDD	3530	0.93
PF3D7_1021700	5	KEMEEAKTYNNNNNKN	3399	0.93
PF3D7_1021700	5	ISKEQMYSEKNKNKNN	2530	0.93
PF3D7_1021700	5	SSVGFTNEDPTKRRNK	1087	0.93
PF3D7_1021700	6	YIYYNDYYYNNNYMYR	6049	0.92
PF3D7_1021700	6	SMNILNDPDNNKRCDI	5736	0.92
PF3D7_1021700	6	SGSSRTSYTNKKKGYK	5539	0.92
PF3D7_1021700	6	YKIINDNYINNNENNN	5312	0.92
PF3D7_1021700	6	HMLQKNKQTNDNYLVK	4885	0.92
PF3D7_1021700	6	GGVGQNNTGDNNLLNN	449	0.92
PF3D7_1021700	6	KEMQKKKKDRHKKNNF	4389	0.92
PF3D7_1021700	6	TKTITINNRYTIINNT	4017	0.92
PF3D7_1021700	6	EEEISKQNEANDMLNY	3175	0.92
PF3D7_1021700	6	SCSYKSMNENDENVKS	2421	0.92
PF3D7_1021700	6	MQCRKYYQEFLKMNNL	1195	0.92
PF3D7_1021700	6	TEHIKRDNSIHSNDNC	1139	0.92
PF3D7_1021700	7	TLSKKNRYDDDKDIND	5984	0.91
PF3D7_1021700	7	ERTVQEKDEKKSEDCI	472	0.91
PF3D7_1021700	7	HKKYNDKKYNQKENNN	4167	0.91
PF3D7_1021700	7	YMTYSFSLENLQNQNN	4055	0.91
PF3D7_1021700	7	KHHIIHYNNNNIIKKK	3690	0.91
PF3D7_1021700	7	HDMIKNSVSKHILNND	3589	0.91
PF3D7_1021700	7	NYHNNYHNNNDKGANI	3070	0.91

Protein ID	Rank	Sequence	Start Position	Score
PF3D7_1021700	7	LPIIYELKKFNEKKKI	2903	0.91
PF3D7_1021700	7	LEKMEIYLTFENNRNK	2708	0.91
PF3D7_1021700	7	CIEQIDDIDYNKESLQ	2147	0.91
PF3D7_1021700	7	KTTIEHNNNFCEEQNK	1625	0.91
PF3D7_1021700	7	HAIIHNNSHYNNKKTN	1301	0.91
PF3D7_1021700	8	KKEKTTPNSIIFKLNF	6899	0.90
PF3D7_1021700	8	HVTGEENNSFTSSLYN	6705	0.90
PF3D7_1021700	8	FLDIKAPKKRNYFVYT	6623	0.90
PF3D7_1021700	8	TSLGMTKSKGYNDLFT	5149	0.90
PF3D7_1021700	8	EYHNEYHNENNGNINS	5009	0.90
PF3D7_1021700	8	CVNIKDNCVNIKGNCV	4541	0.90
PF3D7_1021700	8	SDHINDKNIINSYKNE	4409	0.90
PF3D7_1021700	8	HKRNYNHRNGGVGQNN	440	0.90
PF3D7_1021700	8	TCEISDKKYTEKGMTK	4138	0.90
PF3D7_1021700	8	YDFYIHKNENNNKCYF	3321	0.90
PF3D7_1021700	8	KNEIEENNYPDKYPMN	3114	0.90
PF3D7_1021700	8	VSIYFSSNLFINNSTN	2958	0.90
PF3D7_1021700	8	YEIIENEDDNSSSCNN	2495	0.90
PF3D7_1021700	8	CSSAYHNMKYEKYVDI	2258	0.90
PF3D7_1021700	8	HEKNIDGKTNIIYHNN	2163	0.90
PF3D7_1021700	8	EHIWDEQNNQNNKEND	2110	0.90
PF3D7_1021700	8	NNMVDGQIECNENVTE	1586	0.90
PF3D7_1021700	8	NEKIQNILSHKNKINK	1397	0.90
PF3D7_1024800	1	AKLIMTYNKNMNALYS	675	0.96
PF3D7_1024800	2	HSGYVVGLWRDFPRNT	633	0.94
PF3D7_1024800	2	TQEYIKDVENNVERNN	1333	0.94
PF3D7_1024800	3	LRCEDFPNISDRCVNN	927	0.93
PF3D7_1024800	3	IMVINGYNCNIYEKNT	590	0.93
PF3D7_1024800	3	GDLLSTSDGNDNENND	263	0.93
PF3D7_1024800	3	HSDSTHVPDEDNEAQD	1091	0.93
PF3D7_1024800	3	DIPEKDMSEYRYPLNV	1002	0.93
PF3D7_1024800	4	LKHKYCPVDDNNNDNN	849	0.92
PF3D7_1024800	4	QDSIKDELSRNNENDY	370	0.92
PF3D7_1024800	5	IDIIKKDDAVDNKENE	65	0.91
PF3D7_1024800	5	YFSPTTVGEYNKLVDT	454	0.91
PF3D7_1024800	5	RKEIQNEFSFRNGLYF	440	0.91
PF3D7_1024800	5	HNEEKDDDDDDNNNIE	108	0.91
PF3D7_1024800	5	AMKAYASGKMYNIVTP	1048	0.91
PF3D7_1024800	6	YFEENPKNEEDAHDED	133	0.90
PF3D7_1024800	6	PNDPFYDGTFNVLNND	1299	0.90
PF3D7_1025400	1	DHDIIDEDTSNNEDLE	1133	0.94
PF3D7_1025400	2	YMNGQTNNVMNGQGNN	913	0.92
PF3D7_1025400	2	YMNGQTNNVMNGQGNN	897	0.92
PF3D7_1025400	2	KGRIKYYDAYSILYNF	538	0.92
PF3D7_1025400	2	YKSIIEDNKGRIKYYD	530	0.92
PF3D7_1025400	3	YGNINAQGNYMNGQTN	888	0.91
PF3D7_1025400	3	SGLYDKDNYKRNEEDK	1203	0.91
PF3D7_1025400	3	QVHTEIPYRPNENAQT	1077	0.91
PF3D7_1025400	4	YMNIQTNNCMNGQENN	953	0.90
PF3D7_1243900	1	DSQISIPLRFNNKNDI	562	0.97
PF3D7_1243900	2	QKKIVAPVSFNTTMNF	679	0.95
PF3D7_1243900	3	TITLSHTPKGHEGLNI	377	0.94
PF3D7_1243900	3	PIKIDEIQVYDIIKNE	1638	0.94

Protein ID	Rank	Sequence	Start Position	Score
PF3D7_1243900	4	LKTIKSIPENCTDVNT	713	0.93
PF3D7_1243900	4	KGIVDYPYVMHELEAP	449	0.93
PF3D7_1243900	4	TEKEEGKNAEINENND	17	0.93
PF3D7_1243900	4	IQEISDYEYKNIKENK	1699	0.93
PF3D7_1243900	5	HFSIWTYPDILGNSTN	663	0.92
PF3D7_1243900	5	DETSRRTRLMKKTLSP	544	0.92
PF3D7_1243900	5	TALSDDSDERKNKNDR	321	0.92
PF3D7_1243900	6	KKHWGYPRRWKVILWD	84	0.91
PF3D7_1324600	1	KKEIYTHNSNNIYDNN	280	0.97
PF3D7_1324600	2	EHDIYTYEKSHPNKNN	593	0.95
PF3D7_1324600	3	AQLISSTFFFNNNHNT	609	0.94
PF3D7_1324600	4	GCMVINNNDNMKRNNN	936	0.93
PF3D7_1324600	4	YKIISDPIGEHINDDI	732	0.93
PF3D7_1324600	4	KKKIQMKGTYSHDNTF	231	0.93
PF3D7_1324600	4	MKKWFFSRGNNNNNNN	1369	0.93
PF3D7_1324600	4	HSIQIKCNKRMDGNNN	135	0.93
PF3D7_1324600	5	EGKEKIHNDFNKKNYI	804	0.92
PF3D7_1324600	5	TFDAGENKTFDAGENK	709	0.92
PF3D7_1324600	5	TFDAGENKTFDAGENK	701	0.92
PF3D7_1324600	5	TFDAGENKTFDAGENK	693	0.92
PF3D7_1324600	5	TFDAGENKTFDAGENK	685	0.92
PF3D7_1324600	5	TFDAGENKTFDAGENK	677	0.92
PF3D7_1324600	5	TFDAGENKTFDAGENK	669	0.92
PF3D7_1324600	5	KFDEGENKTFDAGENK	661	0.92
PF3D7_1324600	5	EEEKEERYSSDGKDNV	633	0.92
PF3D7_1324600	5	PFYISYMNYHLNLYNI	385	0.92
PF3D7_1324600	5	LLYQMDHRKYFNYLNK	173	0.92
PF3D7_1324600	5	YVTVMRQNDLTKNKNK	1554	0.92
PF3D7_1324600	6	CFDAQTKSTMPINLYS	891	0.91
PF3D7_1324600	7	NKREYYRETYYKHDNI	842	0.90
PF3D7_1326300	1	QWVAKTNQTNPHHLDP	216	0.93
PF3D7_1326300	2	DRERRSIREERSGRDD	136	0.92
PF3D7_1326300	2	DRERDRDRERDRDRDR	116	0.92
PF3D7_1326300	3	HSRRSEHKKEDVERHP	15	0.91
PF3D7_1326300	3	DRERDRDRDRDRDRDR	122	0.91
PF3D7_1326300	4	NNPYNKPPPPPPGAPP	382	0.90
PF3D7_1327300	1	KGTIKHTLEYLKRAAE	380	0.96
PF3D7_1327300	1	KGKHNDYITPRIGANI	1481	0.96
PF3D7_1327300	2	DDTITDDIVKDKKKLS	793	0.95
PF3D7_1327300	3	EKNIDDKENFGYKKDN	608	0.94
PF3D7_1327300	3	EKTIEDKNESELEKYE	566	0.94
PF3D7_1327300	4	EEDIQKDIEEDIQKDI	1159	0.93
PF3D7_1327300	5	NVENNNKYDNDNDNNN	1439	0.92
PF3D7_1327300	5	THVIIDKEKNDVDVPF	132	0.92
PF3D7_1327300	5	DSMNDTNNDYNNNDKF	1135	0.92
PF3D7_1327300	5	MNDIDNMNGMDNINDI	1119	0.92
PF3D7_1327300	6	VDVQMEQNKFNGSKNK	718	0.91
PF3D7_1327300	6	KEMYYDDMMRNVENNN	1429	0.91
PF3D7_1327300	6	SGTIISKGVLEKNKGI	1324	0.91
PF3D7_1327300	7	KSDENDKNYKNNENNN	647	0.90
PF3D7_1327300	7	IMSINDKVIEENIVNK	536	0.90
PF3D7_1327300	7	TLSKIPYSKYDVIRKP	281	0.90
PF3D7_1327300	7	KRSYSVPNKAVAKNIP	219	0.90

Protein ID	Rank	Sequence	Start Position	Score
PF3D7_1327300	7	LSAIHNREDMNKNDEE	1582	0.90
PF3D7_1349300	1	TEEVQYIETYMKDNDT	923	0.96
PF3D7_1349300	2	SKHKTSPARSINYLKP	815	0.93
PF3D7_1349300	2	KSVIIMSDNDNFIVQN	530	0.93
PF3D7_1349300	3	ESYISTPRSSHRYIFP	1448	0.92
PF3D7_1349300	3	SCCEISPYDEISKKTK	136	0.92
PF3D7_1349300	4	GSLEKYIYKDEKKKNS	1607	0.91
PF3D7_1349300	4	FLFIDDYLTSNENKFD	1281	0.91
PF3D7_1349300	4	LFCNSDKLFYDNFRNL	12	0.91
PF3D7_1349300	4	GDIIKTKRENNNILNG	1158	0.91
PF3D7_1349300	5	HQNIIKTVCSKNDNNI	981	0.90
PF3D7_1349300	5	DDCIKNKDTYKNEEKK	271	0.90
PF3D7_1349300	5	IGGGSYAQVFRAKYKT	1411	0.90
PF3D7_1349300	5	DSLNNDSLNNDSLNND	1199	0.90
PF3D7_0804500	1	TNVIQGINEKNAILDN	5503	0.96
PF3D7_0804500	1	THHIIPTNLNNNNNNN	5463	0.96
PF3D7_0804500	1	HSHEDEHNIYDKKKNG	4104	0.96
PF3D7_0804500	2	YGSITSMTWNDSGNLL	5069	0.95
PF3D7_0804500	2	KFHHQNGIYDDNNNNN	3410	0.95
PF3D7_0804500	3	NVRISRDSAHDEYDNK	9	0.94
PF3D7_0804500	3	NKNIIDPSYNYNNNNE	4148	0.94
PF3D7_0804500	3	NIMISFQPLFDVILCD	3426	0.94
PF3D7_0804500	3	NNEIYSDNDHHHNDDN	2263	0.94
PF3D7_0804500	4	FSPLSYYIQNNKHLNN	791	0.93
PF3D7_0804500	4	MKNCIYYNEFKSNHNN	632	0.93
PF3D7_0804500	4	KLHIMRIKRRRKKLNK	5287	0.93
PF3D7_0804500	4	ESEQDDYYDDDYYDDD	5179	0.93
PF3D7_0804500	4	ISHVDYGNEHDVYKNN	4804	0.93
PF3D7_0804500	4	YHPINKHVINSYNIFP	48	0.93
PF3D7_0804500	4	GEEEEEGEGNNQNRND	4658	0.93
PF3D7_0804500	4	DRHGEYKDDKTQNCTS	1468	0.93
PF3D7_0804500	4	CKMIESFDSFFLLLCI	1187	0.93
PF3D7_0804500	5	TQPLENSNTYENNMNK	978	0.92
PF3D7_0804500	5	EIEIKHDVSPKKCICE	5836	0.92
PF3D7_0804500	5	DDYYDDDYYDDNSYND	5198	0.92
PF3D7_0804500	5	DGYYNNIYKGNNNNNN	4410	0.92
PF3D7_0804500	5	KEGGTCTDVNRNKKNT	304	0.92
PF3D7_0804500	5	GIMREEQNKKNKEKNK	111	0.92
PF3D7_0804500	6	EYLIVYGSKNDLLDNK	602	0.91
PF3D7_0804500	6	HREIYCFKPFDFPIFK	5786	0.91
PF3D7_0804500	6	VWTSTFTNYMYKSVCP	5706	0.91
PF3D7_0804500	6	YTTKNNDTSNNNNNNN	5529	0.91
PF3D7_0804500	6	KGRKDDQETYEEEYDE	5163	0.91
PF3D7_0804500	6	SSGYEKKEHFNNLHTD	4890	0.91
PF3D7_0804500	6	YNDIEYDNKYISKKKK	4531	0.91
PF3D7_0804500	6	SSTCNNDSHFYMSNNP	3796	0.91
PF3D7_0804500	6	HFIVISFMLMDQGVYD	1745	0.91
PF3D7_0804500	6	NQTIYNTQRNNNFVLS	1055	0.91
PF3D7_0804500	7	YSYNRMPSDNNKSNNL	5903	0.90
PF3D7_0804500	7	EKERGQHYKGRKDDQE	5155	0.90
PF3D7_0804500	7	DAHIEPKNENIQNGKP	4602	0.90
PF3D7_0804500	7	DDYIDDYIDDYSDDYS	433	0.90
PF3D7_0804500	7	KSNVFIPNTMNIKKGD	3897	0.90

Protein ID	Rank	Sequence	Start Position	Score
PF3D7_0804500	7	KIKIYYYYTALNKYKI	3453	0.90
PF3D7_0804500	7	METSSTPQGFISNNIS	2781	0.90
PF3D7_0804500	7	NKDYDDDDDDDNSLLS	2147	0.90
PF3D7_0804500	7	EEAEKKNNKMNNNNNN	2125	0.90
PF3D7_0804500	7	SSYIQPFNVNKKNDTL	1233	0.90
PF3D7_0804500	7	IKMICIFDDYSSYIQP	1223	0.90
PF3D7_0804500	7	SSNINTNYVPHRNNNL	1030	0.90
PF3D7_0826100	1	NKMIHNHNNNNNNNNE	3538	0.95
PF3D7_0826100	1	KILTNSPDTLNNDLND	3322	0.95
PF3D7_0826100	2	HKEVQEYQDSDIYDDD	8109	0.94
PF3D7_0826100	2	KLSRFTTTTNDKKKNT	6191	0.94
PF3D7_0826100	2	DDITENRNLYDNRLNR	5859	0.94
PF3D7_0826100	2	YSKEDTGHVYMNNDNT	526	0.94
PF3D7_0826100	2	HARIYYYDNIMNPNNK	2953	0.94
PF3D7_0826100	2	NILKMEPNTNNKHNTN	1510	0.94
PF3D7_0826100	3	DRLPTAHTCFNQLDLP	8550	0.93
PF3D7_0826100	3	VSAIDNMNIFDYINNT	769	0.93
PF3D7_0826100	3	TLTGISEKAQDNIKNK	6388	0.93
PF3D7_0826100	3	YDEIDIEDEDAAERNS	5138	0.93
PF3D7_0826100	3	NSYHSDDNPFNAYQDN	3281	0.93
PF3D7_0826100	3	LKFIHHSTEVEPKLNE	2717	0.93
PF3D7_0826100	3	DYYIDDKNIHMHTNYP	1912	0.93
PF3D7_0826100	3	HKKNSYKYHMDNNKYL	1546	0.93
PF3D7_0826100	3	TNFIMYTQYVNFILNN	1475	0.93
PF3D7_0826100	3	YEHIYEDPTILSTLLK	1225	0.93
PF3D7_0826100	3	TGCTLEYNYNNYNDNN	105	0.93
PF3D7_0826100	4	DEEEDDDDDNDEYIQI	8142	0.92
PF3D7_0826100	4	KKKIQNNYTHDDSMNN	7970	0.92
PF3D7_0826100	4	APTLIDSITNKNDLNK	7578	0.92
PF3D7_0826100	4	TFLIHPSYFMPNDLDE	7145	0.92
PF3D7_0826100	4	DSDSDDYNDNSDNNNN	6273	0.92
PF3D7_0826100	4	SPSQEQYFTPRAYTQE	617	0.92
PF3D7_0826100	4	NSLIQASQHISNNNNY	593	0.92
PF3D7_0826100	4	DHHINSSLLDNNKNQN	5499	0.92
PF3D7_0826100	4	EDDIDDDDDEDDEEDV	5095	0.92
PF3D7_0826100	4	EHFIFDNGESDEEDDD	4917	0.92
PF3D7_0826100	4	GEKIKETNKNEENMND	4160	0.92
PF3D7_0826100	4	TVGTSNYNLSNNIKLP	3760	0.92
PF3D7_0826100	4	SRNIMQCREYNNNLND	2853	0.92
PF3D7_0826100	4	NGHIFNNNTNDIHNNN	2535	0.92
PF3D7_0826100	4	KKEDKYFNLYDYSYTP	2114	0.92
PF3D7_0826100	4	HVISEFHKYQNKNCKT	1749	0.92
PF3D7_0826100	4	KICIHEHVKRTIRNNQ	1659	0.92
PF3D7_0826100	4	NSTDDDDDDDDNIWNV	1099	0.92
PF3D7_0826100	5	EGVNTPVQENDHINNN	7610	0.91
PF3D7_0826100	5	NEEEVTSDDNNKNNNN	7339	0.91
PF3D7_0826100	5	GLPIGDSNNSSNYNNN	6884	0.91
PF3D7_0826100	5	YKNINYEELFDNILNI	6325	0.91
PF3D7_0826100	5	VSIIDLYYHFYGSNNR	5686	0.91
PF3D7_0826100	5	DVEEEDGDEEELHINN	5294	0.91
PF3D7_0826100	5	EDEEDDDGEDDEEDDD	5241	0.91
PF3D7_0826100	5	EVEEMDDDMENEDDDE	5080	0.91
PF3D7_0826100	5	DEDVEGDDDEDVEVDD	5044	0.91

Protein ID	Rank	Sequence	Start Position	Score
PF3D7_0826100	5	DYSNADTFTFNNRDNN	4676	0.91
PF3D7_0826100	5	KKTKEEKYSEDKNMFL	3710	0.91
PF3D7_0826100	5	SVHDINPSTNIHVNNN	3220	0.91
PF3D7_0826100	5	PLTINNNGSNDNNNND	2883	0.91
PF3D7_0826100	5	YIRKMSRDVQDNNLIK	2050	0.91
PF3D7_0826100	6	KKKLNEDQTNDNNNNN	8020	0.90
PF3D7_0826100	6	LDYIRHSQSNNNNNDN	796	0.90
PF3D7_0826100	6	KLAYDDSPFYINIYNK	7890	0.90
PF3D7_0826100	6	DKMIKNEKTDGEKKNN	7189	0.90
PF3D7_0826100	6	LWCASADLHAKKNNPS	7069	0.90
PF3D7_0826100	6	TSLINNNNNNNNNDNN	702	0.90
PF3D7_0826100	6	YICVEEPVPFQALIDI	7013	0.90
PF3D7_0826100	6	KYIIHNKLVMQKKNNK	70	0.90
PF3D7_0826100	6	ENDIIESNENDNITNA	6209	0.90
PF3D7_0826100	6	AGQKSSSNERDVLENN	5469	0.90
PF3D7_0826100	6	HVYMNNDNTNNKSHNN	533	0.90
PF3D7_0826100	6	RIREIDETEEDEEDDD	5232	0.90
PF3D7_0826100	6	DVEVDDDEEVDVDEDE	5054	0.90
PF3D7_0826100	6	LRSPYSLNTFKKKYPN	4520	0.90
PF3D7_0826100	6	NEENMNDIYNNNISNN	4169	0.90
PF3D7_0826100	6	IKICIDTLYFFKGYNS	3794	0.90
PF3D7_0826100	6	SNNIITATSPNITLNI	3173	0.90
PF3D7_0826100	6	DSCNNNIYHSNNKLDE	2904	0.90
PF3D7_0826100	6	MDSINNCNNMNVDMNN	2200	0.90
PF3D7_0826100	6	NVECYDDKTMFSIKDK	1113	0.90
PF3D7_1329000	1	YKMITDNNNNDNNNNN	1531	0.96
PF3D7_1329000	2	VKSILDDLRNDAGKTC	863	0.95
PF3D7_1329000	3	AQSIGEPGTQMTLKTF	1799	0.94
PF3D7_1329000	3	CNGIYEKETNNNELTT	1592	0.94
PF3D7_1329000	4	KAKIMDFKTFRFNECV	589	0.92
PF3D7_1329000	4	KEDIEVTDNEDEICDD	1977	0.92
PF3D7_1329000	5	NFEIQEREYSNKFGDL	725	0.91
PF3D7_1329000	5	PKIIAMRLTYPETVNK	381	0.91
PF3D7_1329000	5	GEHIIERLSYKMKEKN	2110	0.91
PF3D7_1329000	5	NVVQSKYNRMSKNLKK	1449	0.91
PF3D7_1329000	5	QMCAEEHITINNTNND	1287	0.91
PF3D7_1329000	6	KQLISVLIKPNKKENT	707	0.90
PF3D7_1329000	6	KKDINDDNNNNDDNNN	2054	0.90
PF3D7_0501100	1	YRKLAMKWHPDKHLND	100	0.92
PF3D7_0501100	2	DLFIEFDIVFPKKLSP	373	0.90
PF3D7_0529400	1	NEKYDSYDEYEKYKNF	239	0.96
PF3D7_0529400	2	RHDAYDGYRGDLYLNS	879	0.95
PF3D7_0529400	2	YIMRNEKNLYNKKVNF	813	0.95
PF3D7_0529400	2	MGNIIKYVVEDNNSNM	411	0.95
PF3D7_0529400	3	KQHIVTTNYYKHEDIP	1020	0.94
PF3D7_0529400	4	DEEYDNNNLYDNMKGD	791	0.93
PF3D7_0529400	4	NSDIKNNDTKNKNKNN	554	0.93
PF3D7_0529400	4	MEKIENKYNMMVNDTK	538	0.93
PF3D7_0529400	4	APIVYELNENDDNMNN	310	0.93
PF3D7_0529400	5	HMIPMDKNRFMDKGDV	634	0.92
PF3D7_0529400	5	NKKIEEHILSDELKNL	1115	0.92
PF3D7_0529400	6	YSDMEKHEDVRSYQNS	896	0.91
PF3D7_0529400	6	HGMFQSDEEYDNNNLY	785	0.91

Protein ID	Rank	Sequence	Start Position	Score
PF3D7_0529400	6	ADKIFAFDTSGENNNI	30	0.91
PF3D7_0529400	6	DDEKKVTDENDAIDTQ	1560	0.91
PF3D7_0529400	6	KDSIDYSQLMGQNNNA	1203	0.91
PF3D7_0529400	6	NLKIDRNVTERKRASI	1129	0.91
PF3D7_0529400	7	HENVINPLKEGYKNNE	841	0.90
PF3D7_0529400	7	YGIRKELIINDNNNNN	689	0.90
PF3D7_0529400	7	KKIIPQEYVNDINDNM	666	0.90
PF3D7_0529400	7	SGENNNIITNDKYINN	39	0.90
PF3D7_1435700	1	QEFITASKEFNEKLTY	378	0.97
PF3D7_1435700	2	SGYIENPIYYNPYMRN	757	0.94
PF3D7_1435700	2	ISHIAEKTKHNNANDP	424	0.94
PF3D7_1435700	2	DHSGMNQPYIDSQKNI	409	0.94
PF3D7_1435700	3	DNSIYSKDIFRIDADI	111	0.93
PF3D7_1435700	4	NNEIDEEDLFGAVRQN	229	0.92
PF3D7_1435700	5	MHSYMHSNINYAINNN	831	0.91
PF3D7_1435700	5	RGMHLDPEDQEKNNNE	216	0.91
PF3D7_1435700	6	YPMRNPHVVQNNHMNF	813	0.90
PF3D7_1435700	6	SSSIEENICANKKNKI	306	0.90
